# Intracellular and Extracellular Efficacy of Homoisoflavone Derivatives Against *Mycobacterium Tuberculosis*: Progress Toward Novel Antitubercular Agents

**DOI:** 10.1002/cmdc.202500249

**Published:** 2025-06-04

**Authors:** Sanderson D. Calixto, Juliane S. Falcão, Stella S. Antunes, Marlon H. Araujo, Alexandre L. B. Cunha, David R. Martins, Sarah M. R. Nascimento, Thatiana L. B. V. Simão, Elena B. Lasunskaia, Nelilma C. Romeiro, Paulo R. R. Costa, Michelle F. Muzitano, Guilherme S. Caleffi

**Affiliations:** ^1^ Laboratório de Produtos Bioativos Instituto de Ciências Farmacêuticas Universidade Federal do Rio de Janeiro Macaé Rio de Janeiro 27930‐560 Brazil; ^2^ Laboratório de Biologia do Reconhecer Centro de Biociências e Biotecnologia Universidade Estadual do Norte Fluminense Darcy Ribeiro Campos dos Goytacazes Rio de Janeiro 28013‐602 Brazil; ^3^ Laboratório de Química Bioorgânica Instituto de Pesquisas de Produtos Naturais Walter Mors Universidade Federal do Rio de Janeiro Rio de Janeiro 21941‐902 Brazil; ^4^ Laboratório Integrado de Computação Científica Instituto Multidisciplinar de Química Universidade Federal do Rio de Janeiro Macaé Rio de Janeiro 27930‐560 Brazil

**Keywords:** absorption, distribution, metabolism, excretion and toxicity, drug development, homoisoflavones, synthesis, tuberculosis

## Abstract

Tuberculosis (TB) remains a leading cause of death among infectious diseases globally, necessitating new drug discovery due to rising drug‐resistant strains. Homoisoflavones, a distinct subgroup of flavonoids characterized by their 3‐benzylidenechroman‐4‐one skeleton, are promising natural products for new antimicrobials. This study explored 42 homoisoflavone derivatives as potential anti‐TB agents. Several derivatives showed potent anti‐*Mycobacterium tuberculosis* (*Mtb*) activity. Specifically, derivatives **19**, **22**, and **41** show good selectivity index and significantly inhibited the *Mtb* H37Rv strain (MIC_90_ 2.2, 3.8, and 1.9 μM, respectively). Derivatives **22** and **41** were particularly effective against the hypervirulent clinical isolate *Mtb* M299 (MIC_90_ 1.5 and 2.5 μM, respectively), surpassing the potency of rifampicin (MIC_90_ 3.3 μM). Furthermore, these derivatives inhibited intracellular *Mtb* H37Rv growth in infected macrophages, with derivative **41** proving most potent (IC_50_ 5.2 μM) due to its unique nitrofuranyl and piperidine groups. The study also established a structure‐activity relationship (SAR) for the homoisoflavone scaffold. *In silico* analyses suggest these compounds have good oral bioavailability and low toxicity. These findings highlight homoisoflavone derivatives as promising candidates for future anti‐TB drug development.

## Graphical abstract

A series of 42 homoisoflavones were synthesized and evaluated against *Mycobacterium tuberculosis* (Mtb). Three derivatives showed potent inhibition of Mtb strains. Evaluation in infected macrophages revealed promising intracellular activity, where one derivative featuring a nitrofuranyl and piperidine group emerges as the most promising. These findings highlight the potential of homoisoflavones as anti‐TB drug candidates.

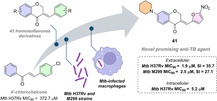

## 1 Introduction

In 2023, tuberculosis (TB) regained its position as the leading cause of death among infectious diseases worldwide, with ≈1.25 million fatalities, surpassing deaths attributed to COVID‐19.^[^ ^1^ ^]^ Approximately one third of the global population harbors a latent infection with *Mycobacterium tuberculosis* (*Mtb*), carrying a 5%–10% lifetime risk of reactivation.^[^ ^2^ ^]^ Although over 90% of infected individuals maintain the infection in a latent stage, they constitute a substantial reservoir for potential new cases of active TB.^[^ ^3^ ^]^

For over 60 years, TB treatment has been effectively standardized by the WHO, using anti‐TB drugs primarily targeting the causative *bacillus*. However, the emergence of drug resistance, largely driven by the selection of resistant strains and the high prevalence of TB‐HIV comorbidities, has led to the need for increasingly complex and side effect–prone therapeutic regimens, posing significant challenges to disease control in various regions.^[^ ^4^, ^5^ ^]^

One of the critical issues in TB control is the rise in resistance to rifampicin (RR‐TB), multidrug resistance (MDR), and extensively drug‐resistant TB (XDR). Between 2018 and 2022, an accumulated 825 000 individuals received treatment for resistant TB, with 675 489 cases of RR‐TB. This is particularly concerning as rifampicin is one of the most effective drugs against TB, and among these cases, 27 075 were classified as pre‐XDR‐TB or XDR‐TB.^[^ ^1^ ^]^ Success rates for RR‐TB treatment also reflect alarming data, with only 50%–75% efficacy, underscoring the need for ongoing research into safer and more effective anti‐TB agents.

Delays in initiating appropriate treatment or treatment failures due to bacterial resistance increase the severity of the disease, contributing to more cases of destructive pulmonary TB. Additional factors, such as the high virulence of infecting strains or host‐specific factors that enhance individual susceptibility to TB, further exacerbate disease progression and severity.^[^ ^2^, ^5^, ^6^ ^]^ Thus, continuing the discovery process for new compounds with high anti‐TB potency and low toxicity is crucial.

**Figure 1:**
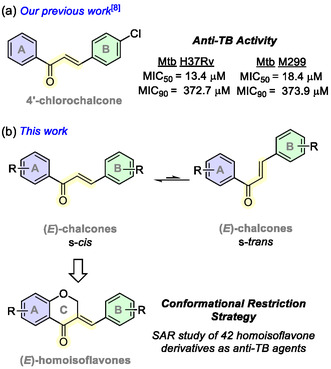
a) 4′‐Chlorochalcone, a promising anti‐TB agent. b) Homoisoflavones as rigid analogues of chalcones.

In this context, chalcones have gained recognition as a privileged scaffold for developing new bactericidal prototypes.^[^ ^7^ ^]^ Previously, our group conducted an in vitro study on the anti‐TB and anti‐inflammatory properties of a series of forty chalcones, examining their potential in a dual‐treatment approach. The 4'‐chlorochalcone (**Figure** **1**a) emerged as one of the most promising candidates, displaying pronounced inhibitory activity against the hypervirulent M299 strain.^[^ ^8^ ^]^ Building on our interest in synthesizing bioactive flavonoids and recognizing that chalcones serve as biosynthetic precursors to various flavonoid subclasses,^[^ ^9^ ^]^ we were prompted to investigate homoisoflavones—a unique subgroup of flavonoids—as rigid analogues of chalcones and explore their potential as anti‐TB agents (Figure 1b).

Homoisoflavones are sappanin‐type homoisoflavonoids with a 3‐benzylidenechroman‐4‐one skeleton.^[^ ^10^ ^]^ This flavonoid subgroup was first described in 1967 when eucomin was isolated from the bark of *Eucomis bicolor* BAK (Liliaceae).^[^ ^11^ ^]^ Their occurrence is reported in fewer species, mainly within the Liliaceae and Fabaceae families.^[^ ^12^, ^13^ ^]^ Due to their uncommon structure, the pharmacological activities remain under‐explored. Regarding antimicrobial properties, homoisoflavones from *Caesalpinia pulcherrina* (Fabaceae) were active against several bacteria, *Bacillus subtilis*, *B. sphaericus*, *Staphylococcus aureus*, *Klebisiella aerogenes*, and *Chromobacterium violaceum*, and the fungi *Aspergillus niger* and *Candida albicans*.^[^ ^14^ ^]^

To date, there are no reports on the antitubercular activity of natural homoisoflavones.^[^ ^10^, ^15^, ^16^, ^17^, ^18^ ^]^ However, antitubercular activity has been documented for natural isoflavones, especially prenylated ones like lupinifolin and lupinifolinol, with MIC ranging from 30 to 77 μM against *M. tuberculosis* H37Ra and H37Rv strains.^[^ ^19^, ^20^ ^]^ On the other hand, a 3‐benzyl‐4‐chromanone type homoisoflavanone isolated from *Chlorophytum inornatum* (Liliaceae) showed activity against fast‐growing *Mycobacterium* species, including *M. aurum* and *M. phlei*.^[^ ^21^ ^]^

A literature search for synthetic compounds with the 3‐benzylidene‐4‐chromanone scaffold evaluated against *Mtb* revealed only a single report. In this study, Dimmock and colleagues^[^ ^21^ ^]^ assessed 12 compounds against *Mtb* H37Rv strain, focusing primarily on monosubstituted derivatives on the B ring. Although the study scope was limited, it highlighted the largely untapped potential of the 3‐benzylidene‐4‐chromanone scaffold for developing anti‐TB agents.

Therefore, this work aims to design, synthesize, and evaluate the structure‐activity relationship (SAR) of an unprecedented set of 42 homoisoflavone derivatives as growth inhibitors of *Mtb* strains with differing virulence levels, testing both in bacterial cultures and in infected macrophages.

## 2 Results and Discussion

### 2.1 Design and Synthesis of the Compounds

**Figure 2:**
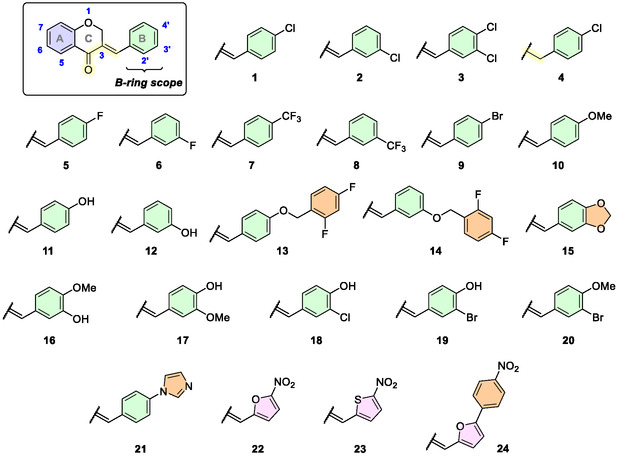
B‐ring substituted homoisoflavone derivatives evaluated as anti‐TB agents.

Based on the structure of the 4′‐chlorochalcone, we designed the homoisoflavone derivatives as their rigid analogues, starting by varying the substituents on the B‐ring (**Figure** **2**). Compounds **1**‐**4** are mono‐ or disubstituted with chlorine at the *para* and/or *meta* position of the B‐ring. The effect of the exocyclic double bond at the 3‐position of the C‐ring will also be evaluated through comparison with the benzyl analogue **4** (Figure 2).

Other mono‐substituted halogenated groups, such as fluorine, bromine, and trifluoromethyl, were also planned at *meta* or *para* positions (**5**‐**9**). The next group designed consists of mono‐ or disubstituted compounds with oxygenated substituents (**10**‐**20**). Within this group, the 2,4‐difluorobenzyloxyl (**13**, **14**) and methylenedioxyphenyl (**15**) moieties emerged as pharmacophoric groups in promising anti‐TB chalcone derivatives.^[^ ^8^, ^22^ ^]^ The nitrofuranyl (**22**) and nitrothiophenyl (**23**) moieties were also identified in the structure of promising anti‐TB hybrid chalcones.^[^ ^23^, ^24^ ^]^ These groups could act as prodrugs and could be activated by nitroreductases (Figure 2).^[^ ^25^ ^]^

**Figure 3:**
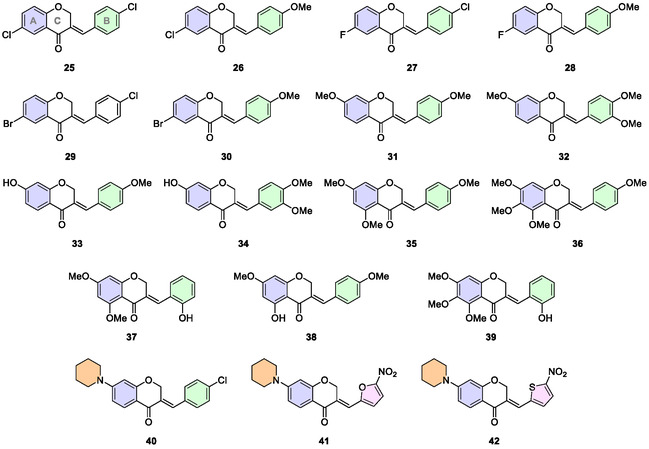
A‐ and B‐ring substituted homoisoflavone derivatives evaluated as anti‐TB agents.

The second set of planned compounds includes variations in the substituent patterns on both the A‐ and B‐rings (**Figure** **3**).

Compounds **25**‐**30** feature halogenated groups (Cl, F, and Br) at the 6‐position of the A‐ring and 4′‐Cl or 4′‐OMe groups at the B‐ring. In contrast, compounds **31**‐**39** exhibit typical oxygenated substitution patterns of natural flavonoids.^[^ ^10^, ^16^ ^]^ Among these, four compounds are natural homoisoflavones (**31**, **32**, **33**, **38**).^[^ ^14^, ^20^ ^]^ The last three compounds (**40**‐**42**) were designed to include a piperidine ring at the 7‐position of the A‐ring (Figure 3). This moiety has also been identified in the structure of anti‐TB hybrid chalcones.^[^ ^23^, ^24^ ^]^

**Scheme 1:**
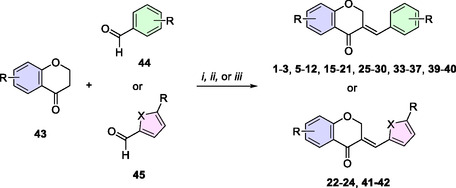
Synthesis of homoisoflavone derivatives through the aldol condensation reactions. *i*) HCl, MeOH, reflux, 12–24 h (**1**‐**3**, **5‐12**, **15‐21**, **23‐30**, **33**, **35‐36**, **39‐40**, **42**); *ii*) H_2_SO_4_, AcOH, 80–100 °C, 12–20 h (**22**, **37**, and **41**); *iii*) 60% KOH aq., MeOH, rt, 72 h (**34**).

The homoisoflavone derivatives were achieved via an aldol condensation reaction between chroman‐4‐ones (**43**) and aromatic (**44**) or heteroaromatic (**45**) aldehydes, serving as a key step. Most of the compounds were prepared using concentrated hydrochloric acid in methanol as the solvent, under reflux conditions, as illustrated in **Scheme** **1**.^[^ ^26^ ^]^ In specific cases, such as the synthesis of compounds **22**, **37**, and **41**, a sulfuric acid/acetic acid mixture was employed to improve yields.^[^ ^27^ ^]^ Alternatively, for the synthesis of compound **34**, a base‐catalyzed aldol condensation reaction was used.^[^ ^28^ ^]^ In total, twenty‐three compounds were synthesized in a single step through the aldol condensation reaction by the combination of chroman‐4‐ones (**43**) with aromatic (**44**) or heteroaromatic (**45**) aldehydes (Scheme 1).

**Scheme 2:**
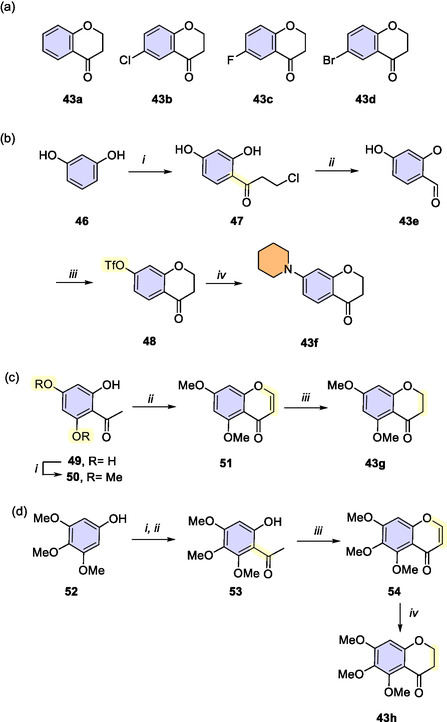
a) Commercially available chroman‐4‐ones (**43a**‐**d**). b) Synthesis of chroman‐4‐ones **43e** and **43f**. *i*) 3‐chloropropionic acid, TfOH, 80 °C, 1 h; *ii*) 2 M NaOH, rt, 2 h; *iii*) Tf_2_O, Py, rt, 5 h; *iv*) piperidine, DMSO, rt, 72 h. c) Synthesis of **43g**. *i*) (CH_3_)_2_SO_4_, K_2_CO_3_, acetone, reflux, 1 h; *ii*) DMF‐DMA, 110 °C, 3 h; *iii*) 10% Pd/C, H_2_, MeOH, rt, 2 h. d) Synthesis of **43h**. *i*) Ac_2_O, AcONa, 110 °C, 3 h; *ii*) BF_3_ OEt_2_, AcOH, 80 °C, 3 h; *iii*) DMF‐DMA, 110 °C, 3 h; *iv*) 10% Pd/C, H_2_, MeOH, rt, 2 h.

Eight chroman‐4‐ones (**43**) were employed in this study, with four of them (**43a**‐**d**) being commercially available (**Scheme** **2**a). The synthesis of the remaining four chroman‐4‐ones is outlined in Scheme 2b–d.

The 7‐hydroxychroman‐4‐one (**43e**) was synthesized in two steps from resorcinol (**46**), undergoing *C*‐acylation with 3‐chloropropionic acid under neat triflic acid conditions, followed by a base‐catalyzed intramolecular cyclization (Scheme 2b).^[^ ^26^ ^]^ Subsequent reaction of **43e** with triflic anhydride, followed by amination with piperidine, yielded chroma‐4‐one **43f** with a tertiary amine at 7‐position.^[^ ^29^ ^]^

For the synthesis of the 5,7‐dimethoxychroman‐4‐one (**43g**), partial methylation of 2,4,6‐trihydroxyacetophenone (**49**) with dimethyl sulfate^[^ ^30^ ^]^ was followed by reaction with *N*,*N*‐dimethylformamide dimethyl acetal (DMF‐DMA)^[^ ^31^ ^]^ to produce chromone **51**. Catalytic hydrogenation using Pd/C then yielded the desired chroman‐4‐one **43g** (Scheme 2c).^[^ ^32^ ^]^

Finally, a four‐step synthesis of 5,6,7‐trimethoxychroman‐4‐one (**43h**) began with the acetylation of 3,4,5‐trimethoxyphenol (**52**), followed by Fries rearrangement using BF_3_•OEt_2_ to produce 2‐hydroxyacetophenone **53**.^[^ ^33^ ^]^ This intermediate then underwent the same reaction sequence with DMF‐DMA and catalytic hydrogenation to afford **43 h** (Scheme 2d).

**Scheme 3:**
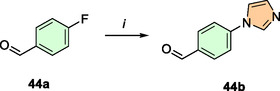
Preparation of the noncommercially available aldehyde **44b**. *i*) 1*H*‐imidazole, K_2_CO_3_, DMF,100 °C 24 h.

In this study, twenty‐three aldehydes were used, of which twenty‐two are commercially available. The 4‐(1*H*‐imidazol‐1‐yl)benzaldehyde (**44b**) was synthesized via an SNAr reaction between 4‐fluorobenzaldehyde (**44a**) and 1*H*‐imidazole (**Scheme** **3**).^[^ ^34^ ^]^

**Scheme 4:**
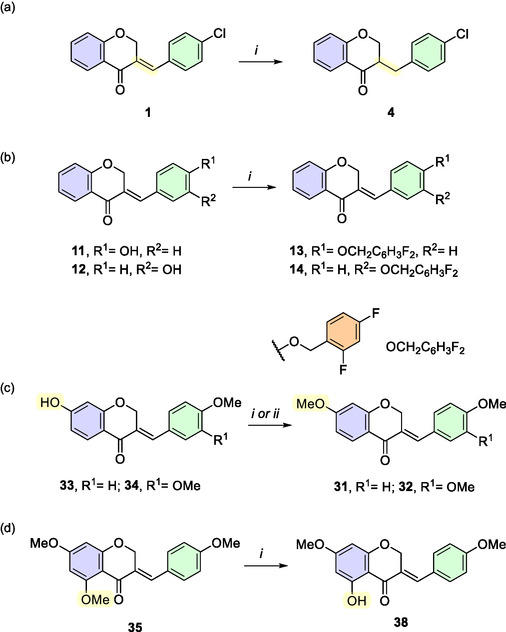
a) Catalytic hydrogenation of the C═C bond of compound **1**. *i*) 10% Pd/C, AcOEt, rt, 5 h. b) Benzylation reaction of the phenolic derivatives **11** and **12**. *i*) 2,4‐Difluorobenzyl bromide, K_2_CO_3_, CH_3_CN, 50 °C, 12 h. c) Methylation reactions of compounds **33** and **34**. *i*) Mel, K_2_CO_3_, acetone, reflux, 2 h (**31**); *ii*) Mel, K_2_CO_3_, DMF, 60 °C, 12 h (**32**). d) Selective demethylation of compound **35** with boron trichloride. i) BCl_3_, DCM, rt, 1 h.

Six of the forty‐two homoisoflavone derivatives (compounds **4**, **13**, **14**, **31**, **32**, and **38**) required an additional synthetic step following the aldol condensation reaction (**Scheme** **4**). Homoisoflavanone **4** was obtained by reducing the C=C exocyclic bond of compound **1** using 10% Pd/C under an H_2_ atmosphere yielded (Scheme 4a).^[^ ^35^ ^]^ Homoisoflavones **13** and **14** were obtained by reacting their respective phenolic precursors, **11** and **12**, with 2,4‐difluorobenzyl bromide in the presence of K_2_CO_3_ at 50 °C in CH_3_CN (Scheme 4b).^[^ ^36^ ^]^ Methylation of the hydroxyl groups at the 7‐position on the A‐ring of homoisoflavones **33** and **34** with methyl iodide produced compounds **31** and **32**, respectively (Scheme 4c).^[^ ^31^ ^]^ Finally, selective demethylation of compound **35** at the 5‐position on the A‐ring, using boron trichloride, yielded homoisoflavone **38** (Scheme 4d).^[^ ^37^ ^]^

### 2.2 Evaluation of a Homoisoflavone Series Against *Mycobacterium Tuberculosis* and Macrophage Viability

The forty‐two synthesized homoisoflavone derivatives (**1**–**42**) were assessed for their antitubercular activity against the *Mtb* H37Rv strain in an extracellular culture. The minimum concentrations needed for each compound to prevent 50% and 90% of bacterial growth (MIC_50_ and MIC_90_, respectively) were determined. The results are detailed in **Table** **1**.

**Table 1:** Inhibitory effects of homoisoflavone derivatives on the growth of *Mycobacterium tuberculosis* H37Rv strain in extracellular culture and cytotoxicity on RAW 264.6 macrophages.

Compound	*Mycobacterium tuberculosis* H37Rv growth inhibition MIC_50_ [μM]	*Mycobacterium tuberculosis* H37Rv growth inhibition MIC_90_ [μM]	Cytotoxicity on macrophages CC_50_ [μM]	SI [CC_50_/MIC_90_]
**1**	13.2 ± 0.13	>100	>100	–
**2**	>100	>100	33.6 ± 0.09	–
**3**	31.0 ± 0.07	>100	38.7 ± 0.10	–
**4**	4.2 ± 0.09	>100	18.3 ± 0.07	–
**5**	10.0 ± 0.15	>100	64.5 ± 0.26	–
**6**	33.5 ± 0.14	>100	>100	–
**7**	>100	>100	43.4 ± 0.11	–
**8**	>100	>100	14.5 ± 0.18	–
**9**	24.1 ± 0.16	>100	>100	–
**10**	1.7 ± 0.07	16.9 ± 0.07	>100	>5.9
**11**	3.4 ± 0.16	>100	67.2 ± 0.17	–
**12**	5.0 ± 0.07	>100	>100	–
**13**	58.4 ± 0.07	>100	>100	–
**14**	>100	>100	>100	–
**15**	3.0 ± 0.05	28.7 ± 0.05	78.3 ± 0.16	2.7
**16**	33.0 ± 0.17	>100	17.3 ± 0.05	–
**17**	5.3 ± 0.08	16.5 ± 0.08	47.7 ± 0.11	2.9
**18**	5.4 ± 0.09	18.8 ± 0.09	58.7 ± 0.10	3.1
**19**	**≤0.8**	**2.2 ± 0.01**	**65.3 ± 0.09**	**29.7**
**20**	12.0 ± 0.05	>100	42.8 ± 0.06	–
**21**	4.5 ± 0.13	25.6 ± 0.13	93.0 ± 0.10	3.6
**22**	**1.4 ± 0.04**	**3.8 ± 0.04**	**41.6 ± 0.05**	**10.9**
**23**	45.6 ± 0.12	>100	71.1 ± 0.04	–
**24**	>100	>100	80.6 ± 0.08	–
**25**	>100	>100	19.1 ± 0.07	–
**26**	>100	>100	46.3 ± 0.07	–
**27**	10.0 ± 0.15	>100	>100	–
**28**	33.5 ± 0.10	>100	>100	–
**29**	>100	>100	97.3 ± 0.09	–
**30**	>100	>100	>100	–
**31**	9.7 ± 0.14	>100	60.1 ± 0.05	–
**32**	3.5 ± 0.04	>100	89.5 ± 0.05	–
**33**	27.0 ± 0.05	>100	67.4 ± 0.05	–
**34**	2.5 ± 0.16	29.2 ± 0.16	>100	> 3.4
**35**	≤ 0.8	>100	30.4 ± 0.07	–
**36**	≤ 0.8	>100	48.1 ± 0.09	–
**37**	≤0.8	>100	60.6 ± 0.06	–
**38**	≤0.8	>100	12.7 ± 0.09	–
**39**	≤0.8	>100	21.5 ± 0.04	–
**40**	>100	>100	67.9 ± 0.08	–
**41**	≤0.8	**1.9 ± 0.08**	**67.8 ± 0.04**	**35.7**
**42**	≤0.8	30.1 ± 0.11	56.3 ± 0.11	1.9
**Rifampicin** a)	0.2 ± 0.10	1.5 ± 0.08	–	–

a) Standard antimycobacterial drug; mean value ± SD; *n* = 3; ‐ not defined.

### 2.3 SAR Evaluation

To further investigate the physicochemical properties potentially linked to the observed in vitro activity of the synthesized compounds (Table 1), we calculated several steric and electronic structural descriptors (Table S1, Supporting Information). Our goal is to establish unprecedent SARs for the homoisoflavone scaffold against *Mtb* H37Rv strain.

Comparing the 4′‐chlorohomoisoflavone (**1**) with the 4′‐chlorochalcone from our previous work (Figure 1a),^[^ ^8^ ^]^ we observed the beneficial effect of the conformational restricted *α,β*‐unsaturated ketone system of homoisoflavone [MIC_50_ = 13.4 μM (4′‐chlorochalcone) vs. 1.7 μM (**1**)].

The homoisoflavones substituted with the chlorine group at the 3′‐position of the B‐ring (**2**) or with two chloride groups at the 3′‐ and 4′‐positions (**3**) showed a significant decrease in *Mtb* growth inhibition compared to **1**. Despite homoisoflavanone **4** exhibited a lower MIC_50_ value than its unsaturated counterpart **1**, the MIC_90_ determination indicated neither compound was promising, with MIC_90_ exceeding 100 μM. Other halogenated groups, such as fluorine (**5**‐**6**), trifluoromethyl (**7**‐**8**), and bromine (**9**), were evaluated but did not show higher potency than compound **1** (Table 1). The compounds containing the trifluoromethyl group (**7**‐**8**) exhibited the highest MIC_50_ among the halogenated. According to our SAR analysis, these compounds also have the highest molecular surface area and volume values (Table S1, Supporting Information), which may have hindered binding to the biological target due to steric hindrance. Additionally, compounds **7**‐**8** show the lowest calculated HOMO and LUMO energy values. Since HOMO and LUMO energies can critically impact ligand‐protein interactions due to their electron donor/acceptor capabilities, they are useful descriptors for evaluating the effects of different substituents on new compounds.^[^ ^38^ ^]^

Among the B‐ring mono‐oxygenated substituted compounds (**10**‐**14**), the 4′‐methoxyl group (**10**) emerged as the most promising, with a MIC_50_ of 1.7 μM outperforming hydroxyl or 2,4‐difluorobenzyloxyl groups at the 4′‐ or 3′‐positions. Compound **10** has the highest calculated HOMO energy value (Table S1, Supporting Information), indicating a strong propensity for electron donation to a suitable acceptor, such as a biological target binding site. Furthermore, the lowest HOMO‐LUMO gap value can be related to high chemical and biological reactivities/activities, albeit with relatively lower kinetic stability.^[^ ^39^ ^]^

The homoisoflavone **15**, bearing a methylenedioxy group on the B‐ring, was identified as a promising anti‐TB agent, with a MIC_50_ of 3.0 μM and a MIC_90_ of 28.7 μM (Table 1). Notably, in our previous work,^[^ ^8^ ^]^ this substitution pattern was found to be one of the most effective for the chalcone scaffold, with a MIC_50_ of 12.7 μM and a MIC_90_ of 303.2 μM against the *Mtb* H37Rv strain. This data further emphasizes the higher potency of the homoisoflavone skeleton compared to chalcone in anti‐TB activity.

B‐ring disubstituted homoisoflavones, bearing either two or one oxygenated groups and one halogen group, were also evaluated (**16**‐**20**). Compound **19**, featuring a hydroxyl group at the 4′‐position and a bromine at the 3′‐position, was the most potent, with a MIC_50_ of 0.04 μM and a MIC_90_ of 2.2 μM against *Mtb* H37Rv strain. The analyses of the 2D structure suggest that the presence of a hydroxyl group at the 4′‐position and a less electronegative, more ionizable atom at the 3′‐position are important for antimycobacterial activity.

Compound **21**, substituted with a pharmacophoric anti‐TB imidazolyl group^[^ ^22^ ^]^ at the 4′‐position of the B‐ring, exhibited moderate activity against *Mtb* H37Rv strain with MIC_90_ of 25.6 μM. The imidazole ring is known for its wide range of biological effects, including antibacterial properties.^[^ ^40^, ^41^ ^]^ However, among the planned pharmacophoric groups, the nitrofuranyl ring demonstrated better activity, with a MIC_90_ of 3.8 μM for compound **22**. Interestingly, substituting oxygen with sulfur in the nitrothiophenyl derivative **23**, or introducing an aryl group as a spacer between the nitro group and the furan ring (**24**), resulted in a significant decrease in the anti‐TB activity, MIC_90_ of 45.6 μM and > 100 μM, respectively (Table 1). This outcome aligns with existing trends in the literature, as nitrofuran compounds are well‐documented for their remarkable antimycobacterial activity.^[^ ^42^ ^]^

Next, we evaluated chlorine, fluorine, and bromine substitutions at the 6‐position of the A‐ring, combined with either chlorine or methoxyl at the 4′‐position of the B‐ring (compounds **25**‐**30**). In all cases, these modifications led to a decrease in anti‐TB activity compared to the corresponding A‐ring nonsubstituted derivatives **1** and **10**. None of the compounds (**25**‐**30**) achieved a MIC_90_ < 100 μM.

The effects of methoxyl or hydroxyl groups at the A‐ring 7′‐position were also evaluated in compounds **31**‐**34**, which contain one or two methoxyl groups on the B‐ring. Among these compounds, the most active was **34,** with MIC_90_ of 29.2 μM, which has the highest polar surface area (PSA) value of 65 Å^2^ (Table S1, Supporting Information). This descriptor is related to drug transport properties, and compounds with PSA higher than 140 Å^2^ are believed to be less capable of penetrating cell membranes.^[^ ^43^ ^]^ Since the PSA value observed for **34** is below 140 Å^2^, it might be lying in a better range compared to **31**‐**33**. Additionally, other di‐ and trisubstituted oxygenated patterns, commonly found in natural flavonoids,^[^ ^44^, ^45^, ^46^, ^47^ ^]^ were tested but demonstrated low potency against *Mtb* H37Rv growth (compounds **35**‐**39**, MIC_90_ > 100 μM).

The final three compounds (**40**‐**42**) contain a piperidine ring at the 7‐position of the A‐ring. Compound **40** demonstrated a notable decrease in potency compared to the nonsubstituted A‐ring homoisoflavone **1** [MIC_50_ = >100 μM (**40**) vs 1.7 μM (**1**)]. However, a synergistic effect on activity was observed in homoisoflavone derivatives featuring nitro‐substituted heterocyclic B‐rings and piperidine groups at the A‐ring. Compound **41**, which incorporates a nitrofuranyl group as the B‐ring, exhibited the highest anti‐TB activity in the series, with a MIC_90_ of 1.9 μM, twice as potent as its corresponding nonsubstituted A‐ring analog (compound **22**, MIC_90_ of 3.8 μM), showing that the addition of a piperidine ring is important to increase the activity of compound **41**. The analysis of the descriptors demonstrated that the most active compound has the highest dipole moment calculated value (Table S1, Supporting Information). This descriptor is useful for predicting drug‐receptor interactions, and polar compounds are associated with the facilitation of forming hydrogen bonding contacts.^[^ ^48^ ^]^

In summary, ten homoisoflavones demonstrated a promising inhibitory effect on *Mtb* growth, with MIC_90_ values below 30 μM: compounds **10**, **15**, **17‐19**, **21**, **22**, **34**, **41**, and **42** (Table 1). Among the most active compounds, all exhibit intermediate lipophilicity, with Log P values in the range 1.3‐4.1 (Table S1, Supporting Information). We observed that all the compounds with Log P values higher than 4.1 had MIC_90_ values exceeding 100 μM. However, compound **42** is an exception in this series, with a Log P value of 4.41, which may be correlated with other physicochemical properties, leading to optimum results in *Mtb* growth inhibition.^[^ ^49^ ^]^ The most active compounds against *Mtb* H37Rv strain were the homoisoflavones **19**, **22**, and **41**, with MIC_90_ values of 2.2, 3.8, and 1.9 μM, respectively.

Additionally, the cytotoxicity of all forty‐two homoisoflavone derivatives was assessed using RAW 264.7 macrophage viability after treatment at various concentrations. The calculated CC_50_ (the concentration of the samples required to reduce cell viability by 50%) ranged from 12.7 to > 100 μM. The selectivity index (SI) for the ten most promising homoisoflavones, calculated as CC_50_/MIC_90_, ranged from 2.7 to 35.7 (Table 1).

Based on the efficacy and selectivity data from this screening, compounds **19**, **22**, and **41**, which displayed low cytotoxicity (SI ≥ 10) and strong activity against *Mtb* H37Rv strain, were selected for further investigation against intracellular *Mtb*.

### 2.4 Effect of the Promising Homoisoflavones Derivatives on Intracellular Growth of Mtb

The challenge in the development of new drugs for tuberculosis lies in targeting compounds that effectively act against Mtb in various physiological states throughout the course of the infection. This complexity is driven by the pathogen's diverse resistance mechanisms and its adaptation to different host conditions.^[^ ^50^ ^]^

**Figure 4:**
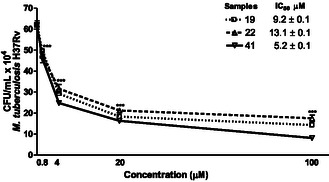
Effect of homoisoflavone derivatives on the intracellular growth of *Mycobacterium tuberculosis* (*Mtb*) H37Rv in macrophages. RAW 264.7 macrophages were infected with *Mtb* H37Rv at an MOI of 1:1 (bacterium per macrophage) and treated with compounds **19**, **22,** and **41**(0.8, 4, 20, and 100 μM) for 4 days. To measure intracellular bacterial growth, the cells were lysed on day 4 and plated in Middlebrook 7H10 agar. After 21 days, bacterial colonies were counted (CFU*U* test). The mean value of each group, significantly different from the mean value of the positive control (untreated infected macrophages, 62.0 ± 1.0 × 10^4^ CFU/mL), was indicated by asterisks according to p < 0.001 (***), p < 0.01 (**) and p < 0.05 (*) (*n* = 3). The calculated IC_50_ (half‐maximal inhibitory concentration) of homoisoflavones on the intracellular growth of *Mtb* was inserted next to the graph.

The results presented in **Figure** **4** show that the three selected homoisoflavones significantly suppressed the intracellular growth of *Mtb* H37Rv strain, reducing colony‐forming units (CFU) by 60 to 69% even at a concentration of 20 μM. Homoisoflavones **19**, **22**, and **41** exhibited significant IC_50_ values, with compound **41** showing the strongest inhibition of intracellular *Mtb* growth, achieving an IC_50_ of 5.2 μM.

### 2.5 Evaluation of Absorption, Distribution, Metabolism, Excretion, and Toxicity (ADMET) Parameters of the Most Active Compounds

Poor pharmacokinetic properties and safety of bioactive compounds are still the major challenges in drug discovery.^[^ ^51^ ^]^ In silico predictive ADMET models have been used to accelerate the identification of safety and effective compounds based on their chemical structure.^[^ ^52^ ^]^ Therefore, we evaluated the theoretical ADMET properties of the most active anti‐TB compounds (**Table** **2**). The first assessed parameters were human intestinal absorption (HIA), Caco‐2 cell permeability, and oral bioavailability based on Lipinski's rule of five. All the compounds showed good predicted intestinal absorption through the intestinal epithelial barrier and passed Lipinski's rule,^[^ ^53^ ^]^ showing good predicted oral bioavailability. To explore the potential toxicity of the synthesized compounds, some risks as cardiotoxicity based on hERG channel inhibition, hepatotoxicity, and Ames mutagenicity, were predicted (Table 2). The most promising homoisoflavones, compounds **19**, **22,** and **41**, did not show predicted cardiotoxicity. Compound **19** also did not demonstrate potential mutagenicity. All the compounds evaluated, including rifampicin, were predicted as hepatotoxic.

**Table 2:** In silico ADMET properties of the most active homoisoflavones derivatives and rifampicin.

Compound^a^	HIAa)	Caco‐2	hERG inhibitor	Hepatotoxicity	Mutagenicity	Rule of 5
10	Good	Good	Yes (93.7%)	Yes (76.6 %)	Yes (58.8%)	Pass
15	Good	Good	Yes (87.5%)	Yes (74.6%)	Yes (60.9%)	Pass
17	Good	Good	No	Yes (65.3%)	No	Pass
18	Good	Good	No	Yes (60.4%)	No	Pass
19	Good	Good	No	Yes (67.9%)	No	Pass
21	Good	Good	Yes (97.2%)	Yes (65.3%)	Yes (59.4%)	Pass
22	Good	Good	No	Yes (92.2%)	Yes (95.8%)	Pass
34	Good	Good	No	Yes (63.7%)	No	Pass
41	Good	Good	No	Yes (91.9%)	Yes (95.7%)	Pass
42	Good	Good	Yes (63%)	Yes (88.5%)	Yes (81.9%)	Pass
Rifampicin	Good	Good	No	Yes (92.1%)	Yes (78.5%)	No

a) HIA: human intestinal absorption.

### 2.6 Homoisoflavones Activity Against the Hypervirulent Strain *Mycobacterium Tuberculosis* M299

To further evaluate the antimycobacterial potential of homoisoflavones, the ten most active compounds previously identified against the Mtb H37Rv strain (MIC_90_ < 30 μM) were tested against a highly virulent clinical isolate, Mtb M299 strain, which belongs to the modern Beijing sublineage. This hypervirulent strain is known for its rapid intracellular growth, induction of necrotic cell death in infected macrophages, and the formation of extensive necrotic lung lesions, accompanied by an exacerbated inflammatory response in infected C57BL/6 mice.^[^ ^54^, ^55^, ^56^ ^]^

As shown in **Table** **3**, all ten homoisoflavones demonstrated high activity against the hypervirulent strain (MIC_90_ < 30 μM). Compounds **22**, **34**, and **41** were particularly effective, with MIC_90_ 1.5, 4.2, and 2.5 μM, respectively, and favorable SI (>20). Notably, compounds **22** and **41**, both containing a nitrofuranyl group, achieved MIC_90_ values lower than that of rifampicin (MIC_90_ of 3.3 μM), the standard antitubercular drug.

**Table 3:** Inhibitory effects of promising homoisoflavone derivatives on the growth of the hypervirulent strain *Mycobacterium tuberculosis* M299 in culture and calculated selective index.

Promising compounds	*Mycobacterium tuberculosis* M299 growth inhibition MIC_90_ [μM]	SI [CC_50_/MIC_90_]a)
**10**	29.6 ± 0.06	> 3.4
**15**	22.7 ± 0.05	3.5
**17**	28.1 ± 0.04	2.1
**18**	28.4 ± 0.04	2.1
**19**	15.3 ± 0.04	4.3
**21**	19.5 ± 0.05	4.8
**22**	**1.5 ± 0.11**	**27.7**
**34**	**4.2 ± 0.06**	**23.8**
**41**	**2.5 ± 0.16**	**27.1**
**42**	10.3 ± 0.16	5.5
**Rifampicin** b)	3.3 ± 0.08	–

a) The CC50 presented in Table 2 were used to calculate the SI on Mtb M299.

b) Standard antimycobacterial drug; mean value ± SD; *n* = 3; ‐ not defined.

## 3 Conclusions

In this study, we investigated the potential of homoisoflavone derivatives as effective anti‐TB agents. Homoisoflavones, a unique and largely underexplored subgroup of flavonoids characterized by a 3‐benzylidenechroman‐4‐one skeleton, were designed as rigid analogues of chalcones, incorporating various halogenated, oxygenated, and heterocyclic substituents, including pharmacophoric groups identified in previously promising anti‐TB chalcone derivatives.

A total of 42 homoisoflavone derivatives were synthesized through a one‐ to five‐step process, with aldol condensation as the key synthetic step, and subsequently evaluated as growth inhibitors of *Mtb* H37Rv. The ten most active compounds (**10**, **15**, **17**‐**19**, **21**, **22**, **34**, **41**, and **42**) demonstrated MIC_90_ values below 30 μM, indicating significant inhibitory activity. Through calculated steric and electronic structural descriptors, we established an unprecedented SAR for the homoisoflavone scaffold, confirming the enhanced potency of these compounds as conformationally restricted analogues of chalcones.

Among these, homoisoflavone **15**, featuring a methylenedioxy group on the B‐ring, showed a tenfold improvement in potency (MIC_90_ of 28.7 μM) compared to its corresponding chalcone analogue (MIC_90_ of 303.2 μM). Notable activity was also observed for compound **19**, with a hydroxyl group at the 4′‐position and a bromine at the 3′‐position (MIC_90_ of 2.2 μM), and compound **22**, bearing a nitrofuranyl group as the B‐ring (MIC_90_ = 3.8 μM). The synergistic effects of combined substituents were exemplified by compound **41**, featuring the nitrofuranyl group as B‐ring and the piperidine group at the 7‐position of A‐ring, achieving the highest potency in the series (MIC_90_ = 1.9 μM). It is important to mention that this is the first time that these most active homoisoflavones are being described.

Based on efficacy (MIC_90_ < 5 μM) and selectivity (SI ≥ 10), compounds **19**, **22**, and **41** were further tested against intracellular growth of *Mtb* H37Rv in macrophages. Homoisoflavone derivative **41** emerged as the most effective, with an IC_50_ of 5.2 μM and a SI of 13.

Additionally, the ten most active compounds were evaluated against the highly virulent *Mtb* M299 strain. Compounds **22** and **41**, both containing a nitrofuranyl group, displayed MIC_90_ values of 1.5 and 2.5 μM, respectively, surpassing the potency of rifampicin (MIC_90_ of 3.3 μM), the current standard antitubercular drug.

In summary, our findings highlight homoisoflavone derivatives as promising novel anti‐TB agents. Further investigations into their mechanisms of action are underway in our laboratories.

## 4 Experimental Section

4.1

4.1.1

##### Synthesis of the Compounds: General Experimental Procedures

All commercial reagents and solvents were purchased from Sigma‐Aldrich, TCI, Oakwood Chemical, or Acros Organics and used without further purification. All reactions that required heating were performed using an oil bath. Analytical thin layer chromatography was performed on 0.25 mm silica gel 60 F_254_ plates and visualized under UV light (254 nm or 365 nm) or by staining with vanillin/H_2_SO_4_. Flash column chromatography was performed on silica gel 60 (230–400 mesh) SiliaFlashTM. High‐performance liquid chromatography (HPLC) analyses were carried out on a Shimadzu LC‐20AT liquid chromatography equipped with an SPD‐M20A diode array detector, and retention times (*t*_R_) were expressed in minutes. All tested compounds had purity ≥ 95% determined by HPLC analysis. NMR spectra were recorded on Varian Unity 400 or 500 MHz instruments at 25 °C. Chemical shifts were expressed in ppm relative to TMS (Me_4_Si) or deuterated solvent (CDCl_3_, DMSO‐*d*_*6*_, Acetone‐*d*_6_), and the coupling constants were expressed in Hz. High‐resolution mass spectra were obtained with a Solarix XR mass spectrometer with electrospray ionization source coupled to Fourier transform‐ion cyclotron resonance mass analyzer. The characterization data of each synthesized intermediates and final products [purification method, isolated yield, purity by HPLC analysis (column, mobile phase, flow, and t_R_), and spectroscopic data] are described in the Supporting Information file.

##### Procedures for the Synthesis of the Starting Materials

The syntheses of the acetophenones (**50**, **53**), 4*H*‐chromen‐4‐ones (**51**, **54**), chroman‐4‐ones (**43e‐h, 48**), and aldehyde **44b** that were not obtained from commercial sources are described in the following.

##### Procedure for the Synthesis of 1‐(2‐Hydroxy‐4,6‐Dimethoxyphenyl)ethan‐1‐One (50)

In a two‐neck flask, 1‐(2,4,6‐trihydroxyphenyl)ethan‐1‐one (10 mmol, 1.861 g, 1 equiv) and potassium carbonate (20 mmol, 1.764 g, 2 equiv) were added and dissolved in acetone (50 mL). The mixture was refluxed with stirring for 20 min. Then, dimethyl sulfate (20 mmol, 1.89 mL, 2 equiv) was added while hot, and the reaction was continued under reflux for an additional hour. After cooling to room temperature, cold water was added to the solution until complete precipitation occurred. The precipitate was filtered using a Büchner funnel and washed with cold water (5 × 100 mL), yielding the pure product **50**.

##### Procedure for the Synthesis of 1‐(6‐Hydroxy‐2,3,4‐Trimethoxyphenyl)ethan‐1‐One (53)

*Step* 1. A mixture of 3,4,5‐trimethoxyphenol (10 mmol, 1.842 g, 1 equiv) and sodium acetate (22.6 mmol, 1.854 g, 2.26 equiv) in acetic anhydride (5 mL) was heated at 110 °C for 3 h. Subsequently, the mixture was concentrated under reduced pressure, diluted with water (100 mL), and extracted with dichloromethane (3 × 100 mL). The organic layers were combined and washed with water (250 mL × 5), dried over Na_2_SO_4_, filtered, and concentrated under reduced pressure, yielding 3,4,5‐trimethoxyphenyl acetate. *Step* 2. A mixture of 3,4,5‐trimethoxyphenyl acetate (10 mmol, 2.262 g, 1 equiv) and BF_3_·Et_2_O (36 mmol, 4.5 mL, 3.6 equiv) in acetic acid (2.26 mL) was stirred at 70 °C for 3 h. After cooling to room temperature, 10% NaOH (100 mL) was added, and the solution was extracted with diethyl ether. The aqueous phase was cooled, acidified with concentrated HCl, and extracted with dichloromethane. The organic layers were combined, dried over anhydrous sodium sulfate, and evaporated under reduced pressure, yielding 1‐(6‐hydroxy‐2,3,4‐trimethoxyphenyl)ethan‐1‐one without the need for further purification.

##### General Procedure for the Synthesis of 4H‐Chromen‐4‐ones 51 and 54

A pressure tube containing the acetophenone of interest (5.0 mmol, 1 equiv) and DMF‐DMA (7.5 mmol, 0.99 mL, 1.5 equiv) was heated at 115 °C for 3 h. Then, 5 mL of 37% HCl was added, and the mixture was heated at 50 °C for 40 min. After this, the reaction mixture was transferred to a separatory funnel using ethyl acetate (200 mL) and washed with water (200 mL × 2). The organic phase was dried over anhydrous sodium sulfate, filtered, and concentrated under reduced pressure, yielding the pure products **51** and **54**.

##### Procedure for the Synthesis of 7‐Hydroxy‐Chromanon‐4‐One (43e)

*Step* 1. The trifluoromethanesulfonic acid (24 mmol, 2.12 mL, 3 equiv) was added to a mixture of Resorcinol (8 mmol, 881 mg, 1 equiv) and 3‐chloropropionic acid (9.6 mmol, 1.042 g, 1.2 equiv). The resulting solution was stirred at 80 °C for 1 h, cooled to room temperature over 10 min, and poured into chloroform (50 mL). Then, the solution was washed with water (50 mL × 2), and the aqueous layer was extracted with chloroform (25 mL × 2). The combined organic layers were washed with brine, dried over Na_2_SO_4_, filtered, and concentrated under reduced pressure. The resulting product 3‐chloro‐1‐(2,4‐dihydroxyphenyl) propan‐1‐one was used crude in the next step. *Step* 2. A NaOH solution (2 M, 40 mL) was added to a round‐bottom flask containing 3‐chloro1‐(2,4‐dihydroxyphenyl) propan‐1‐one (4.5 mmol, 903 mg), and the resulting mixture was left under stirring at rt for 2 h. Then, a H_2_SO_4_ solution (6 M) was added until pH < 2, and the mixture was extracted with AcOEt (60 mL × 2). The resulting organic phase was washed with brine (120 mL), dried over Na_2_SO_4_, and concentrated under reduced pressure, resulting in 7‐hydroxy‐chroman‐4‐one that was used without further purification in the following step.

##### Procedure for the Synthesis of 4‐Oxochroman‐7‐yl Trifluoromethanesulfonate (48)

To a stirred solution of 7‐hydroxychroman‐4‐one (3 mmol, 524 mg, 1 equiv) in pyridine (2.4 mL) at 0 °C, trifluoromethanesulfonic anhydride (5.4 mmol, 1.02 mL, 1.8 equiv) was slowly added. The reaction was then left at room temperature for 3 h. Subsequently, the mixture was diluted with EtOAc (10 mL) and transferred to a separatory funnel, where the organic phase was washed with 2M HCl (2 × 70 mL), distilled water (2 × 70 mL), and saturated NaCl solution (70 mL). The organic phase was dried over anhydrous sodium sulfate, filtered, and evaporated under reduced pressure. Purification was carried out using flash silica column chromatography with AcOEt/Hex mixture.

##### Procedure for the Synthesis of 7‐(piperidin‐1‐yl)chroman‐4‐one (43f)

To a stirred solution of 4‐oxochroman‐7‐yl trifluoromethanesulfonate (2 mmol, 588 mg, 1 equiv) in DMSO (20 mL), piperidine was added (10 mmol, 1 mL, 5 equiv) and stirred at room temperature for 3 days. Subsequently, the reaction mixture was extracted with EtOAc (100 mL) and washed with water (150 mL × 8) and aqueous NaCl (150 mL × 2). The organic phase was dried over anhydrous sodium sulfate, filtered, and evaporated under reduced pressure, yielding the product **43f** as an orange solid, without the need for further purification.

##### General Procedure for the Synthesis of Chroman‐4‐ones 43g‐h

In a flask, the chroman‐4‐one of interest (4 mmol) and 10% Pd/C (12% w/w) were added. The mixture was dissolved in 20 mL of MeOH and stirred under a hydrogen atmosphere (1 atm) at room temperature for 1.5 h. Following this, a filtration through celite with ethyl acetate was performed, yielding the pure products **43g‐h.**

##### Procedure for the Synthesis of 4‐(1H‐Imidazol‐1‐yl)benzaldehyde (44b)

To a solution of 4‐fluorobenzaldehyde (2 mmol, 248 mg, 0,22 mL, 1 equiv) in DMF (20 mL), K_2_CO_3_ (4 mmol, 552 mg, 2 equiv) and 1*H*‐imidazole (12 mmol, 816 mg, 6 equiv.) were added and stirred at 100 °C for 24 h. The reaction was then cooled to room temperature and then diluted with water. Subsequently, the reaction mixture was diluted with water, extracted with EtOAc (100 mL) and the resulting organic phase was washed with water (150 mL × 8) and aqueous NaCl (150 mL × 2). The organic phase was dried over anhydrous sodium sulfate, filtered, and evaporated under reduced pressure. Purification was carried out using flash silica column chromatography with AcOEt/Hex mixture.

##### General Procedure for the Synthesis of Homoisoflavone Derivatives 1‐3, 5‐12, 15‐21, 23‐30, 33, 35‐36, 39‐40, and 42

The 4‐chromanone of interest (1.0 mmol) was dissolved in methanol (6 mL), followed by the addition of the corresponding aldehyde (1.05 mmol) and 37% HCl (3 mL). The mixture was refluxed for 24 h and then diluted with water. The reaction mixture was transferred to a separatory funnel with ethyl acetate and washed with saturated NaHCO_3_ solution (1 × 100 mL). The aqueous phase was extracted with ethyl acetate (3 × 100 mL). The combined organic fractions were washed with saline solution (1 × 100 mL), dried over anhydrous sodium sulfate, filtered, and concentrated under reduced pressure. Purification was carried out using flash silica column chromatography with AcOEt/Hex mixture.

##### General Procedure for the Synthesis of Homoisoflavone Derivatives 22, 37, and 41

To a stirred solution of the chromanone of interest (1 mmol, 1 equiv) in acetic acid (5 mL), the corresponding aldehyde (2 mmol, 2 equiv.) and concentrated sulfuric acid (5 mmol, 276 μL, 5 equiv) were added. The reaction mixture was heated at 80 °C for 12 to 20 h. Subsequently, it was diluted with 25 mL of ice‐cold distilled water, and the precipitate was filtered using a Büchner funnel. The nonprecipitated products had their pH adjusted to 5 and were extracted with EtOAc. The organic phase was washed with NaCl solution, dried over anhydrous sodium sulfate, filtered, and concentrated under reduced pressure. Purification was carried out using flash silica column chromatography with AcOEt/Hex mixture.

##### Procedure for the Synthesis of (E)‐3‐(3,4‐Dimethoxybenzylidene)‐7‐Hydroxychroman‐4‐one (34)

7‐Hydroxychroman‐4‐one (3 mmol, 492 mg, 1 equiv) was dissolved in methanol (10,7 mL), followed by the addition of 3,4‐dimethoxybenzaldehyde (3.6 mmol, 610 mg, 1.2 equiv) and 60% aqueous KOH solution (48 mmol, 4,5 mL, 16 equiv). The mixture was stirred at room temperature for 72 h. Subsequently, the reaction was acidified to pH 4. The precipitate was filtered with cold distilled water and recrystallized from hot methanol, yielding the pure product **34**.

##### Procedure for the Synthesis of 3‐(4‐Chlorobenzyl)chroman‐4‐one (4)

In a flask, the (*E*)‐3‐(4‐chlorobenzylidene)chroman‐4‐one (0.48 mmol, 130 mg) and 10% Pd/C (10% w/w, 13 mg) were added. The mixture was dissolved in 5 mL of MeOH and stirred under a hydrogen atmosphere (1 atm) at 0 °C for 45 min. Following this, a filtration through celite with ethyl acetate was performed and the organic phase was concentrated under reduced pressure. Purification was carried out using flash silica column chromatography with AcOEt/Hex mixture.

##### General Procedure for the Synthesis of Homoisoflavone Derivatives 13 and 14

To a stirred solution of compound **11** or **12** (0.2 mmol, 50 mg, 1 equiv) and K_2_CO_3_ (0.3 mmol, 41 mg, 1.5 equiv) in acetonitrile (1 mL), 1‐(bromomethyl)‐2,4‐difluorobenzene (0.3 mmol, 38 μL, 1.5 equiv) was added. The reaction was heated at 50 °C for 12 h. Subsequently, the reaction mixture was transferred to a separatory funnel using ethyl acetate, washed with 2 M KOH solution (1 × 30 mL) and NaCl solution (1 × 30 mL). The organic phase was dried over anhydrous sodium sulfate, filtered, and concentrated under reduced pressure. Purification was carried out using flash silica column chromatography with AcOEt/Hex mixture.

##### Procedure for the Synthesis of (E)‐7‐Methoxy‐3‐(4‐Methoxybenzylidene)chroman‐4‐one (31)

To a solution of (*E*)‐7‐hydroxy‐3‐(4‐methoxybenzylidene)chroman‐4‐one (1 mmol, 282 mg, 1 equiv) in acetone (5 mL) at rt were added iodomethane (3 mmol, 0.19 mL, 3 equiv) and K_2_CO_3_ (3 mmol, 415 mg, 3 equiv). The mixture was refluxed for 2 h; then, the acetone was evaporated, and the crude was diluted in AcOEt (25 mL). The mixture was washed with water (25 mL) and brine (25 mL), dried over Na_2_SO_4_, and concentrated under reduced pressure, resulting in **31.**

##### Procedure for the Synthesis of (E)‐3‐(3,4‐Dimethoxybenzylidene)‐7‐Methoxychroman‐4‐one (32)

To a mixture of (*E*)‐3‐(3,4‐dimethoxybenzylidene)‐7‐hydroxychroman‐4‐one (2 mmol, 624 mg, 1 equiv) and K_2_CO_3_ (2.4 mmol, 331 mg, 1.2 equiv) in DMF (2.7 mL), iodomethane (3 mmol, 0.19 mL, 1.5 equiv) was added, and the mixture was stirred at 60 °C for 12 h. The reaction mixture was extracted with ethyl acetate and washed with water (5 × 150 mL) and aqueous NaCl (2 × 150 mL). The organic phase was dried over Na_2_SO_4_, filtered and concentrated under reduced pressure, yielding the pure product **32**.

##### Procedure for the Synthesis of (E)‐5‐Hydroxy‐7‐Methoxy‐3‐(4‐Methoxybenzylidene)chroman‐4‐one (38)

The compound was added to a two‐neck flask (1 mmol, 163 mg, 1 equiv) and dissolved in anhydrous dichloromethane (15 mL). Slowly, BCl3 (34 mmol, 2.5 mL, 34 equiv) was added in an ice bath under an inert atmosphere of Ar. The mixture was stirred at room temperature for 1 h. After this period, the reaction was diluted with methanol and filtered through a flash silica pad, yielding the pure product as a light yellow solid (199 mg, 64% yield).

##### Biological Methods: Reagents

Cell culture reagents were purchased from Gibco/Invitrogen (Grand Island, NY, USA). Mycobacteria‐specific Middlebrook 7H9 and 7H10 media were obtained from Difco (Detroit, MI, USA), and OADC and ADC supplements were from BD Biosciences (BD, Sparks, MD, USA). Rifampicin (cod. R7382) and 3‐(4,5‐dimethylthiazol‐2‐yl)‐2,5‐diphenyltetrazolium bromide (MTT) were from Sigma‐Aldrich Co. (St. Louis, MO, USA). The homoisoflavone derivatives and rifampicin were dissolved in dimethyl sulfoxide (DMSO, Sigma Aldrich) and sterilized by passage through 0.22 μm nylon filters (Corning Inc., Wilkes‐Barre, PA, USA).

##### Biological Methods: Viability Assay

The cell viability of RAW 264.7 macrophages and treated with homoisoflavone derivatives was determined by MTT method described by MOSMANN et al. (1983). From the values obtained, the percentage of cytotoxicity was calculated by formula % cytotoxicity = 100‐((Samples‐C‐)*100/C± ‐ C‐). Untreated cells were used as a negative control (C‐), while cells treated with Triton X‐100 were used as positive control of cytotoxicity.

##### Biological Methods: Mycobacterial Culture and Evaluation of Mtb Growth in Bacterial Culture

Two *Mycobacterium tuberculosis* strains, laboratory Mtb strain H37Rv (ATCC), and highly virulent *M. tuberculosis* strain of Beijing genotype (strain M299) isolated from TB patient in Mozambique) were kindly provided by Dr. Philip Suffys (FIOCRUZ, RJ/Brazil). The strains from a single CFU were suspended in Middlebrook 7H9 medium (Difco, BD) supplemented with 10% albumin‐dextrose‐catalase (ADC, BD BBL) and 0.05% Tween‐80 at 37 °C. After 7 days of cultivation, bacterial suspensions were sonicated, vortexed, and kept for 10 min for sedimentation of eventual clumps. Optical densities (ODs) of the suspensions were measured at 600 nm by spectrophotometry and after adjusting to OD_600_ of 0.1. The bacterial suspensions (1 × 10^6^ CFU/well in 96‐well plate) were incubated with or without thiourea (0.8, 4, 20, and 100 μM) or rifampicin (Sigma Aldrich) (ranging from 0.0032 to 1 μg mL^−1^ for Mtb H37Rv strain and from 0.008 to 10 μg/mL for clinical Mtb M299). After incubation for 5 days at 37 °C, 10 μL of MTT solution (5 mg mL^−1^, Sigma Aldrich) were added to each well for 3 h followed by addition of 100 μL lysis buffer (20% w/v SDS, 50% dimethylformamide (DMF) in distilled water, pH 4.7) for 18 h. The ODs were measured at 570 nm. Untreated bacterial suspensions were used to control the spontaneous growth of bacteria.

##### Biological Methods: Infection of Macrophage Cultures and Quantification of Intracellular Growth

RAW 264.7 macrophages (5 × 10^5^ cells mL^−1^) were plated with DMEM‐F12 medium supplemented with 2% FBS for 24 h and incubated at 37 °C and 5% CO_2_. After this period, the macrophage monolayers were infected with Mtb H37Rv at MOI 1:1 (bacteria per macrophage) for 3 h, and then, the extracellular nonphagocytosed bacteria were removed by washing with PBS. Posteriorly, the homoisoflavone derivatives or rifampicin (0.8, 4, 20, and 100 μM) were added and plates incubated at 37 °C for 4 days. On day 4 post infection (p.i.), cells were lysed with 1% saponin solution to release intracellular bacteria, and culture lysates were collected vortexed to obtain single‐cell suspension. Lysate aliquots were serially diluted in PBS and seeded on Middlebrook 7H10 agar. After 21 days at 37 °C, CFU number was quantified, and intracellular growth was expressed in log10. The mean CFU of day 0 was subtracted from mean CFU obtained on day 4 for all conditions (untreated or treated).

##### Biological Methods: SAR Evaluation

The 3D structures of the homoisoflavones derivatives were constructed and optimized using the Spartan’10 software (Wavefunction Inc. Irvine, CA, USA). Initially, a conformational analysis of the compounds was performed using molecular mechanics with MMFF force field, to obtain the conformations of minimum energy. Afterward, the lowest‐energy conformations of the compounds were optimized using the PM3 semiempirical method. Some calculated descriptors included molecular surface area (MSA; Å^2^), highest occupied molecular orbital (HOMO), and lowest unoccupied molecular orbital (LUMO) energy (E_HOMO_ and E_LUMO_; eV), molecular dipole moment (μ; Debye), molecular weight (MW; Da), and molecular volume (MV; Å^3^). Other descriptors such as the logarithm of the octanol/water partition coefficient (LogP), topologic PSA (TPSA), number of hydrogen bond acceptors (HBA), number of hydrogen bond donors (HBD), and the number of rotatable bonds (nRots) were calculated using Molinspiration server.^[^ ^57^ ^]^

##### Biological Methods: In Silico ADMET Studies

The 2D structures of the most active compounds (homoisoflavones derivatives 10, 15, 17, 18, 19, 21, 22, 34, 41, and 42) were submitted to ADMET analysis using admetSAR 3.0.^[^ ^51^ ^]^ The following parameters were predicted: HIA, permeability in Caco‐2 cells, cardiotoxicity (hERG channel inhibitor), hepatotoxicity, and mutagenicity (based on Ames’ test). Also, Lipinski's “Rule of 5” was analyzed to evaluate the theoretical solubility and permeability of orally administered compounds. This parameter states that a drug with good oral bioavailability must have, at least, three of the following requirements: MW ≤ 500 Daltons; LogP ≤ 5; HBA ≤ 10, and HBD ≤ 5.^[^ ^53^ ^]^ Rifampicin was used as a reference in these studies.

## Conflict of Interest

The authors declare no conflict of interest.

## Supporting information

Supplementary Material

## Data Availability

The data that support the findings of this study are available in the supplementary material of this article.
