# Supplementary material for: Intracellular and Extracellular Efficacy of Homoisoflavone Derivatives Against Mycobacterium Tuberculosis: Progress Toward Novel Antitubercular Agents
Source: ChemMedChem. 2025 Jun 4;20(14):e202500249. doi: 10.1002/cmdc.202500249 (PMC12276032; doi:10.1002/cmdc.202500249)

Intracellular and Extracellular Efficacy of Homoisoflavone Derivatives Against *Mycobacterium tuberculosis*: Progress Toward Novel Antitubercular Agents

Sanderson D. Calixto,[a,b‡] *Juliane S. Falcão,*[c‡] *Stella S. Antunes,*[d] *Marlon H. Araujo,*[a] *Alexandre L. B. Cunha,*[c] *David R. Martins,*[c] *Sarah M. R. Nascimento,*[c] *Thatiana L. B. V. Simão,*[b] *Elena B. Lasunskaia,*[b] *Nelilma C. Romeiro,*[d] *Paulo R. R. Costa,*[c] *Michelle F. Muzitano,*[a*] *Guilherme S. Caleffi* [c*]

[a]Laboratório de Produtos Bioativos, Instituto de Ciências Farmacêuticas, Universidade Federal do Rio de Janeiro, 27930-560 Macaé, RJ, Brazil.

[b]Laboratório de Biologia do Reconhecer, Centro de Biociências e Biotecnologia, Universidade Estadual do Norte Fluminense Darcy Ribeiro, 28013-602 Campos dos Goytacazes, RJ, Brazil.

[c]Laboratório de Química Bioorgânica, Instituto de Pesquisas de Produtos Naturais Walter Mors, Universidade Federal do Rio de Janeiro, 21941-902 Rio de Janeiro, RJ, Brazil

[d]Laboratório Integrado de Computação Científica, Instituto Multidisciplinar de Química, Universidade Federal do Rio de Janeiro, 27930-560 Macaé, RJ, Brazil

[‡] Equal contribution as first author

*Correspondence authors: Michelle F. Muzitano, e-mail address: mfmuzitano@macae.ufrj.br; and Guilherme S. Caleffi, e-mail address: guilherme.caleffi@ippn.ufrj.br.

TABLE OF CONTENTS

1. CALCULATED STEREOELECTRONIC DESCRIPTORS 3

2. CHARACTERIZATION DATA OF THE SYHTHESIZED COMPOUNDS 5

2.1 Intermediates 5

2.2 Homoisoflavone derivatives (**1-42**) 6

3. REFERENCES 18

4. NMR SPECTRA OF THE HOMOISOFLAVONE DERIVATIVES **1-42** 22

1. CALCULATED STEREOELECTRONIC DESCRIPTORS

Table S1. Stereoelectronic descriptors calculated for the homoisoflavone derivatives (**1-42**)[a]

Comp.	MW	MSA	MV	PSA	E_{HOMO}	E_{LUMO}	GAP	Dipole	LogP	HBA	HBD	nRot
**1**	270.71	296.81	287.6	26.30	-9.41	-0.75	8.66	1.44	4.30	2	0	1
**2**	270.71	296.88	287.62	26.30	-9.42	-0.74	8.68	1.56	4.27	2	0	1
**3**	305.16	312.54	304.82	26.30	-9.38	-0.82	8.56	1.26	4.90	2	0	1
**4**	272.73	303.20	236.55	26.30	-9.39	-0.54	8.85	1.50	4.18	2	0	2
**5**	254.26	286.22	276.49	26.30	-9.45	-0.79	8.66	1.36	3.78	2	0	1
**6**	254.26	286.26	270.50	26.30	-9.44	-0.78	8.66	1.53	3.76	2	0	1
**7**	304.27	320.23	309.87	26.30	-9.54	-1.00	8.54	2.44	4.51	2	0	2
**8**	304.27	320.18	309.86	26.30	-9.50	-0.91	8.59	2.33	4.49	2	0	2
**9**	315.17	302.22	293.80	26.30	-9.45	-0.78	8.67	1.38	4.43	2	0	1
**10**	266.30	311.07	300.97	35.54	-9.18	-0.64	8.54	2.20	3.68	3	0	2
**11**	252.27	289.93	280.27	46.53	-9.24	-0.66	8.58	2.50	3.14	3	1	1
**12**	252.27	290.05	280.32	46.53	-9.36	-0.71	8.65	1.56	3.12	3	1	1
**13**	378.37	408.28	402.32	35.54	-9.28	-0.66	8.62	2.33	5.53	3	0	4
**14**	378.37	411.24	402.55	35.54	-9.34	-0.69	8.65	1.34	5.50	3	0	4
**15**	280.28	307.43	300.09	44.77	-9.17	-0.71	8.46	1.80	3.51	4	0	1
**16**	282.29	321.26	311.34	55.77	-8.92	-0.68	8.24	1.29	2.96	4	1	2
**17**	282.29	319.88	310.83	55.77	-9.06	-0.70	8.36	2.91	2.96	4	1	2
**18**	286.71	305.69	297.53	46.53	-9.28	-0.73	8.55	1.79	4.01	3	1	1
**19**	331.16	310.89	303.66	46.53	-9.37	-0.75	8.62	1.77	4.14	3	1	1
**20**	345.19	332.42	324.27	35.54	-9.29	-0.71	8.58	2.15	4.41	3	0	2

**21**	302.33	340.50	336.30	44.13	-9.24	-0.99	8.25	2.89	2.95	4	0	2
**22**	271.23	292.82	280.61	85.27	-9.73	-1.63	8.10	6.36	2.78	6	0	2
**23**	287.29	301.60	291.29	72.13	-9.70	-1.93	7.77	5.57	3.42	5	0	2
**24**	347.33	371.51	366.93	85.27	-9.22	-1.75	7.47	6.47	4.58	6	0	3
**25**	305.16	314.98	305.57	26.30	-9.20	-0.88	8.32	2.02	4.95	2	0	1
**26**	300.74	329.24	318.94	35.54	-9.13	-0.77	8.36	3.20	4.33	3	0	2
**27**	288.77	304.33	294.43	26.30	-9.47	-0.95	8.52	2.66	4.44	2	0	1
**28**	284.29	318.59	307.81	35.54	-9.25	-0.85	8.40	3.92	3.82	3	0	2
**29**	349.61	320.36	311.73	26.30	-9.48	-0.91	8.57	2.18	5.08	2	0	1
**30**	345.19	334.61	325.10	35.54	-9.24	-0.81	8.43	3.40	4.46	3	0	2
**31**	296.32	343.53	332.28	44.77	-9.15	-0.57	8.58	3.12	3.71	4	0	3
**32**	326.35	374.59	363.24	54.01	-8.86	-0.59	8.27	2.64	3.30	5	0	4
**33**	282.29	322.27	311.55	55.77	-9.17	-0.60	8.57	1.34	3.17	4	1	2
**34**	312.32	353.32	342.50	65.00	-8.85	-0.62	8.23	0.68	2.76	5	1	3
**35**	326.35	373.36	362.76	54.01	-9.10	-0.41	8.69	2.00	3.69	5	0	4
**36**	356.37	404.1	394.87	63.24	-9.07	-0.56	8.51	3.24	3.48	6	0	5
**37**	312.32	350.37	341.98	65.00	-9.26	-0.56	8.70	3.23	3.40	5	1	3
**38**	298.29	328.09	320.92	76.00	-9.27	-0.62	8.65	3.00	3.33	5	2	2
**39**	342.35	380.13	373.67	74.23	-9.21	-0.70	8.51	2.48	3.19	6	1	4
**40**	353.85	393.57	388.69	29.54	-8.62	-0.62	8.00	2.87	5.28	3	0	2
**41**	354.36	389.57	381.68	88.51	-8.82	-1.53	7.29	8.18	3.77	7	0	3
**42**	370.43	398.35	392.36	75.37	-8.80	-1.85	6.95	7.28	4.41	6	0	3

[a]Molecular weight (MW; Da), molecular surface area (MSA; Å^2), molecular volume (MV; Å^3), topologic polar surface area (PSA; Å^2), energy of the highest occupied molecular orbital and the lowest unoccupied molecular orbital (E_{HOMO} and E_{LUMO}; eV), HOMO-LUMO energy gap (GAP); dipole moment (Debye), logarithm of the octanol/water partition coefficient (LogP), number of hydrogen bond acceptors and donors (HBA and HBD) and number of rotatable bonds (nRot).

2. CHARACTERIZATION DATA OF THE SYHTHESIZED COMPOUNDS

2.1 Intermediates

*1-(2-hydroxy-4,6-dimethoxyphenyl)ethan-1-one (**50**):* The compound was obtained as a white solid (1.746 g, 89% yield). **^{1}H NMR** (500 MHz, $CDCl_3$) δ 14.02 (s, 1H), 6.06 (d, *J* = 2.4 Hz, 1H), 5.92 (d, *J* = 2.4 Hz, 1H), 3.86 (s, 3H), 3.82 (s, 3H), 2.61 (s, 3H). **^{13}C NMR** (101 MHz, $CDCl_3$) δ 203.3, 167.7, 166.2, 163.0, 106.1, 93.6, 90.8, 55.6, 33.0. The spectroscopic data were consistent with those reported in the literature.[1]

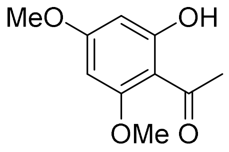

*1-(6-hydroxy-2,3,4-trimethoxyphenyl)ethan-1-one (**53**):* The compound was obtained as a white solid (2.217g, 98% yield). **^{1}H NMR** (400 MHz, $CDCl_3$) δ 13.38 (s, 1H), 6.18 (s, 1H), 3.94 (s, 3H), 3.83 (s, 3H), 3.73 (s, 3H), 2.60 (s, 3H). **^{13}C NMR** (101 MHz, $CDCl_3$) δ 203.3, 161.9, 160.1, 155.2, 134.7, 108.4, 96.1, 61.0, 61.0, 56.1, 31.9. The spectroscopic data were consistent with those reported in the literature.[1]

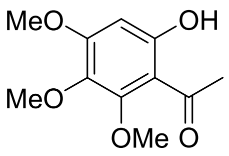

*5,7-dimethoxy-4H-chromen-4-one (**51**):* The compound was obtained as an orange solid (1.010 g, 98% yield). **^{1}H NMR** (500 MHz, $CDCl_3$) δ 7.61 (d, *J* = 5.9 Hz, 1H), 6.44 (d, *J* = 2.3 Hz, 1H), 6.36 (d, *J* = 2.3 Hz, 1H), 6.19 (d, *J* = 5.9 Hz, 1H), 3.94 (s, 3H), 3.88 (s, 3H). **^{13}C NMR** (101 MHz $CDCl_3$) δ 176.7, 164.0, 161.1, 160.2, 152.7, 114.6, 110.4, 96.2, 92.9, 56.5, 55.8. The spectroscopic data were consistent with those reported in the literature.[1]

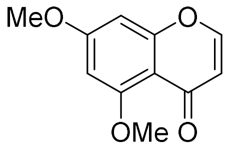

*5,6,7-trimethoxy-4H-chromen-4-one (**54**):* The compound was obtained as a brown solid (1.157 g, 98% yield). **^{1}H NMR** (500 MHz, $CDCl_3$) δ 7.66 (d, *J* = 5.9 Hz, 1H), 6.68 (s, 1H), 6.18 (d, *J* = 6.0 Hz, 1H), 3.96 (s, 3H), 3.95 (s, 3H), 3.90 (s, 3H). **^{13}C NMR** (126 MHz, $CDCl_3$) δ 176.4, 157.9, 154.9, 153.1, 152.6, 140.5, 114.0, 113.9, 96.3, 62.2, 61.6, 56.3. The spectroscopic data were consistent with those reported in the literature.[1]

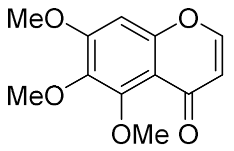

*7-Hydroxy-chromanon-4-one (**43e**):* The compound was obtained as a brown solid (705 mg, 54% yield). **^{1}H NMR** (400 MHz, $CDCl_3$) δ 7.83 (d, *J* = 8.7 Hz, 1H), 6.52 (dd, *J* = 8.7, 2.3 Hz, 1H), 6.40 (d, *J* = 2.3 Hz, 1H), 6.03 (s, 1H), 4.51 (t, *J* = 6.4 Hz, 2H), 2.82 – 2.71 (m, 2H). The spectroscopic data were consistent with those reported in the literature.[2]

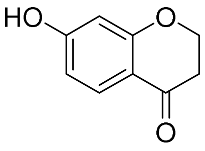

*4-oxochroman-7-yl trifluoromethanesulfonate (**48**):* The compound was purified by flash chromatography (*n*-Hexane/EtOAc 80:20), yielding the pure product as a white solid (613 mg, 83% yield). **¹H NMR** (500 MHz, $CDCl_3$) δ 8.00 (d, *J* = 9.4 Hz, 1H), 6.96 – 6.92 (m, 2H), 4.62 – 4.59 (m, 2H), 2.89 – 2.83 (m, 2H). **¹³C NMR** (126 MHz, $CDCl_3$) δ 190.0, 162.7, 153.9, 129.5, 121.1, 118.6 (q, *J* = 320.8 Hz), 114.5, 111.1, 67.6, 37.3. The spectroscopic data were consistent with those reported in the literature.[3]

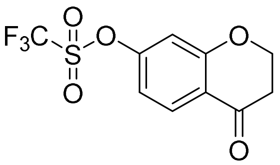

*7-(piperidin-1-yl)chroman-4-one (**43f**):* The compound was purified by flash chromatography (*n*-Hexane/EtOAc 80:20), yielding the pure product as a white solid (477 mg, 98 % yield). **¹H NMR** (400 MHz, $CDCl_3$) δ 7.75 (d, *J* = 9.0 Hz, 1H), 6.53 (dd, *J* = 9.0, 2.4 Hz, 1H), 6.24 (d, *J* = 2.4 Hz, 1H), 4.50 – 4.43 (m, 2H), 3.37 (d, *J* = 5.2 Hz, 4H), 2.74 – 2.67 (m, 2H), 1.65 (s, 6H).

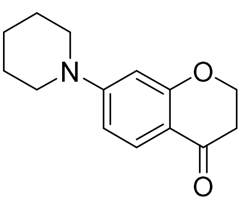

*5,7-dimethoxychroman-4-one (**43g**):* The compound was obtained as a white solid (674 mg, 81% yield). **¹H NMR** (500 MHz, $CDCl_3$) δ 6.06 (s, 2H), 4.44 (dd, *J* = 6.8, 6.0 Hz, 2H), 3.88 (s, 3H), 3.82 (s, 3H), 2.75 – 2.69 (m, 2H). The spectroscopic data were consistent with those reported in the literature.[1]

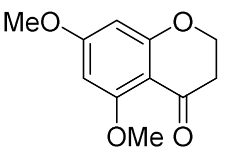

*5,6,7-trimethoxychroman-4-one (**43h**):* The compound was obtained as a white solid (857 mg, 90% yield). **¹H NMR** (500 MHz, $CDCl_3$) δ 6.19 (s, 1H), 4.42 – 4.32 (m, 2H), 3.85 (s, 3H), 3.82 (s, 3H), 3.74 (s, 3H), 2.64 (dd, J = 6.9, 6.0 Hz, 2H). The spectroscopic data were consistent with those reported in the literature.[1]

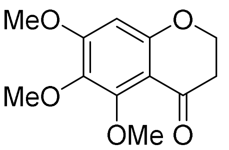

*4-(1H-imidazol-1-yl)benzaldehyde (**44b**):* The compound was purified by flash chromatography (*n*-Hexane/EtOAc 20:80), yielding the pure product as a light yellow solid (96 mg, 28% yield). **¹H NMR** (500 MHz, $CDCl_3$) δ 10.05 (s, 1H), 8.03 (d, *J* = 8.6 Hz, 2H), 7.99 (s, 1H), 7.59 (d, *J* = 8.5 Hz, 2H), 7.38 (s, 1H), 7.27 (s, 1H). The spectroscopic data were consistent with those reported in the literature.[4]

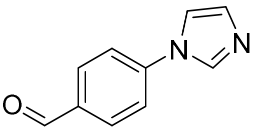

2.2 Homoisoflavone derivatives (1-42)

*(E)-3-(4-chlorobenzylidene)chroman-4-one (**1**):* The compound was purified by recrystallization from hot methanol as a light yellow solid (154 mg, 57% yield). The purity (98%) was determined by HPLC analysis using a Waters C18 column (250 x 4.6 mm, 5 µm), CH_3CN/H_2O 80:20, 1 mL/min, 28 ºC, t_R: 6.7 min, 254 nm. **¹H NMR** (400 MHz, $CDCl_3$) δ 8.0 (dd, *J* = 7.9, 1.7 Hz, 1H), 7.8 (s, 1H), 7.5 (ddd, *J* = 8.3, 7.2, 1.8 Hz, 1H), 7.4 (d, *J* = 8.5 Hz, 2H), 7.3 (d, *J* = 8.3 Hz, 2H), 7.1 (ddd, *J* = 8.1, 7.2, 1.1 Hz, 1H), 7.0 (dd, *J* = 8.4, 1.1 Hz, 1H), 5.3 (d, *J* = 1.9 Hz, 2H). **¹³C{H} NMR** (101

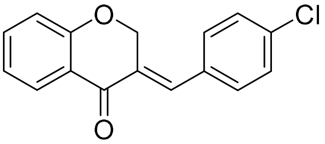

MHz, $CDCl_3$) δ 182.0, 161.1, 136.1, 136.0, 135.6, 132.8, 131.4, 131.2, 129.1, 128.0, 122.0, 121.9, 117.9, 67.4. The spectroscopic data were consistent with those reported in the literature.[5]

*(E)-3-(3-chlorobenzylidene)chroman-4-one (**2**):* The compound was purified by recrystallization

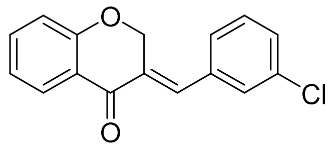

from hot methanol as white solid (254 mg, 94% yield). The purity (99%) was determined by HPLC analysis using a Waters C18 column (250 x 4.6 mm, 5 µm), CH_3CN/H_2O 80:20, 1 mL/min, 28 ºC, t_R: 6.7 min, 254 nm. **^{1}H NMR** (500 MHz, $CDCl_3$) δ 8.02 (d, *J* = 7.9 Hz, 1H), 7.79 (s, 1H), 7.53 – 7.48 (m, 1H), 7.39 (d, *J* = 4.8 Hz, 2H), 7.29 (s, 1H), 7.19 (t, *J* = 4.6 Hz, 1H), 7.08 (t, *J* = 7.5 Hz, 1H), 6.98 (d, *J* = 8.3 Hz, 1H), 5.31 (s, 2H). **^{13}C{H} NMR** (126 MHz, $CDCl_3$) δ 182.1, 161.3, 136.2, 136.2, 135.8, 134.9, 132.2, 130.1, 129.8, 129.5, 128.1, 128.1, 122.2, 122.0, 118.1, 67.5. The spectroscopic data were consistent with those reported in the literature.[6]

*(E)-3-(3,4-dichlorobenzylidene)chroman-4-one (**3**):* The compound was purified by

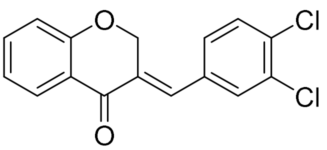

recrystallization from hot methanol as white solid (302 mg, 99% yield). The purity (99%) was determined by HPLC analysis using a Waters C18 column (250 x 4.6 mm, 5 µm), CH_3CN/H_2O 80:20, 1 mL/min, 28 ºC, t_R: 8.1 min, 254 nm. **^{1}H NMR** (400 MHz, $CDCl_3$) δ 8.02 (dd, *J* = 7.9, 1.7 Hz, 1H), 7.74 (s, 1H), 7.56 – 7.48 (m, 2H), 7.40 (d, *J* = 2.0 Hz, 1H), 7.14 (dd, *J* = 8.3, 2.1 Hz, 1H), 7.09 (ddd, *J* = 8.1, 7.2, 1.1 Hz, 1H), 6.98 (dd, *J* = 8.4, 1.1 Hz, 1H), 5.29 (d, *J* = 1.9 Hz, 2H). **^{13}C{H} NMR** (101 MHz, $CDCl_3$) δ 182.3, 161.1, 160.8, 137.4, 135.8, 132.2, 129.0, 128.0, 127.1, 122.2, 121.9, 117.9, 114.4, 67.9, 55.5. The spectroscopic data were consistent with those reported in the literature.[7]

*3-(4-chlorobenzyl)chroman-4-one (**4**):* The compound was purified by flash chromatography (n-

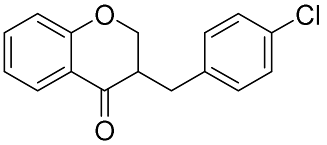

Hexane/EtOAc 80:20), yielding the pure product as a white solid (90 mg, 69 % yield). **^{1}H NMR** (400 MHz, $CDCl_3$) δ 7.92 (dd, *J* = 7.9, 1.8 Hz, 1H), 7.48 (ddd, *J* = 8.6, 7.2, 1.8 Hz, 1H), 7.29 (d, *J* = 8.4 Hz, 2H), 7.17 (d, *J* = 8.4 Hz, 2H), 7.04 (ddd, *J* = 8.1, 7.2, 1.1 Hz, 1H), 6.97 (dd, *J* = 8.4, 1.1 Hz, 1H), 4.37 (dd, *J* = 11.6, 4.3 Hz, 1H), 4.16 (dd, *J* = 11.5, 8.4 Hz, 1H), 3.23 (dd, *J* = 14.0, 4.7 Hz, 1H), 2.90 (ddt, *J* = 10.0, 8.8, 4.5 Hz, 1H), 2.72 (dd, *J* = 14.0, 10.0 Hz, 1H). **^{13}C{H} NMR** (101 MHz, $CDCl_3$) δ 193.4, 161.5, 136.7, 136.0, 132.5, 130.4, 128.8, 127.5, 121.6, 120.5, 117.8, 69.3, 47.5, 31.8. The spectroscopic data were partially consistent with those reported in the literature.[8]

*(E)-3-(4-fluorobenzylidene)chroman-4-one (**5**):* The compound was purified by recrystallization from hot methanol as white solid (119 mg, 47% yield). The purity (97%) was determined by

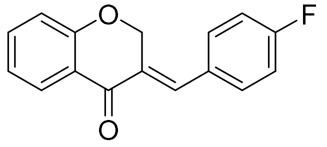

HPLC analysis using a Waters C18 column (250 x 4.6 mm, 5 µm), CH_3CN/H_2O 80:20, 1 mL/min, 28 ºC, t_R: 5.4 min, 254 nm. **^{1}H NMR** (400 MHz, $CDCl_3$) δ 8.02 (dd, *J* = 7.9, 1.6 Hz, 1H), 7.83 (s,

1H), 7.49 (ddd, J = 8.3, 7.2, 1.8 Hz, 1H), 7.30 (dd, J = 8.5, 5.4 Hz, 2H), 7.15 (t, J = 8.6 Hz, 2H), 7.08 (ddd, J = 8.0, 7.2, 1.1 Hz, 1H), 6.97 (dd, J = 8.3, 1.1 Hz, 1H), 5.32 (d, J = 1.9 Hz, 2H). **^{13}C{H} NMR** (101 MHz, $CDCl_3$) δ 182.2, 163.3 (d, $^{1}J_{C-F}$ = 251.4 Hz), 161.2, 136.4, 136.1, 132.2, 130.9 (d, $^{4}J_{C-F}$ = 1.5 Hz), 130.7 (d, $^{3}J_{C-F}$ = 3.4 Hz), 128.1, 122.1, 122.1, 118.1, 116.1 (d, $^{2}J_{C-F}$ = 21.8 Hz), 67.6. The spectroscopic data were consistent with those reported in the literature. [6]

*(E)-3-(3-fluorobenzylidene)chroman-4-one (**6**):* The compound was purified by recrystallization

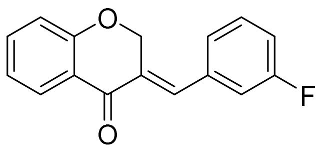

from hot methanol as white solid (76 mg, 30% yield). The purity (99%) was determined by HPLC analysis using a Waters C18 column (250 x 4.6 mm, 5 µm), CH_3CN/H_2O 80:20, 1 mL/min, 28 ºC, t_R: 5.5 min, 254 nm. **^{1}H NMR** (400 MHz, $CDCl_3$) δ 8.02 (dd, J = 7.9, 1.8 Hz, 1H), 7.81 (s, 1H), 7.50 (ddd, J = 8.6, 7.1, 1.8 Hz, 1H), 7.42 (td, J = 8.0, 5.9 Hz, 1H), 7.15 – 7.06 (m, 3H), 7.03 – 6.96 (m, 2H), 5.32 (d, J = 1.9 Hz, 2H). **^{13}C{H} NMR** (101 MHz, $CDCl_3$) δ 182.1, 168.8 (d, $^{1}J_{C-F}$ = 247.6 Hz), 161.3, 136.6 (d, $^{3}J_{C-F}$ = 7.8 Hz), 136.2, 136.0, 132.1, 130.5 (d, $^{3}J_{C-F}$ = 8.4 Hz), 128.1, 125.8 (d, $^{4}J_{C-F}$ = 3.0 Hz), 122.2, 122.0, 118.1, 116.7 (d, $^{2}J_{C-F}$ = 15.7 Hz), 116.5 (d, $^{2}J_{C-F}$ = 15.0 Hz), 67.6. The spectroscopic data were consistent with those reported in the literature.[9]

*(E)-3-(4-(trifluoromethyl)benzylidene)chroman-4-one (**7**):* The compound was purified by

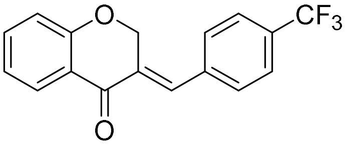

recrystallization from hot methanol as white solid (149 mg, 49% yield). The purity (98%) was determined by HPLC analysis using a Waters C18 column (250 x 4.6 mm, 5 µm), CH_3CN/H_2O 80:20, 1 mL/min, 28 ºC, t_R: 5.2 min, 254 nm. **^{1}H NMR** (400 MHz, $CDCl_3$) δ 8.04 (dd, J = 7.9, 1.7 Hz, 1H), 7.87 (s, 1H), 7.72 (d, J = 8.1 Hz, 2H), 7.52 (ddd, J = 8.3, 7.2, 1.8 Hz, 1H), 7.43 (d, J = 8.3 Hz, 2H), 7.10 (ddd, J = 8.1, 7.2, 1.1 Hz, 1H), 6.99 (dd, J = 8.4, 1.0 Hz, 1H), 5.31 (d, J = 1.9 Hz, 2H). **^{13}C{H} NMR** (126 MHz, $CDCl_3$) δ 182.0, 161.3, 138.0, 136.3, 135.6, 132.9, 131.2 (q, $^{2}J_{C-F}$ = 32.8 Hz), 130.1, 128.1, 125.8 (q, $^{3}J_{C-F}$ = 3.8 Hz), 123.9 (q, $^{1}J_{C-F}$ = 272.4 Hz), 122.3, 121.9, 118.1, 67.4. The spectroscopic data were consistent with those reported in the literature.[10]

*(E)-3-(3-(trifluoromethyl)benzylidene)chroman-4-one (**8**):* The compound was purified by

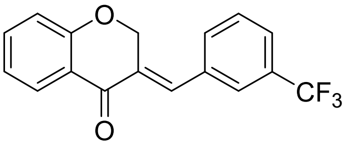

recrystallization from hot methanol as white solid (55 mg, 18% yield). The purity (98%) was determined by HPLC analysis using a Waters C18 column (250 x 4.6 mm, 5 µm), CH_3CN/H_2O 80:20, 1 mL/min, 28 ºC, t_R: 6.1 min, 254 nm. **^{1}H NMR** (400 MHz, $CDCl_3$) δ 8.04 (dd, J = 7.9, 1.7 Hz, 1H), 7.87 (s, 1H), 7.68 (d, J = 7.8 Hz, 1H), 7.60 (t, J = 7.8 Hz, 1H), 7.57 – 7.47 (m, 3H), 7.10 (ddd, J = 8.0, 7.2, 1.1 Hz, 1H), 6.99 (dd, J = 8.4, 1.1 Hz, 1H), 5.32 (d, J = 1.9 Hz, 2H). **^{13}C{H} NMR** (101 MHz, $CDCl_3$) δ 181.9, 161.0, 156.8, 135.9, 135.6, 134.9, 130.9, 130.1, 128.2, 127.9, 122.0, 117.9, 112.1, 111.8, 67.5, 56.4. HRMS: calculated $[M + H]^+$ = 305.0784, experimental $[M + H]^+$ = 305.0792, error: 2.62 ppm.

(E)-3-(4-bromobenzylidene)chroman-4-one (**9**): The compound was purified by recrystallization

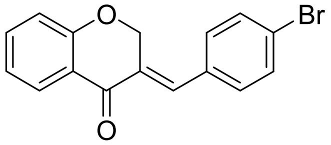

from hot methanol as white solid (189 mg, 60% yield). The purity (99%) was determined by HPLC analysis using a Waters C18 column (250 x 4.6 mm, 5 μm), CH_3CN/H_2O 80:20, 1 mL/min, 28 ºC, t_R: 7.1 min, 254 nm. **^{1}H NMR** (400 MHz, $CDCl_3$) δ 8.02 (dd, *J* = 8.0, 1.6 Hz, 1H), 7.79 (s, 1H), 7.59 (d, *J* = 8.4 Hz, 2H), 7.51 (ddd, *J* = 8.4, 7.2, 1.8 Hz, 1H), 7.18 (d, *J* = 8.3 Hz, 2H), 7.09 (ddd, *J* = 8.0, 7.2, 1.1 Hz, 1H), 6.98 (dd, *J* = 8.5, 1.1 Hz, 1H), 5.31 (d, *J* = 1.9 Hz, 2H). **^{13}C{H} NMR** (101 MHz, $CDCl_3$) δ 182.0, 161.1, 136.1, 136.0, 133.2, 132.0, 131.5, 131.4, 128.0, 123.9, 122.0, 121.9, 117.9, 67.4. The spectroscopic data were consistent with those reported in the literature.[9]

(E)-3-(4-methoxybenzylidene)chroman-4-one (***10***): The compound was purified by

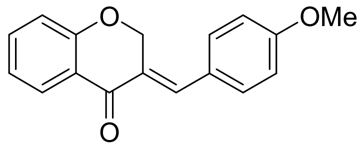

recrystallization from hot methanol as yellow solid (152 mg, 57% yield). The purity (99%) was determined by HPLC analysis using a Waters C18 column (250 x 4.6 mm, 5 μm), CH_3CN/H_2O 80:20, 1 mL/min, 28 ºC, t_R: 5.4 min, 254 nm. **^{1}H NMR** (500 MHz, $CDCl_3$) δ 8.02 (dd, *J* = 7.8, 1.8 Hz, 1H), 7.83 (s, 1H), 7.47 (ddd, *J* = 8.7, 7.2, 1.8 Hz, 1H), 7.27 (d, *J* = 8.7 Hz, 2H), 7.06 (t, *J* = 7.9 Hz, 1H), 6.98 – 6.93 (m, 3H), 5.37 (d, *J* = 1.9 Hz, 2H), 3.85 (s, 3H). **^{13}C{H} NMR** (126 MHz, $CDCl_3$) δ 182.3, 161.1, 160.8, 137.4, 135.8, 132.2, 129.0, 128.0, 127.1, 122.2, 121.9, 117.9, 114.4, 67.9, 55.5. The spectroscopic data were consistent with those reported in the literature.[6]

(E)-3-(4-hydroxybenzylidene)chroman-4-one (***11***): The compound was purified by

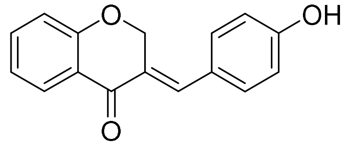

recrystallization from hot methanol as yellow solid (237 mg, 94% yield). The purity (99%) was determined by HPLC analysis using a Waters C18 column (250 x 4.6 mm, 5 μm), CH_3CN/H_2O 80:20, 1 mL/min, 28 ºC, t_R: 4.3 min, 254 nm. **^{1}H NMR** (400 MHz, DMSO-*d*$_6$) δ 7.86 (dd, *J* = 7.8, 1.7 Hz, 1H), 7.68 (s, 1H), 7.58 (ddd, *J* = 8.6, 7.2, 1.8 Hz, 1H), 7.34 (d, *J* = 8.6 Hz, 2H), 7.12 (ddd, *J* = 8.1, 7.3, 1.1 Hz, 1H), 7.04 (dd, *J* = 8.4, 1.0 Hz, 1H), 6.88 (d, *J* = 8.6 Hz, 2H), 5.42 (d, *J* = 1.9 Hz, 2H). **^{13}C{H} NMR** (101 MHz, DMSO-*d*$_6$) δ 181.1, 160.5, 159.4, 137.0, 136.0, 132.9, 127.6, 127.2, 124.8, 121.9, 121.7, 117.9, 115.9, 67.6. The spectroscopic data were consistent with those reported in the literature.[11]

(E)-3-(3-hydroxybenzylidene)chroman-4-one (***12***): The compound was purified by

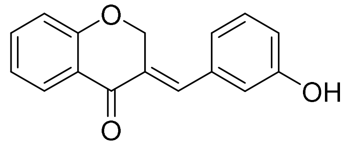

recrystallization from hot methanol as white solid (123 mg, 49% yield). The purity (97%) was determined by HPLC analysis using a Waters C18 column (250 x 4.6 mm, 5 μm), CH_3CN/H_2O 80:20, 1 mL/min, 28 ºC, t_R: 4.3 min, 254 nm. **^{1}H NMR** (400 MHz, DMSO-*d*$_6$) δ 7.88 (dd, *J* = 7.8, 1.8 Hz, 1H), 7.67 (s, 1H), 7.59 (ddd, *J* = 8.8, 7.2, 1.8 Hz, 1H), 7.30

(t, J = 7.8 Hz, 1H), 7.13 (ddd, J = 8.1, 7.2, 1.1 Hz, 1H), 7.05 (dd, J = 8.4, 1.0 Hz, 1H), 6.91 – 6.85 (m, 2H), 6.83 (s, 1H), 5.40 (d, J = 1.9 Hz, 2H). **^{13}C{H} NMR** (101 MHz, DMSO-d_6) δ 181.2, 160.7, 157.5, 136.8, 136.2, 135.0, 130.6, 129.9, 127.3, 122.0, 121.5, 121.1, 117.9, 116.9, 116.7, 67.4. The spectroscopic data were consistent with those reported in the literature.[11]

*(E)-3-(4-((2,4-difluorobenzyl)oxy)benzylidene)chroman-4-one (**13**):* The compound was purified by flash chromatography (n-Hexane/EtOAc 80:20) (132 mg, 35% yield). The purity (99%) was determined by HPLC analysis using a Waters C18 column (250 x 4.6 mm, 5 µm), CH_3CN/H_2O 80:20, 1 mL/min, 28 ºC, t_R: 7.5 min, 254 nm. **^{1}H NMR** (400 MHz, $CDCl_3$) δ 8.02 (dd, J = 7.9, 1.6 Hz, 1H), 7.83 (s, 1H), 7.48 (td, J = 8.3, 7.8, 4.0 Hz, 2H), 7.29 (d, J = 8.7 Hz, 2H), 7.10 – 7.01 (m, 3H), 7.00 – 6.82 (m, 3H), 5.37 (d, J = 1.7 Hz, 2H), 5.13 (s, 2H). **^{13}C{H} NMR** (101 MHz, $CDCl_3$) δ 182.2, 163.0 (dd, $^1J_{C-F}$ = 232.0 Hz, $^3J_{C-F}$ = 12.0 Hz), 160.1, 160.5 (dd, $^1J_{C-F}$ = 232.4, $^3J_{C-F}$ = 12.0 Hz), 159.4, 137.1, 135.7, 132.1, 130.9 (dd, $^3J_{C-F}$ = 9.8 Hz, $^3J_{C-F}$ = 5.5 Hz), 129.2, 127.9, 127.6, 122.1, 121.9, 119.6 (dd, $^2J_{C-F}$ = 14.5 Hz, $^4J_{C-F}$ = 3.8 Hz), 117.8, 115.0, 111.7 (dd, $^2J_{C-F}$ = 21.3 Hz, $^4J_{C-F}$ = 3.8 Hz), 104.0 (t, $^2J_{C-F}$ = 25.3 Hz), 67.7, 63.3. HRMS: calculated $[M + H]^+$ = 379.1140, experimental $[M + H]^+$ = 379.1138, error: -0.53 ppm.

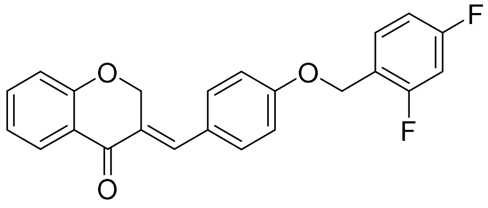

*(E)-3-(3-((2,4-difluorobenzyl)oxy)benzylidene)chroman-4-one (**14**):* The compound was purified by flash chromatography (n-Hexane/EtOAc 80:20) (155 mg, 41% yield). The purity (97%) was determined by HPLC analysis using a Waters C18 column (250 x 4.6 mm, 5 µm), CH_3CN/H_2O 80:20, 1 mL/min, 28 ºC, t_R: 7.7 min, 254 nm. **^{1}H NMR** (500 MHz, $CDCl_3$) δ 8.04 (dd, J = 7.9, 1.7 Hz, 1H), 7.84 (s, 1H), 7.54 – 7.46 (m, 2H), 7.39 (t, J = 7.9 Hz, 1H), 7.09 (ddd, J = 8.1, 7.1, 1.1 Hz, 1H), 7.04 (ddd, J = 8.3, 2.6, 0.9 Hz, 1H), 6.98 (dd, J = 8.4, 1.1 Hz, 1H), 6.96 – 6.86 (m, 4H), 5.33 (d, J = 1.9 Hz, 2H), 5.13 (s, 2H). **^{13}C{H} NMR** (126 MHz, $CDCl_3$) δ δ 182.3, 162.9 (dd, $^1J_{C-F}$ = 275.2 Hz, $^3J_{C-F}$ = 12.0 Hz), 160.1 (dd, $^1J_{C-F}$ = 287.8 Hz, $^3J_{C-F}$ = 11.9 Hz), 161.3, 158.6, 137.3, 136.1, 136.0, 131.4, 130.1 (dd, $^3J_{C-F}$ = 9.8 Hz, $^3J_{C-F}$ = 5.5 Hz), 130.0, 128.1, 123.0, 122.1, 122.1, 119.9 (dd, $^2J_{C-F}$ = 14.6 Hz, $^4J_{C-F}$ = 3.8 Hz), 118.1, 116.4, 116.0, 111.7 (dd, $^2J_{C-F}$ = 21.3 Hz, $^4J_{C-F}$ = 3.8 Hz), 104.1 (t, $^2J_{C-F}$ = 25.3 Hz), 67.7, 63.5. HRMS: calculated $[M + H]^+$ = 379.1140, experimental $[M + H]^+$ = 379.1138, error: -0.53 ppm.

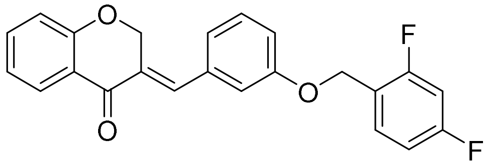

*(E)-3-(benzo[d][1,3]dioxol-5-ylmethylene)chroman-4-one (**15**):* The compound was purified by recrystallization from hot methanol as yellow solid (120 mg, 43% yield). The purity (99%) was determined by HPLC analysis using a Waters C18 column (250 x 4.6 mm, 5 µm), CH_3CN/H_2O 80:20, 1 mL/min, 28 ºC, t_R: 5.0 min, 254 nm. **^{1}H NMR** (500 MHz,

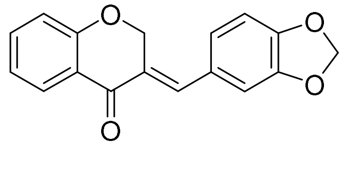

CDCl3) δ 8.01 (dd, *J* = 7.9, 1.5 Hz, 1H), 7.78 (s, 1H), 7.48 (ddd, *J* = 8.5, 7.2, 1.8 Hz, 1H), 7.07 (t, *J* = 7.5 Hz, 1H), 6.96 (d, *J* = 8.3 Hz, 1H), 6.89 (d, *J* = 8.0 Hz, 1H), 6.84 (dd, *J* = 8.0, 1.7 Hz, 1H), 6.79 (d, *J* = 1.7 Hz, 1H), 6.04 (s, 2H), 5.35 (d, *J* = 1.9 Hz, 2H). ^{13}**C{H} NMR** (101 MHz, $CDCl_3$) δ 182.2, 161.1, 149.0, 148.2, 137.5, 135.9, 129.6, 128.6, 128.0, 125.5, 122.2, 122.0, 118.0, 110.0, 108.8, 101.8, 67.9. The spectroscopic data were consistent with those reported in the literature.[12]

*(E)-3-(3-hydroxy-4-methoxybenzylidene)chroman-4-one (**16**):* The compound was purified by

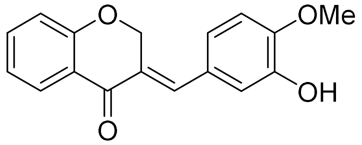

recrystallization from hot methanol as yellow solid (253 mg, 90% yield). The purity (99%) was determined by HPLC analysis using a Waters C18 column (250 x 4.6 mm, 5 µm), CH_3CN/H_2O 80:20, 1 mL/min, 28 ºC, t_R: 4.1 min, 254 nm. ^{1}**H NMR** (400 MHz, DMSO-d_6) δ 9.31 (s, 1H), 7.86 (dd, *J* = 7.9, 1.8 Hz, 1H), 7.62 (s, 1H), 7.57 (ddd, *J* = 8.3, 7.2, 1.8 Hz, 1H), 7.11 (ddd, *J* = 8.1, 7.2, 1.1 Hz, 1H), 7.07 – 7.01 (m, 2H), 6.95 – 6.86 (m, 2H), 5.42 (d, *J* = 1.9 Hz, 2H), 3.83 (s, 3H). ^{13}**C{H} NMR** (126 MHz, $CDCl_3$) δ 182.4, 161.2, 147.9, 145.8, 137.6, 135.9, 129.5, 128.0, 128.0, 123.7, 122.2, 122.0, 118.0, 116.1, 110.7, 67.9, 56.2. The spectroscopic data were consistent with those reported in the literature.[13]

*(E)-3-(4-hydroxy-3-methoxybenzylidene)chroman-4-one (**17**):* The compound was purified by

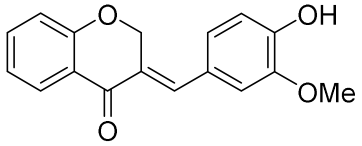

recrystallization from hot methanol as yellow solid (121 mg, 43% yield). The purity (99%) was determined by HPLC analysis using a Waters C18 column (250 x 4.6 mm, 5 µm), CH_3CN/H_2O 80:20, 1 mL/min, 28 ºC, t_R: 4.1 min, 254 nm. ^{1}**H NMR** (400 MHz, $CDCl_3$) δ 8.02 (dd, *J* = 7.9, 1.7 Hz, 1H), 7.81 (s, 1H), 7.48 (ddd, *J* = 8.3, 7.1, 1.8 Hz, 1H), 7.07 (ddd, *J* = 8.0, 7.2, 1.1 Hz, 1H), 6.98 (t, *J* = 9.4 Hz, 2H), 6.87 – 6.83 (m, 2H), 5.89 (s, 1H), 5.39 (d, *J* = 1.9 Hz, 2H), 3.94 (s, 3H). ^{13}**C{H} NMR** (126 MHz, $CDCl_3$) δ 182.3, 161.1, 147.4, 146.7, 137.8, 135.8, 129.1, 128.0, 127.0, 124.3, 122.2, 122.0, 117.9, 114.9, 113.0, 67.9, 56.1. The spectroscopic data were consistent with those reported in the literature.[13]

*(E)-3-(3-chloro-4-hydroxybenzylidene)chroman-4-one (**18**):* The compound was purified by

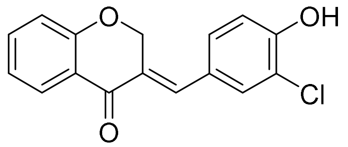

recrystallization from hot methanol as yellow solid (286 mg, 65% yield). The purity (99%) was determined by HPLC analysis using a Waters C18 column (250 x 4.6 mm, 5 µm), CH_3CN/H_2O 80:20, 1 mL/min, 28 ºC, t_R: 4.9 min, 365 nm. ^{1}**H NMR** (400 MHz, $CDCl_3$) δ 8.02 (dd, *J* = 7.9, 1.8 Hz, 1H), 7.75 (s, 1H), 7.50 (ddd, *J* = 8.7, 7.2, 1.8 Hz, 1H), 7.32 (d, *J* = 2.1 Hz, 1H), 7.17 (dd, *J* = 8.5, 2.1 Hz, 1H), 7.13 – 7.05 (m, 2H), 6.98 (d, *J* = 8.3 Hz, 1H), 5.92 (s, 1H), 5.34 (d, *J* = 1.9 Hz, 2H). ^{13}**C{H} NMR** (101 MHz, DMSO-d_6) δ 181.3, 160.9, 155.1, 136.5, 136.0, 132.8, 131.2, 129.4, 127.6, 126.4, 122.3, 121.9, 120.7, 118.3, 117.2, 67.8. HRMS: calculated $[M + Na]^+$ = 309.0289, experimental $[M + Na]^+$ = 309.0284, error: -1.62 ppm.

(E)-3-(3-bromo-4-hydroxybenzylidene)chroman-4-one ***(19)***: The compound was purified by recrystallization from hot methanol as yellow solid (195 mg, 59% yield). The purity (98%) was determined by HPLC analysis using a Waters C18 column (250 x 4.6 mm, 5 µm), CH_3CN/H_2O 80:20, 1 mL/min, 28 ºC, t_R: 5.2 min, 254 nm. **^{1}H NMR** (400 MHz, $CDCl_3$) δ 8.02 (dd, *J* = 7.9, 1.8 Hz, 1H), 7.75 (s, 1H), 7.55 – 7.43 (m, 2H), 7.20 (dd, *J* = 8.5, 2.1 Hz, 1H), 7.14 – 7.05 (m, 2H), 6.98 (d, *J* = 8.3 Hz, 1H), 5.92 (s, 1H), 5.34 (d, *J* = 2.0 Hz, 2H). **^{13}C{H} NMR** (126 MHz, $CDCl_3$) δ 181.8, 160.8, 155.4, 136.0, 135.6, 134.8, 130.8, 129.1, 127.6, 127.0, 121.8, 121.7, 117.7, 116.5, 110.3, 67.5. HRMS: calculated $[M + H]^+$ = 330.9964, experimental $[M + H]^+$ = 330.9974, error: 3.02 ppm.

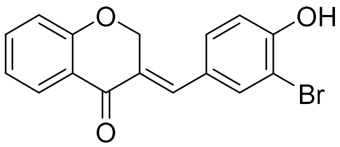

(E)-3-(3-bromo-4-methoxybenzylidene)chroman-4-one ***(20)***: The compound was purified by recrystallization from hot methanol as white solid (79 mg, 23% yield). The purity (99%) was determined by HPLC analysis using a Waters C18 column (250 x 4.6 mm, 5 µm), CH_3CN/H_2O 80:20, 1 mL/min, 28 ºC, t_R: 6.5 min, 254 nm. **^{1}H NMR** (500 MHz, $CDCl_3$) δ 8.01 (dd, *J* = 7.9, 1.7 Hz, 1H), 7.75 (t, *J* = 1.9 Hz, 1H), 7.53 (d, *J* = 2.2 Hz, 1H), 7.49 (ddd, *J* = 8.3, 7.2, 1.8 Hz, 1H), 7.28 – 7.24 (m, 1H), 7.07 (ddd, *J* = 8.0, 7.2, 1.1 Hz, 1H), 6.97 (dd, *J* = 8.4, 3.3 Hz, 2H), 5.35 (d, *J* = 1.9 Hz, 2H), 3.95 (s, 3H). **^{13}C{H} NMR** (126 MHz, $CDCl_3$) δ 182.0, 161.1, 157.0, 136.0, 135.8, 135.0, 131.0, 130.3, 128.4, 128.1, 122.1, 122.1, 118.0, 112.2, 111.9, 67.7, 56.5.

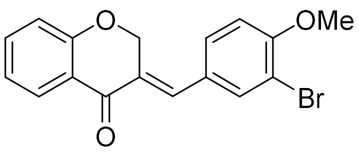

(E)-3-(4-(1H-imidazol-1-yl)benzylidene)chroman-4-one ***(21)***: The compound was obtained as white solid (148 mg, 49% yield). **^{1}H NMR** (500 MHz, DMSO-d_6) δ 9.72 (s, 1H), 8.34 (s, 1H), 7.96 (d, *J* = 8.3 Hz, 2H), 7.87 (d, *J* = 7.8 Hz, 1H), 7.84 (s, 1H), 7.78 (s, 1H), 7.69 (d, *J* = 8.4 Hz, 2H), 7.59 (t, *J* = 7.8 Hz, 1H), 7.12 (t, *J* = 7.5 Hz, 1H), 7.04 (d, *J* = 8.3 Hz, 1H), 5.43 (d, *J* = 2.1 Hz, 2H). **^{13}C{H} NMR** (126 MHz, DMSO-d_6) δ 181.0, 160.6, 136.4, 135.6, 134.9, 134.7, 134.4, 131.9, 131.8, 127.3, 122.3, 122.1, 121.7, 121.3, 120.2, 118.0, 67.3.

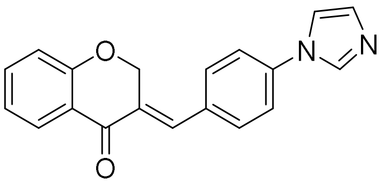

(E)-3-((5-nitrofuran-2-yl)methylene)chroman-4-one ***(22)***: The compound was obtained as an orange solid (236 mg, 87% yield). The purity (99%) was determined by HPLC analysis using a Waters C18 column (250 x 4.6 mm, 5 µm), CH_3CN/H_2O 80:20, 1 mL/min, 28 ºC, t_R: 4.3 min, 365 nm. **^{1}H NMR** (500 MHz, $CDCl_3$) δ 8.00 (dd, *J* = 7.9, 1.5 Hz, 1H), 7.54 (ddd, *J* = 8.8, 7.2, 1.7 Hz, 1H), 7.48 (t, *J* = 2.0 Hz, 1H), 7.40 (d, *J* = 3.8 Hz, 1H), 7.09

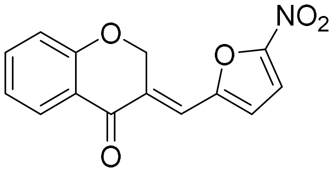

(t, J = 8.0 Hz, 1H), 7.03 (d, J = 8.3 Hz, 1H), 6.87 (d, J = 3.8 Hz, 1H), 5.67 (d, J = 1.9 Hz, 2H). **^{13}C{H} NMR** (126 MHz, $CDCl_3$) δ 180.7, 161.7, 152.7, 136.6, 133.6, 128.1, 122.4, 121.5, 119.2, 118.8, 118.4, 113.0, 68.0, 29.8. HRMS: calculated $[M + Na]^+$ = 294.0373, experimental $[M + Na]^+$ = 294.0375, error: 0.68 ppm.

*(E)-3-((5-nitrothiophen-2-yl)methylene)chroman-4-one (**23**):* The compound was purified by

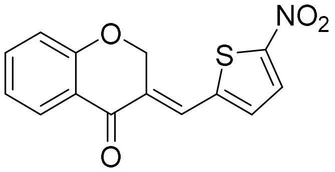

recrystallization from hot methanol as an orange solid (215 mg, 75% yield). The purity (95%) was determined by HPLC analysis using a Waters C18 column (250 x 4.6 mm, 5 µm), CH_3CN/H_2O 80:20, 1 mL/min, 28 ºC, t_R: 5.2 min, 365 nm. **^{1}H NMR** (500 MHz, DMSO-d_6) δ 8.23 (d, J = 4.4 Hz, 1H), 7.92 – 7.87 (m, 2H), 7.68 – 7.61 (m, 2H), 7.16 (ddd, J = 8.0, 7.1, 1.0 Hz, 1H), 7.11 (dd, J = 8.3, 1.0 Hz, 1H), 5.55 (d, J = 2.2 Hz, 2H). **^{13}C{H} NMR** (126 MHz, DMSO-d_6) δ 179.8, 160.7, 153.2, 143.3, 136.7, 133.2, 132.9, 130.3, 127.3, 126.3, 122.2, 121.0, 118.0, 67.5. HRMS: calculated $[M + H]^+$ = 288.0325, experimental $[M + H]^+$ = 288.0317, error: -2.78 ppm.

*(E)-3-((5-(4-nitrophenyl)furan-2-yl)methylene)chroman-4-one (**24**):* The compound was purified

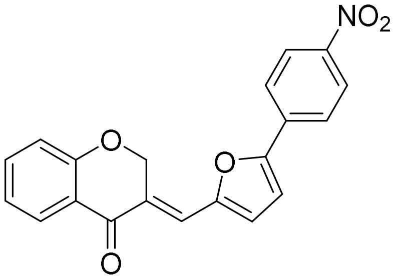

by flash chromatography (n-Hexane/EtOAc 80:20) (73 mg, 21% yield). **^{1}H NMR** (500 MHz, $CDCl_3$) δ 8.32 (d, J = 8.9 Hz, 2H), 8.03 (dd, J = 7.9, 1.8 Hz, 1H), 7.86 (d, J = 8.9 Hz, 2H), 7.57 (t, J = 2.0 Hz, 1H), 7.52 (ddd, J = 8.6, 7.3, 1.7 Hz, 1H), 7.10 (t, J = 7.5 Hz, 1H), 7.05 – 7.01 (m, 2H), 6.91 (d, J = 3.7 Hz, 1H), 5.73 (d, J = 2.0 Hz, 2H). **^{13}C{H} NMR** (126 MHz, $CDCl_3$) δ 181.3, 161.4, 154.5, 152.7, 147.3, 136.1, 135.2, 128.2, 128.1, 124.8, 124.7, 122.2, 121.1, 120.9, 118.1, 111.7, 77.2, 68.2.

*(E)-6-chloro-3-(4-chlorobenzylidene)chroman-4-one (**25**):* The compound was purified by

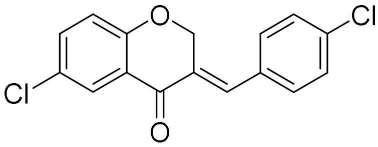

recrystallization from hot methanol as white solid (119 mg, 39% yield). **^{1}H NMR** (500 MHz, $CDCl_3$) δ 7.97 (d, J = 2.7 Hz, 1H), 7.82 (s, 1H), 7.43 (dd, J = 8.7, 2.9 Hz, 3H), 7.24 (d, J = 8.4 Hz, 2H), 6.94 (d, J = 8.8 Hz, 1H), 5.31 (d, J = 1.9 Hz, 2H). **^{13}C{H} NMR** (126 MHz, $CDCl_3$) δ 181.1, 159.6, 137.0, 136.0, 136.0, 132.7, 131.4, 130.7, 129.3, 127.7, 127.4, 122.8, 119.8, 67.7. The spectroscopic data were consistent with those reported in the literature.[14]

*(E)-6-chloro-3-(4-methoxybenzylidene)chroman-4-one (**26**):* The compound was purified by

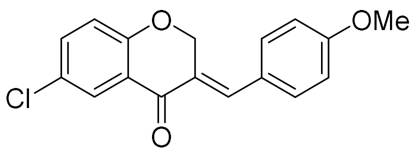

recrystallization from hot methanol as white solid (123 mg, 41% yield). The purity (99%) was determined by HPLC analysis using a Waters C18 column (250 x 4.6 mm, 5 µm),

CH_3CN/H_2O 80:20, 1 mL/min, 28 ºC, t_R: 7.3 min, 254 nm. **^{1}H NMR** (500 MHz, $CDCl_3$) δ 7.96 (d, *J* = 2.6 Hz, 1H), 7.84 (s, 1H), 7.40 (dd, *J* = 8.8, 2.7 Hz, 1H), 7.28 (d, *J* = 8.3 Hz, 2H), 6.97 (d, *J* = 8.8 Hz, 2H), 6.92 (d, *J* = 8.8 Hz, 1H), 5.37 (d, *J* = 1.9 Hz, 2H), 3.86 (s, 3H). **^{13}C{H} NMR** (126 MHz, $CDCl_3$) δ 181.2, 161.1, 159.5, 138.3, 135.6, 132.3, 128.1, 127.4, 127.3, 126.9, 123.0, 119.7, 114.5, 68.0, 55.6. The spectroscopic data were consistent with those reported in the literature.[15]

*(E)-3-(4-chlorobenzylidene)-6-fluorochroman-4-one (**27**):* The compound was purified by recrystallization from hot methanol as white solid (124 mg, 43% yield). The purity (99%) was determined by HPLC analysis using a Waters C18 column (250 x 4.6 mm, 5 µm), CH_3CN/H_2O 80:20, 1 mL/min, 28 ºC, t_R: 7.1 min, 365 nm. **^{1}H NMR** (500 MHz, $CDCl_3$) δ 7.81 (s, 1H), 7.66 (dd, *J* = 8.3, 3.2 Hz, 1H), 7.43 (d, *J* = 8.4 Hz, 2H), 7.24 (d, *J* = 8.4 Hz, 2H), 7.22 – 7.18 (m, 1H), 6.95 (dd, *J* = 9.0, 4.2 Hz, 1H), 5.28 (d, *J* = 2.0 Hz, 2H). **^{13}C{H} NMR** (126 MHz, $CDCl_3$) δ 181.4, 157.9 (d, $^1J_{C-F}$ = 242.5 Hz), 157.5, 136.8, 136.0, 132.9, 131.3, 131.0, 129.3, 123.6 (d, $^2J_{C-F}$ = 24.6 Hz), 122.7, 119.8 (d, $^3J_{C-F}$ = 7.3 Hz), 113.1 (d, $^2J_{C-F}$ = 23.4 Hz), 67.8. HRMS: calculated $[M + H]^+$ = 289.0426, experimental $[M + H]^+$ = 289.0419, error: -2.42 ppm.

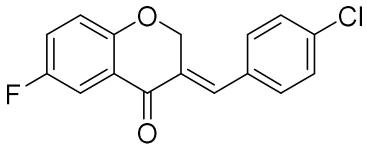

*(E)-6-fluoro-3-(4-methoxybenzylidene)chroman-4-one (**28**):* The compound was purified by recrystallization from hot methanol as white solid (110 mg, 39% yield). The purity (99%) was determined by HPLC analysis using a Waters C18 column (250 x 4.6 mm, 5 µm), CH_3CN/H_2O 80:20, 1 mL/min, 28 ºC, t_R: 5.8 min, 254 nm. **^{1}H NMR** (500 MHz, $CDCl_3$) δ 7.84 (s, 1H), 7.65 (dd, *J* = 8.4, 3.2 Hz, 1H), 7.28 (d, *J* = 8.7 Hz, 2H), 7.19 (ddd, *J* = 9.0, 7.8, 3.2 Hz, 1H), 6.97 (d, *J* = 8.8 Hz, 2H), 6.94 (dd, *J* = 9.0, 4.2 Hz, 1H), 5.35 (d, *J* = 1.9 Hz, 2H), 4.00 – 3.52 (m, 3H). **^{13}C{H} NMR** (126 MHz, $CDCl_3$) δ 181.6, 161.0, 157.7 (d, $^1J_{C-F}$ = 241.7 Hz), 157.3 (d, $^4J_{C-F}$ = 1.6 Hz), 138.1, 132.3, 128.3, 127.0, 123.2 (d, $^2J_{C-F}$ = 24.5 Hz), 122.8 (d, $^3J_{C-F}$ = 6.8 Hz), 119.6 (d, $^3J_{C-F}$ = 7.4 Hz), 114.5, 113.0 (d, $^2J_{C-F}$ = 23.9 Hz), 68.0, 55.5. HRMS: calculated $[M + H]^+$ = 285.0921, experimental $[M + H]^+$ = 285.0921, error: 0.00 ppm.

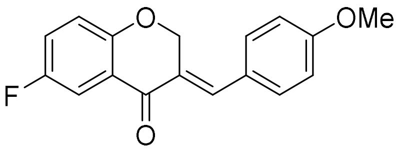

*(E)-6-bromo-3-(4-chlorobenzylidene)chroman-4-one (**29**):* The compound was purified by recrystallization from hot methanol as white solid (66 mg, 19% yield). The purity (99%) was determined by HPLC analysis using a Waters C18 column (250 x 4.6 mm, 5 µm), CH_3CN/H_2O 80:20, 1 mL/min, 28 ºC, t_R: 9.9 min, 365 nm. **^{1}H NMR** (500 MHz, $CDCl_3$) δ 8.12 (d, *J* = 2.5 Hz, 1H), 7.81 (s, 1H), 7.56 (dd, *J* = 8.8, 2.6 Hz, 1H), 7.43 (d, *J* = 8.4 Hz, 2H), 7.24 (d, *J* = 8.2 Hz, 2H), 6.87 (d, *J* = 8.8 Hz, 1H), 5.30 (d, *J* = 1.9 Hz, 2H). **^{13}C{H} NMR** (126 MHz, $CDCl_3$) δ 180.9, 160.2, 138.7, 137.0, 136.1, 132.8, 131.4, 130.7,

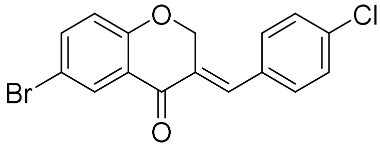

130.5, 129.3, 123.4, 120.2, 114.9, 67.8. HRMS: calculated [M + H]$^+$ = 348.9625, experimental [M + H]$^+$ = 348.9629, error: 1.15 ppm.

*(E)-6-bromo-3-(4-methoxybenzylidene)chroman-4-one (**30**):* The compound was purified by

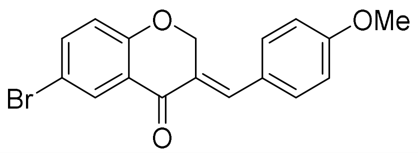

recrystallization from hot methanol as yellow solid (200 mg, 58% yield). The purity (98%) was determined by HPLC analysis using a Waters C18 column (250 x 4.6 mm, 5 µm), CH_3CN/H_2O 80:20, 1 mL/min, 28 ºC, t_R: 7.9 min, 254 nm. **^{1}H NMR** (500 MHz, $CDCl_3$) δ 8.10 (d, *J* = 2.6 Hz, 1H), 7.83 (s, 1H), 7.53 (dd, *J* = 8.8, 2.6 Hz, 1H), 7.27 (d, *J* = 8.8 Hz, 2H), 6.96 (d, *J* = 8.9 Hz, 2H), 6.85 (d, *J* = 8.7 Hz, 1H), 5.36 (d, *J* = 1.9 Hz, 2H), 3.86 (s, 3H). **^{13}C{H} NMR** (126 MHz, $CDCl_3$) δ 181.0, 161.1, 159.9, 138.3, 138.3, 132.3, 130.4, 128.0, 126.9, 123.5, 120.0, 114.6, 114.5, 68.0, 55.5. The spectroscopic data were consistent with those reported in the literature.[16]

*(E)-7-methoxy-3-(4-methoxybenzylidene)chroman-4-one (**31**):* The compound was obtained as a

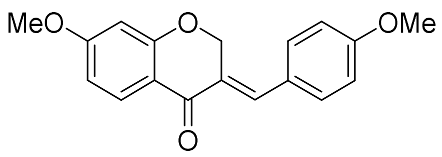

pale-yellow solid (240 mg, 81% yield). The purity (98%) was determined by HPLC analysis using a Waters C18 column (250 x 4.6 mm, 5 µm), CH_3CN/H_2O 80:20, 1 mL/min, 28 ºC, t_R: 5.2 min, 365 nm. **^{1}H NMR** (400 MHz, $CDCl_3$) δ 7.96 (d, *J* = 8.8 Hz, 1H), 7.80 (s, 1H), 7.27 (d, *J* = 6.3 Hz, 2H), 6.96 (d, *J* = 8.7 Hz, 2H), 6.62 (dd, *J* = 8.8, 2.4 Hz, 1H), 6.40 (d, *J* = 2.4 Hz, 1H), 5.36 (d, *J* = 1.7 Hz, 2H), 3.85 (d, *J* = 6.1 Hz, 6H). **^{13}C{H} NMR** (101 MHz, $CDCl_3$) δ 181.0, 165.9, 162.9, 160.5, 136.6, 131.9, 130.0, 129.7, 128.9, 127.2, 115.8, 114.4, 114.2, 110.3, 100.7, 68.0, 55.6, 55.4. The spectroscopic data were consistent with those reported in the literature.[17]

*(E)-3-(3,4-dimethoxybenzylidene)-7-methoxychroman-4-one (**32**):* The compound was obtained

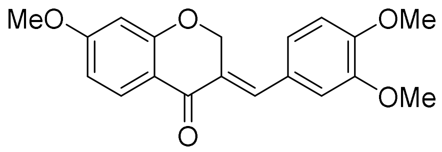

as a pale-yellow solid (310 mg, 95% yield). The purity (99%) was determined by HPLC analysis using a Waters C18 column (250 x 4.6 mm, 5 µm), CH_3CN/H_2O 80:20, 1 mL/min, 28 ºC, t_R: 4.5 min, 254 nm. **^{1}H NMR** (400 MHz, $CDCl_3$) δ 7.96 (d, *J* = 8.8 Hz, 1H), 7.79 (s, 1H), 6.95 – 6.84 (m, 3H), 6.63 (dd, *J* = 8.8, 2.4 Hz, 1H), 6.40 (d, *J* = 2.4 Hz, 1H), 5.37 (d, *J* = 1.8 Hz, 2H), 3.93 (s, 3H), 3.91 (s, 3H), 3.84 (s, 3H). **^{13}C{H} NMR** (101 MHz, $CDCl_3$) δ 180.8, 165.9, 162.9, 150.2, 148.9, 136.7, 129.6, 129.2, 127.4, 123.4, 115.8, 113.2, 111.0, 110.3, 100.8, 68.0, 56.0, 55.6. HRMS: calculated [M + H]$^+$ = 327.1227, experimental [M + H]$^+$ = 327.1231, error: 1.22 ppm.

*(E)-7-hydroxy-3-(4-methoxybenzylidene)chroman-4-one (**33**):* The compound was obtained as a pale-yellow solid (175 mg, 62% yield). The purity (99%) was determined by HPLC analysis using a Waters C18 column (250 x 4.6 mm, 5 µm), CH_3CN/H_2O 80:20, 1 mL/min, 28 ºC, t_R: 4.2 min, 254 nm. **^{1}H NMR** (500 MHz, DMSO-d_6) δ 10.64 (s, 1H), 7.74 (d, J = 8.7 Hz, 1H), 7.64 (s, 1H), 7.41 (d, J = 8.8 Hz, 2H), 7.05 (d, J = 8.8 Hz, 2H), 6.55 (dd, J = 8.7, 2.3 Hz, 1H), 6.32 (d, J = 2.2 Hz, 1H), 5.36 (d, J = 1.9 Hz, 2H), 3.82 (s, 3H). **^{13}C{H} NMR** (126 MHz, DMSO-d_6) δ 179.5, 164.7, 162.5, 160.3, 135.2, 132.2, 129.5, 128.9, 126.6, 114.3, 114.3, 111.2, 102.5, 67.6, 55.3. The spectroscopic data were consistent with those reported in the literature.[18]

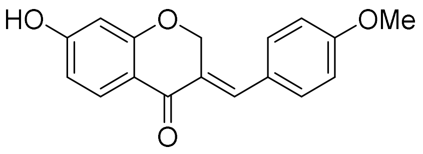

*(E)-3-(3,4-dimethoxybenzylidene)-7-hydroxychroman-4-one (**34**):* The compound was obtained as a pale-yellow solid (193 mg, 62% yield). The purity (95%) was determined by HPLC analysis using a Waters C18 column (250 x 4.6 mm, 5 µm), CH_3CN/H_2O 80:20, 1 mL/min, 28 ºC, t_R: 3.7 min, 365 nm. **^{1}H NMR** (500 MHz, DMSO-d_6) δ 7.74 (d, J = 8.7 Hz, 1H), 7.64 (s, 1H), 7.10 – 7.03 (m, 2H), 6.98 (dd, J = 8.4, 2.0 Hz, 1H), 6.55 (dd, J = 8.7, 2.1 Hz, 1H), 6.32 (d, J = 2.2 Hz, 1H), 5.40 (d, J = 1.9 Hz, 2H), 3.81 (d, J = 5.6 Hz, 6H). **^{13}C{H} NMR** (126 MHz, DMSO-d_6) δ 179.5, 164.6, 162.5, 150.1, 148.7, 135.7, 129.4, 129.0, 126.7, 123.7, 114.3, 113.9, 111.6, 111.2, 102.5, 67.6, 55.6 The spectroscopic data were consistent with those reported in the literature.[19]

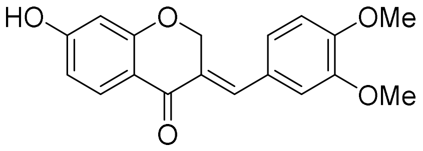

*(E)-5,7-dimethoxy-3-(4-methoxybenzylidene)chroman-4-one (**35**):* The compound was purified by flash chromatography (*n*-Hexane/EtOAc 80:20) as a pale-yellow solid (107 mg, 33% yield). The purity (97%) was determined by HPLC analysis using a Waters C18 column (250 x 4.6 mm, 5 µm), CH_3CN/H_2O 80:20, 1 mL/min, 28 ºC, t_R: 4.1 min, 365 nm. **^{1}H NMR** (400 MHz, $CDCl_3$) δ 7.77 (s, 1H), 7.24 (d, J = 8.6 Hz, 2H), 6.95 (d, J = 8.8 Hz, 2H), 6.13 (d, J = 2.3 Hz, 1H), 6.07 (d, J = 2.3 Hz, 1H), 5.24 (d, J = 1.8 Hz, 2H), 3.91 (s, 3H), 3.85 (s, 3H), 3.83 (s, 3H). **^{13}C{H} NMR** (101 MHz, $CDCl_3$) δ 179.6, 165.7, 164.6, 162.8, 160.3, 135.7, 131.7, 130.1, 127.4, 114.1, 107.3, 93.6, 67.6, 56.2, 55.6, 55.4, 29.7. The spectroscopic data were consistent with those reported in the literature.[20]

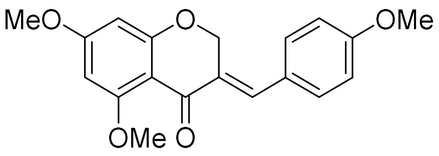

*(E)-5,6,7-trimethoxy-3-(4-methoxybenzylidene)chroman-4-one (**36**):* The compound was purified by flash chromatography (*n*-Hexane/$CHCl_3$/EtOAc 40:20:5) as a white solid (164 mg, 46% yield). The purity (99%) was determined by HPLC analysis using a Waters C18 column (250 x 4.6 mm, 5 µm), CH_3CN/H_2O 80:20, 1 mL/min, 28 ºC, t_R: 4.4 min, 254 nm. **^{1}H NMR** (500 MHz, DMSO-d_6) δ 7.63 (s, 1H), 7.40 (d, J

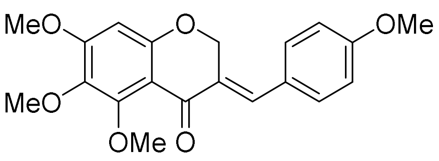

= 8.7 Hz, 2H), 7.04 (d, J = 8.7 Hz, 2H), 6.47 (s, 1H), 5.27 (s, 2H), 3.85 (s, 3H), 3.82 (d, J = 4.0 Hz, 6H), 3.69 (s, 3H). **^{13}C NMR** (126 MHz, DMSO-d_6) δ 178.75, 160.74, 159.37, 159.28, 154.43, 137.71, 135.50, 132.57, 130.17, 126.98, 114.77, 110.44, 97.10, 67.72, 61.71, 61.21, 56.71, 55.79. HRMS: calculated $[M + H]^+$ = 357.1333, experimental $[M + H]^+$ = 357.1335, error: 0.56 ppm.

*(E)-3-(2-hydroxybenzylidene)-5,7-dimethoxychroman-4-one (**37**):* The compound was purified by

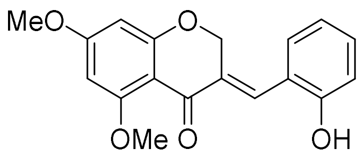

flash chromatography (*n*-Hexane/EtOAc 80:20) as a yellow solid (165 mg, 53% yield). **^{1}H NMR** (400 MHz, $CDCl_3$) δ 9.45 (s, 1H), 7.23 (d, *J* = 6.9 Hz, 2H), 7.04 (d, *J* = 8.3 Hz, 1H), 6.99 (t, *J* = 7.4 Hz, 1H), 6.77 (s, 1H), 6.24 (d, *J* = 2.3 Hz, 1H), 6.12 (d, *J* = 2.3 Hz, 1H), 4.79 – 4.65 (m, 2H), 4.00 (s, 3H), 3.78 (s, 3H). **^{13}C NMR** (101 MHz, Acetone-d_6) δ 162.6, 160.9, 157.6, 153.5, 130.0, 129.1, 127.8, 123.1, 122.4, 122.2, 117.9, 108.5, 94.8, 94.4, 92.5, 68.4, 56.6, 55.8. The spectroscopic data were consistent with those reported in the literature.[1]

*(E)-5-hydroxy-7-methoxy-3-(4-methoxybenzylidene)chroman-4-one (**38**):* The compound was

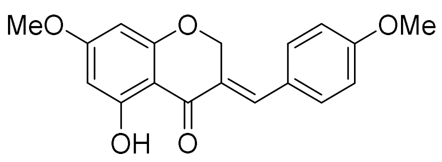

obtained as a white solid (200 mg, 64% yield). The purity (97%) was determined by HPLC analysis using a Waters C18 column (250 x 4.6 mm, 5 µm), CH_3CN/H_2O 80:20, 1 mL/min, 28 ºC, t_R: 6.4 min, 365 nm. **^{1}H NMR** (500 MHz, $CDCl_3$) δ 12.76 (s, 1H), 7.80 (s, 1H), 7.26 (d, J = 8.6 Hz, 2H), 6.97 (d, J = 8.6 Hz, 2H), 6.02 (dd, J = 61.1, 2.1 Hz, 2H), 5.31 (s, 2H), 3.86 (s, 3H), 3.81 (s, 3H). **^{13}C NMR** (101 MHz, $CDCl_3$) δ 185.36, 168.01, 165.27, 162.17, 160.99, 137.19, 132.22, 127.66, 126.97, 114.46, 95.23, 94.98, 94.12, 77.16, 67.57, 55.79, 55.55. The spectroscopic data were partially consistent with those reported in the literature.[21]

*(E)-3-(2-hydroxybenzylidene)-5,6,7-trimethoxychroman-4-one (**39**):* The compound was purified

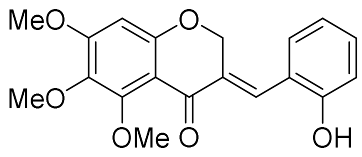

by flash chromatography (*n*-Hexane/$CHCl_3$/EtOAc 40:20:5) as a white solid (226 mg, 66% yield). **^{1}H NMR** (500 MHz, $CDCl_3$) 7.24 (d, J = 7.2 Hz, 2H), 7.04 – 6.98 (m, 2H), 6.78 (s, 1H), 6.28 (s, 1H), 4.88 (s, 1H), 4.75 – 4.67 (m, 2H), 4.28 (s, 3H), 3.86 (s, 3H), 3.84 (s, 3H). **^{13}C NMR** (126 MHz, Acetone-d_6) δ 156.11, 153.37, 153.18, 152.03, 138.50, 130.27, 128.98, 127.99, 123.28, 122.51, 122.43, 118.03, 113.06, 97.69, 92.70, 68.42, 62.17, 60.99, 56.41.

*(E)-3-(4-chlorobenzylidene)-7-(piperidin-1-yl)chroman-4-one (**40**):* The compound was obtained

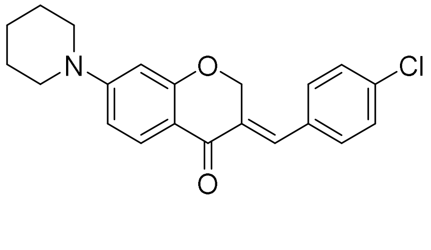

as a yellow solid (152 mg, 43% yield). The purity (99%) was determined by HPLC analysis using a Waters C18 column (250 x 4.6 mm, 5 µm), CH_3CN/H_2O 80:20, 1 mL/min, 28 ºC, t_R: 9.6 min, 388 nm. **^{1}H NMR** (400 MHz,

$CDCl_3$) δ 7.87 (d, *J* = 9.1 Hz, 1H), 7.73 (s, 1H), 7.40 (d, *J* = 8.4 Hz, 2H), 7.21 (d, *J* = 8.4 Hz, 2H), 6.58 (dd, *J* = 9.1, 2.2 Hz, 1H), 6.23 (d, *J* = 2.2 Hz, 1H), 5.24 (d, *J* = 1.5 Hz, 2H), 3.39 (s, 4H), 1.66 (s, 6H). **^{13}C{H} NMR** (101 MHz, $CDCl_3$) δ 179.9, 163.1, 156.5, 135.0, 134.1, 133.3, 132.1, 131.0, 129.6, 128.9, 112.0, 109.1, 99.2, 67.4, 48.3, 25.3, 24.4. HRMS: calculated $[M + H]^+$ = 354.1255, experimental $[M + H]^+$ = 354.1260, error: 1.41 ppm.

*(E)-3-((5-nitrofuran-2-yl)methylene)-7-(piperidin-1-yl)chroman-4-one (**41**):* The compound was obtained as a red solid (205 mg, 58% yield). The purity (97%) was determined by HPLC analysis using a Waters C18 column (250 x 4.6 mm, 5 µm), CH_3CN/H_2O 80:20, 1 mL/min, 28 °C, t_R: 5.9 min, 254 nm. **^{1}H NMR** (500 MHz, $CDCl_3$) δ 7.86 (d, *J* = 9.0 Hz, 1H), 7.43 (s, 1H), 7.38 (d, *J* = 3.8 Hz, 1H), 6.79 (d, *J* = 3.8 Hz, 1H), 6.61 (dd, *J* = 9.1, 2.5 Hz, 1H), 6.28 (d, *J* = 2.5 Hz, 1H), 5.60 (d, *J* = 2.1 Hz, 2H), 3.43 (s, 4H), 1.69 (s, 6H). **^{13}C{H} NMR** (126 MHz, $CDCl_3$) δ 177.9, 163.7, 156.7, 153.5, 134.9, 129.8, 117.7, 117.4, 113.2, 111.7, 109.3, 99.1, 68.0, 48.4, 29.8, 25.5, 24.5. HRMS: calculated $[M + H]^+$ = 377.1108, experimental $[M + H]^+$ = 377.1105, error: -0.79 ppm.

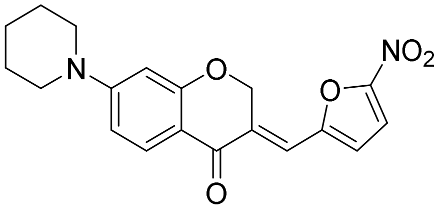

*(E)-3-((5-nitrothiophen-2-yl)methylene)-7-(piperidin-1-yl)chroman-4-one (**42**):* The compound was purified by flash chromatography (*n*-Hexane/CH_2Cl_2 40:60) as a red solid (85 mg, 23% yield). **^{1}H NMR** (500 MHz, $CDCl_3$) δ 7.92 (d, *J* = 4.3 Hz, 1H), 7.86 (d, *J* = 9.1 Hz, 1H), 7.78 (s, 1H), 7.16 (d, *J* = 4.3 Hz, 1H), 6.60 (dd, *J* = 9.1, 2.5 Hz, 1H), 6.25 (d, *J* = 2.5 Hz, 1H), 5.37 (d, *J* = 2.2 Hz, 2H), 3.43 (s, 4H), 1.68 (s, 6H). **^{13}C{H} NMR** (126 MHz, $CDCl_3$) δ 177.7, 163.2, 156.7, 144.4, 134.1, 130.9, 129.9, 129.0, 124.7, 111.8, 109.4, 99.1, 67.7, 48.4, 29.8, 25.5, 24.5. HRMS: calculated $[M + H]^+$ = 371.1060, experimental $[M + H]^+$ = 371.1067, error: 1.89 ppm.

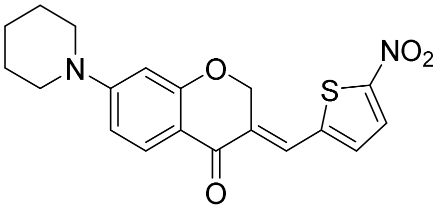

3. REFERENCES

(1) Damodar, K.; Lee, J. T.; Kim, J.-K.; Jun, J.-G. Synthesis and in Vitro Evaluation of Homoisoflavonoids as Potent Inhibitors of Nitric Oxide Production in RAW-264.7 Cells. *Bioorg Med Chem Lett* **2018**, *28* (11), 2098–2102. https://doi.org/10.1016/j.bmcl.2018.04.037.

(2) Caleffi, G. S.; Brum, J. D. O. C.; Costa, A. T.; Domingos, J. L. O.; Costa, P. R. R. Asymmetric Transfer Hydrogenation of Arylidene-Substituted Chromanones and Tetralones Catalyzed by Noyori-Ikariya Ru(II) Complexes: One-Pot Reduction of C═C and C═O Bonds. *Journal of Organic Chemistry* **2021**, *86* (6), 4849–4858. https://doi.org/10.1021/acs.joc.0c02981.

(3) Romanov-Michailidis, F.; Guénée, L.; Alexakis, A. Enantioselective Organocatalytic Iodination-Initiated Wagner–Meerwein Rearrangement. *Org Lett* **2013**, *15* (22), 5890–5893. https://doi.org/10.1021/ol402981z.

(4) Ibrahim, A. I. M.; Batlle, E.; Sneha, S.; Jiménez, R.; Pequerul, R.; Parés, X.; Rüngeler, T.; Jha, V.; Tuccinardi, T.; Sadiq, M.; Frame, F.; Maitland, N. J.; Farrés, J.; Pors, K. Expansion of the 4-(Diethylamino)Benzaldehyde Scaffold to Explore the Impact on Aldehyde Dehydrogenase Activity and Antiproliferative Activity in Prostate Cancer. *J Med Chem* **2022**, *65* (5), 3833–3848. https://doi.org/10.1021/acs.jmedchem.1c01367.

(5) Moreira, J.; Loureiro, J. B.; Correia, D.; Palmeira, A.; Pinto, M. M.; Saraiva, L.; Cidade, H. Structure–Activity Relationship Studies of Chalcones and Diarylpentanoids with Antitumor Activity: Potency and Selectivity Optimization. *Pharmaceuticals* **2023**, *16* (10), 1354. https://doi.org/10.3390/ph16101354.

(6) Li, W.; Yang, T.; Song, N.; Li, R.; Long, J.; He, L.; Zhang, X.; Lv, H. Ir/f-Ampha Complex Catalyzed Asymmetric Sequential Hydrogenation of Enones: A General Access to Chiral Alcohols with Two Contiguous Chiral Centers. *Chem Sci* **2022**, *13* (6), 1808–1814. https://doi.org/10.1039/D1SC05963G.

(7) Gopaul, K.; Koorbanally, N. A.; Shaikh, M. M.; Su, H.; Ramjugernath, D. 3-(3,4-Dichlorobenzylidene)Chroman-4-One. *Acta Crystallogr Sect E Struct Rep Online* **2012**, *68* (11), o3062–o3062. https://doi.org/10.1107/S1600536812040561.

(8) Desideri, N.; Olivieri, S.; Stein, M.; Sgro, R.; Orsi, N.; Conti, C. Synthesis and Anti-Picornavirus Activity of Homo-Isoflavonoids. *Antivir Chem Chemother* **1997**, *8* (6), 545–555. https://doi.org/10.1177/095632029700800609.

(9) Cheng, X.-M.; Huang, Z.-T.; Zheng, Q.-Y. Topochemical Photodimerization of (E)-3-Benzylidene-4-Chromanone Derivatives from β-Type Structures Directed by Halogen Groups. *Tetrahedron* **2011**, *67* (47), 9093–9098. https://doi.org/10.1016/j.tet.2011.09.087.

(10) Molina Betancourt, R.; Phansavath, P.; Ratovelomanana-Vidal, V. Rhodium-Catalyzed Asymmetric Transfer Hydrogenation/Dynamic Kinetic Resolution of 3-Benzylidene-Chromanones. *Org Lett* **2021**, *23* (5), 1621–1625. https://doi.org/10.1021/acs.orglett.1c00047.

(11) Magar, T. B. T.; Seo, S. H.; Kadayat, T. M.; Jo, H.; Shrestha, A.; Bist, G.; Katila, P.; Kwon, Y.; Lee, E.-S. Synthesis and SAR Study of New Hydroxy and Chloro-Substituted 2,4-Diphenyl 5H-Chromeno[4,3-b]Pyridines as Selective Topoisomerase IIα-Targeting Anticancer Agents. *Bioorg Med Chem* **2018**, *26* (8), 1909–1919. https://doi.org/10.1016/j.bmc.2018.02.035.

(12) Mandal, T. K.; Pal, R.; Mondal, R.; Mallik, A. K. Facile Condensation of Aromatic Aldehydes with Chroman-4-Ones and 1-Thiochroman-4-Ones Catalysed by Amberlyst-15 under Microwave Irradiation Condition. *E-Journal of Chemistry* **2011**, *8* (2), 863–869. https://doi.org/10.1155/2011/426560.

(13) Desideri, N.; Bolasco, A.; Fioravanti, R.; Proietti Monaco, L.; Orallo, F.; Yáñez, M.; Ortuso, F.; Alcaro, S. Homoisoflavonoids: Natural Scaffolds with Potent and Selective Monoamine Oxidase-B Inhibition Properties. *J Med Chem* **2011**, *54* (7), 2155–2164. https://doi.org/10.1021/jm1013709.

(14) Cheng, X.-M.; Chen, M.; Huang, Z.-T.; Zheng, Q.-Y. Stereocontrolled Photodimerization of (E)-3-Benzylidene-4-Chromanones in the Crystalline State: The Effect of a Halogen Group on the Chromanone Moiety. *Synthesis (Stuttg)* **2012**, *44* (23), 3693–3698. https://doi.org/10.1055/s-0032-1317511.

(15) Li, H.-Y.; Li, S.-N.; Wang, Q.; Wang, S.-X.; Zhu, B.-C. Imidazolium Ionic Liquids as Catalyst for Synthesis of (*E*)-3-Arylidene(Thio)Chroman-4-Ones under Microwave Irradiation. *J Chem Res* **2012**, *36* (11), 635–637. https://doi.org/10.3184/174751912X13466926675198.

(16) Gan, C.; Zhao, Z.; Nan, D.-D.; Yin, B.; Hu, J. Homoisoflavonoids as Potential Imaging Agents for β-Amyloid Plaques in Alzheimer's Disease. *Eur J Med Chem* **2014**, *76*, 125–131. https://doi.org/10.1016/j.ejmech.2014.02.020.

(17) Shaikh, M. M.; Kruger, H. G.; Bodenstein, J.; Smith, P.; du Toit, K. Anti-Inflammatory Activities of Selected Synthetic Homoisoflavanones. *Nat Prod Res* **2012**, *26* (16), 1473–1482. https://doi.org/10.1080/14786419.2011.565004.

(18) Veerapandiyan, K.; Ravichandiran, K.; N. Patra, C.; Balan, B.; Usha, B. *De Novo* Assembly and Annotation of *Caesalpinia Bonducella* L. Seed Transcriptome Identifies Key Genes in the Biosynthesis of Bonducellin, a Homoisoflavonoid. *Nat Prod Res* **2024**, 1–8. https://doi.org/10.1080/14786419.2024.2301740.

(19) Wang, Y.; Wang, H.; Yang, G.; Hao, Q.; Yang, K.; Shen, H.; Wang, Y.; Wang, J. Design and Synthesis of a Novel Class of PDE4 Inhibitors with Antioxidant Properties as Bifunctional Agents for the Potential Treatment of COPD. *Eur J Med Chem* **2023**, *256*, 115374. https://doi.org/10.1016/j.ejmech.2023.115374.

(20) Desideri, N.; Olivieri, S.; Stein, M.; Sgro, R.; Orsi, N.; Conti, C. Synthesis and Anti-Picornavirus Activity of Homo-Isoflavonoids. *Antivir Chem Chemother* **1997**, *8* (6), 545–555. https://doi.org/10.1177/095632029700800609.

(21) Heller, W.; Andermatt, P.; Schaad, W. A.; Tamm, C. Homoisoflavanone. IV. Neue Inhaltsstoffe Der Eucomin-Reihe von *Eucomis Bicolor*. *Helv Chim Acta* **1976**, *59* (6), 2048–2058. https://doi.org/10.1002/hlca.19760590618.

4. NMR SPECTRA OF THE HOMOISOFLAVONE DERIVATIVES 1-42

^{1}H NMR (500 MHz, $CDCl_3$) spectrum of 1

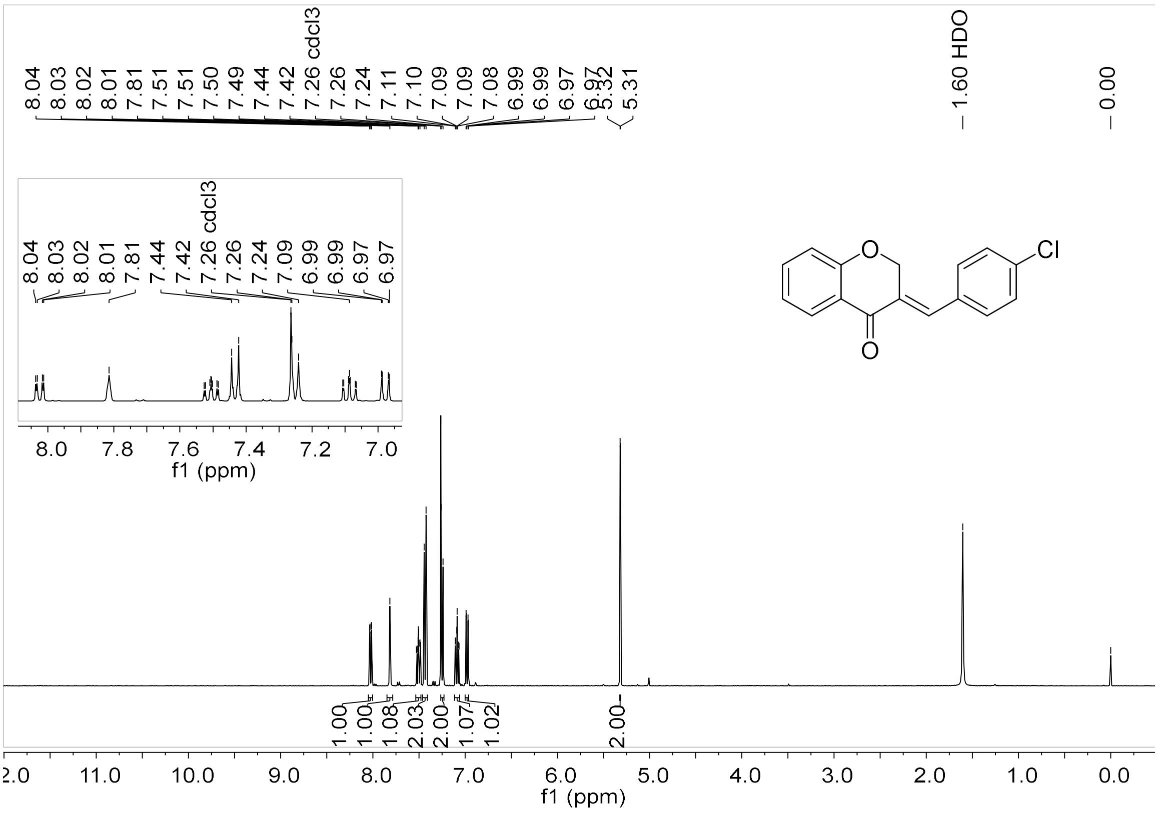

^{13}C NMR (126 MHz, $CDCl_3$) of 1

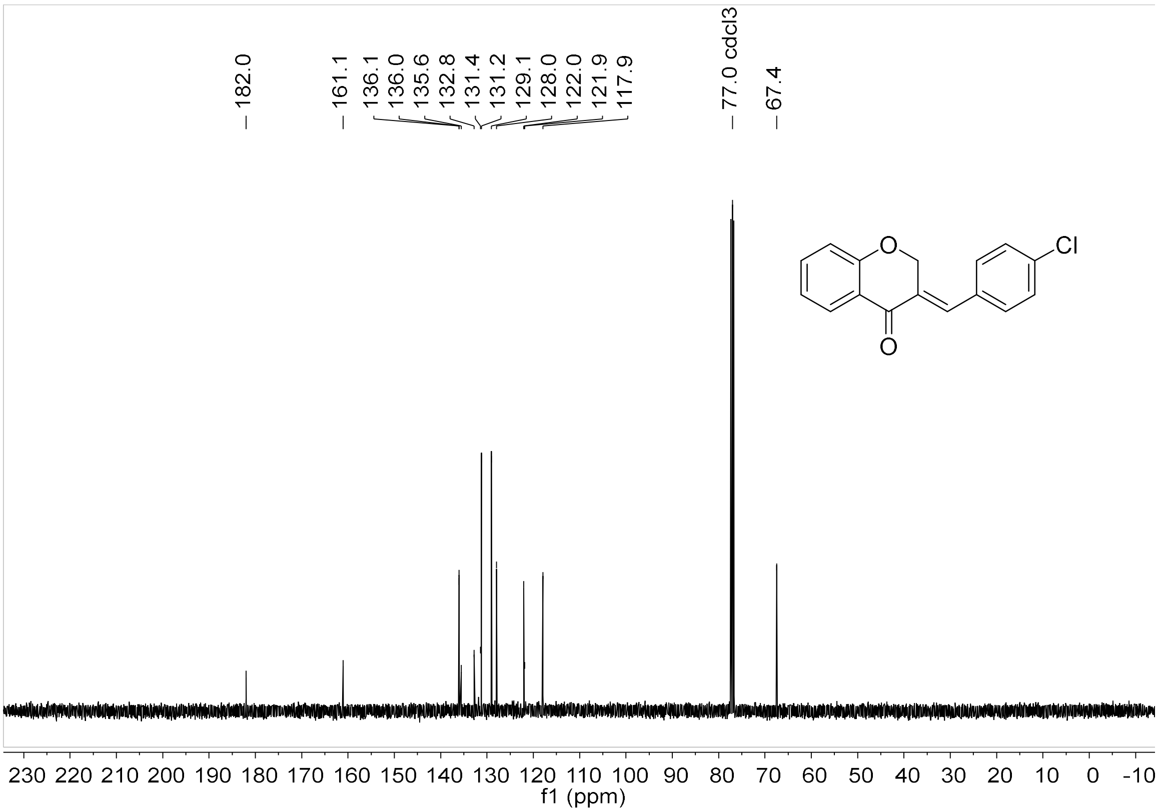

^{1}H NMR (500 MHz, $CDCl_3$) spectrum of 2

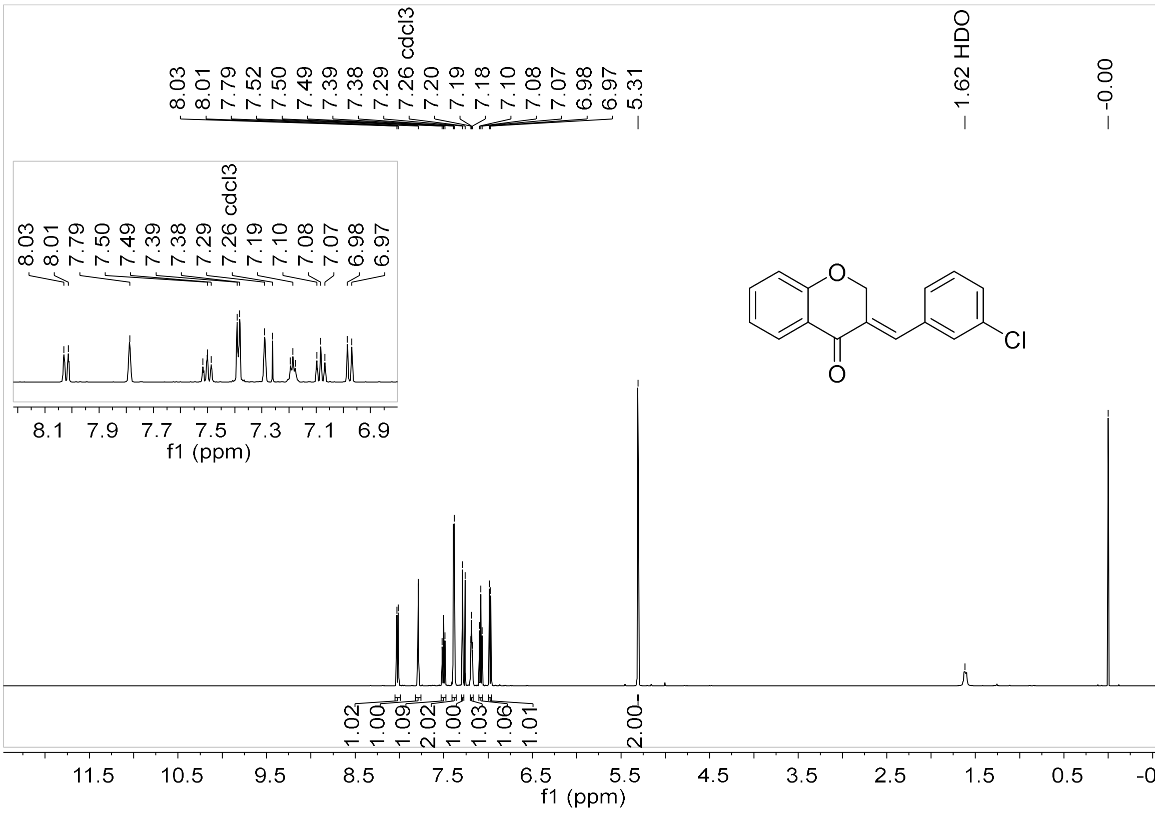

^{13}C NMR (126 MHz, $CDCl_3$) of 2

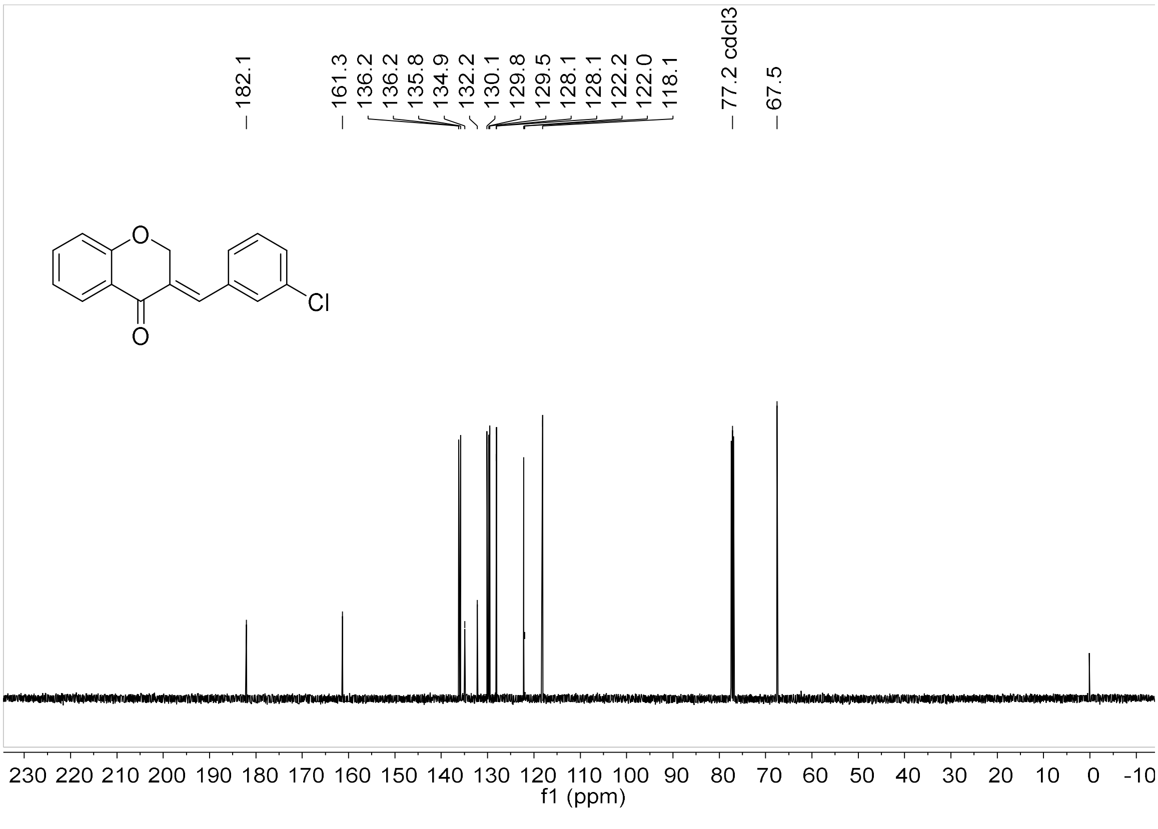

^{1}H NMR (400 MHz, $CDCl_3$) spectrum of 3

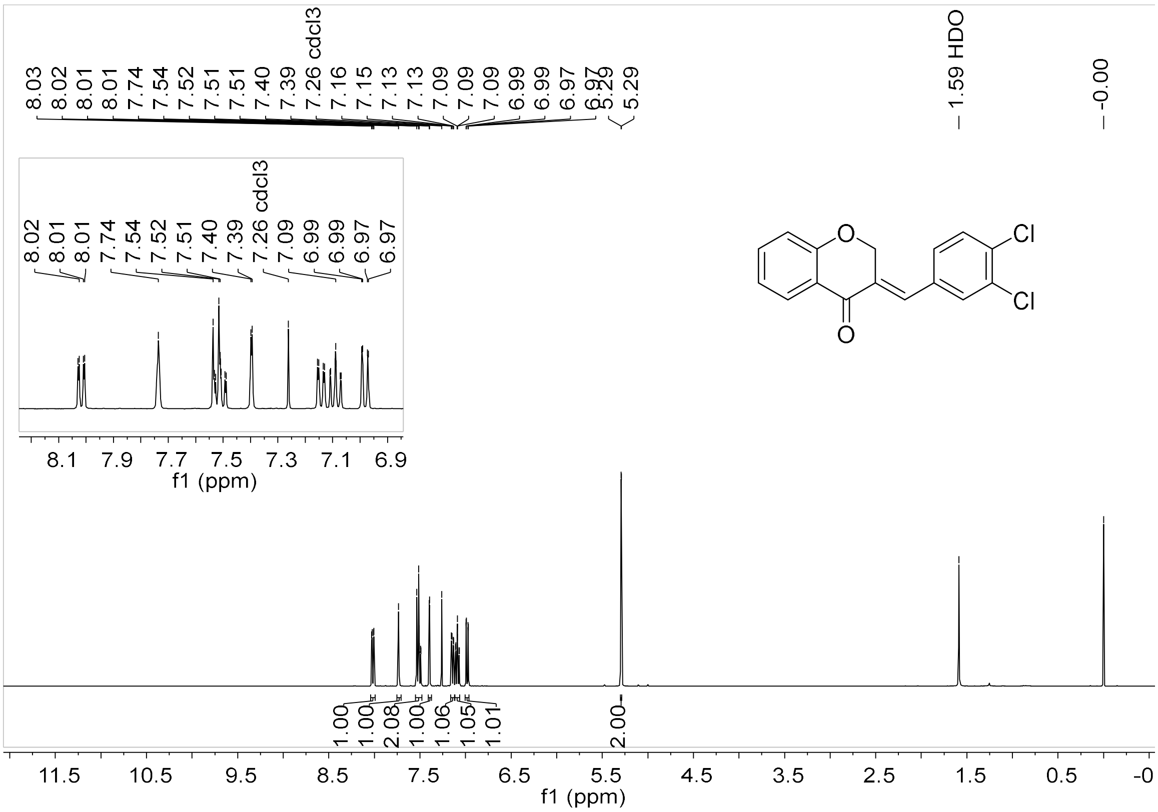

^{13}C NMR (101 MHz, $CDCl_3$) of 3

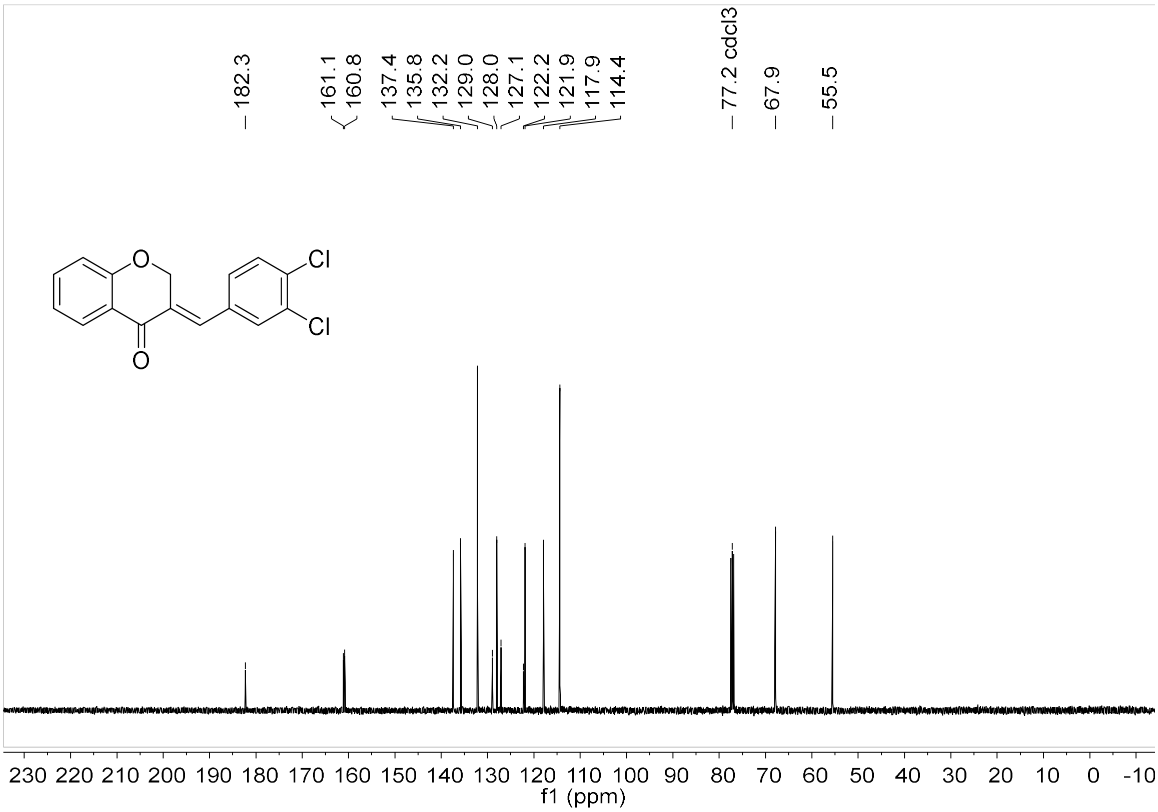

^{1}H NMR (400 MHz, $CDCl_3$) spectrum of 4

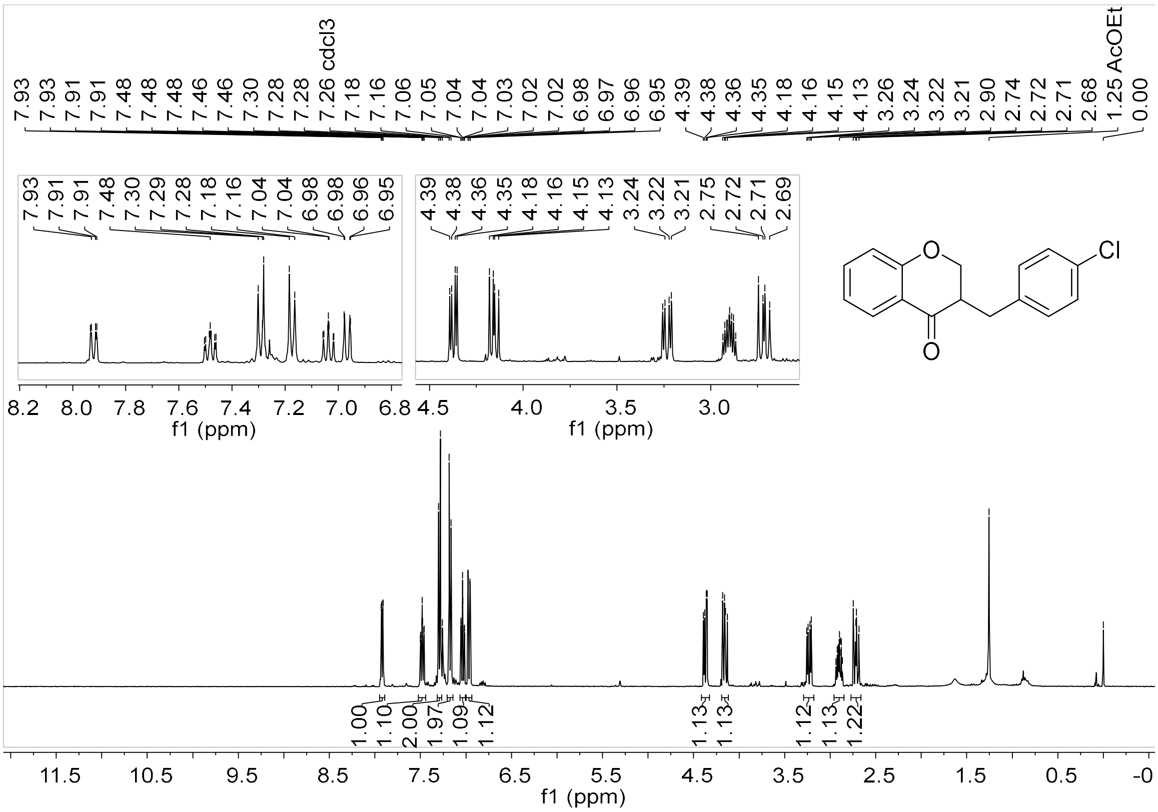

^{13}C NMR (101 MHz, $CDCl_3$) of 4

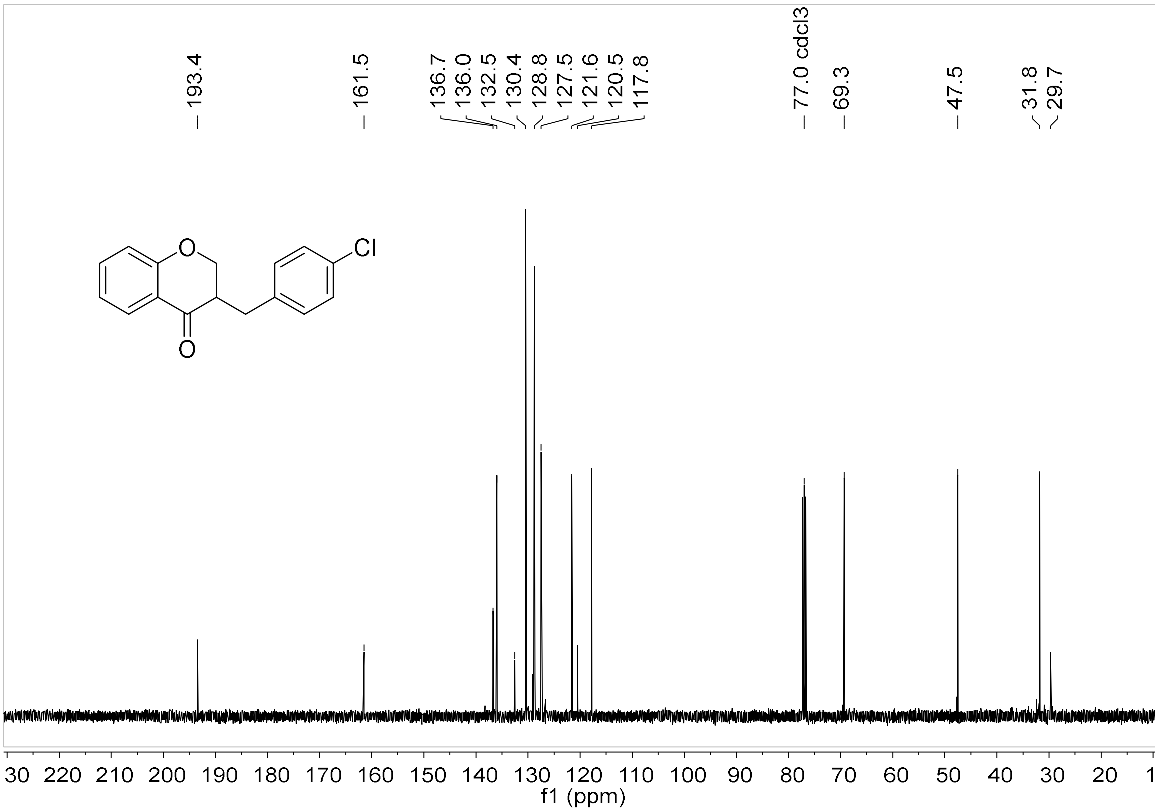

^{1}H NMR (400 MHz, $CDCl_3$) spectrum of 5

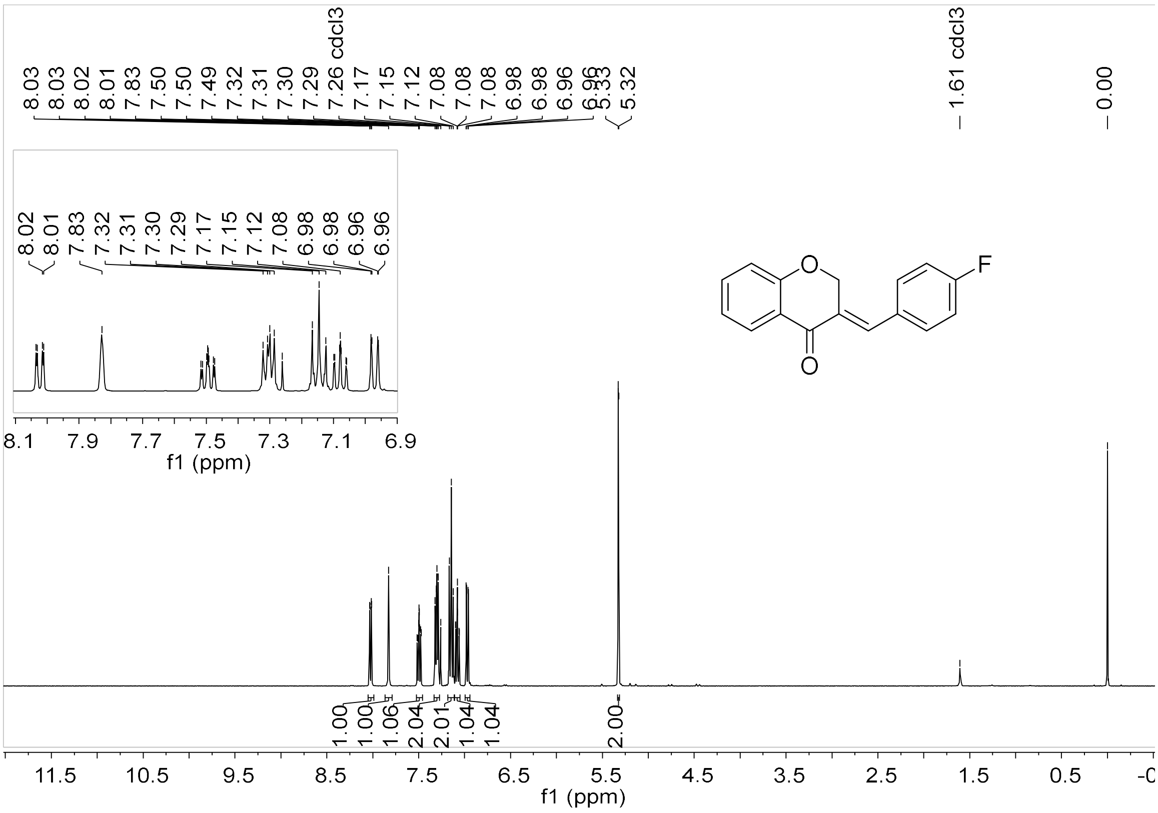

^{13}C NMR (101 MHz, $CDCl_3$) of 5

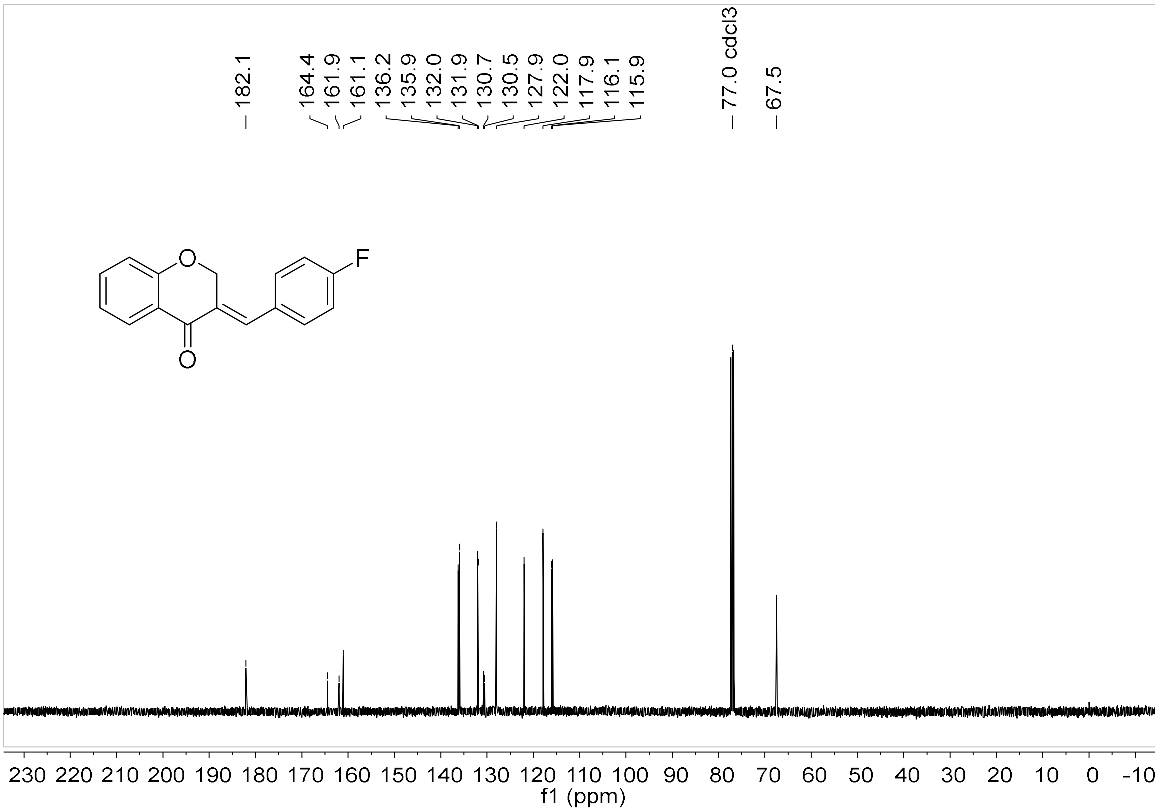

^{1}H NMR (400 MHz, CDCl$_3$) spectrum of 6

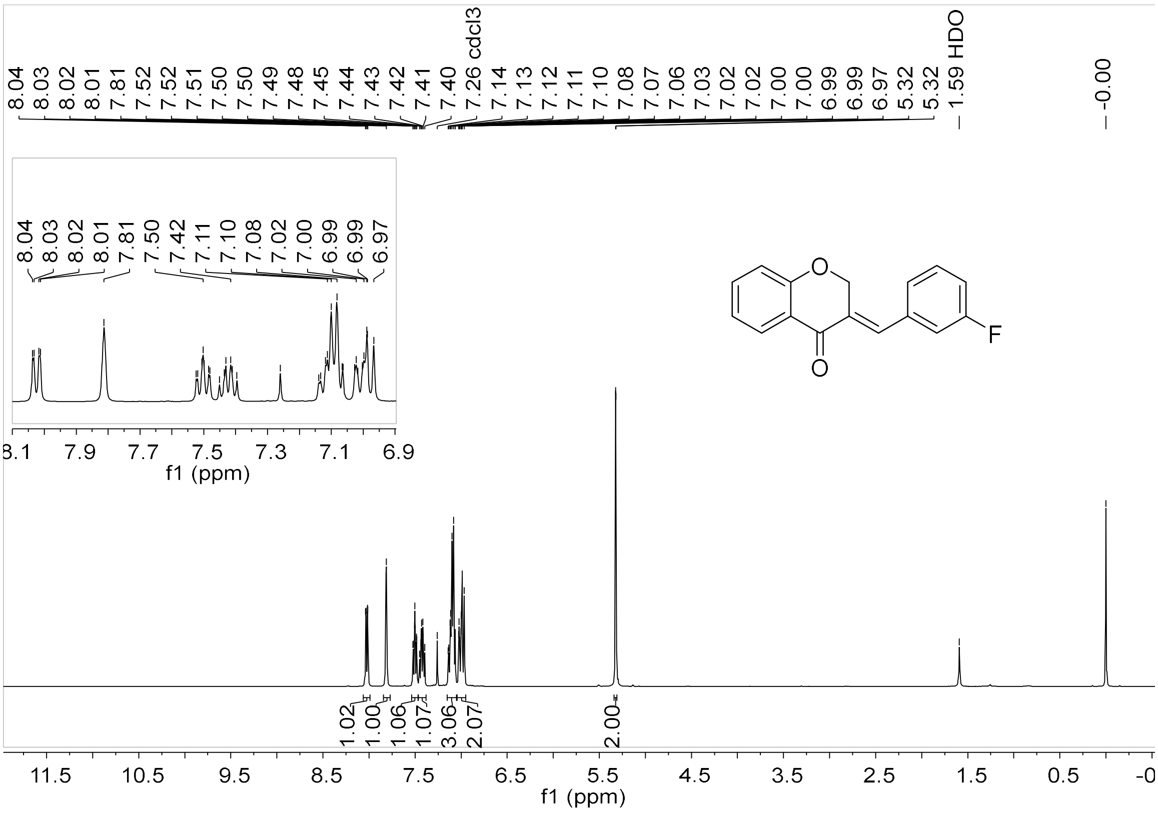

^{13}C NMR (101 MHz, CDCl$_3$) of 6

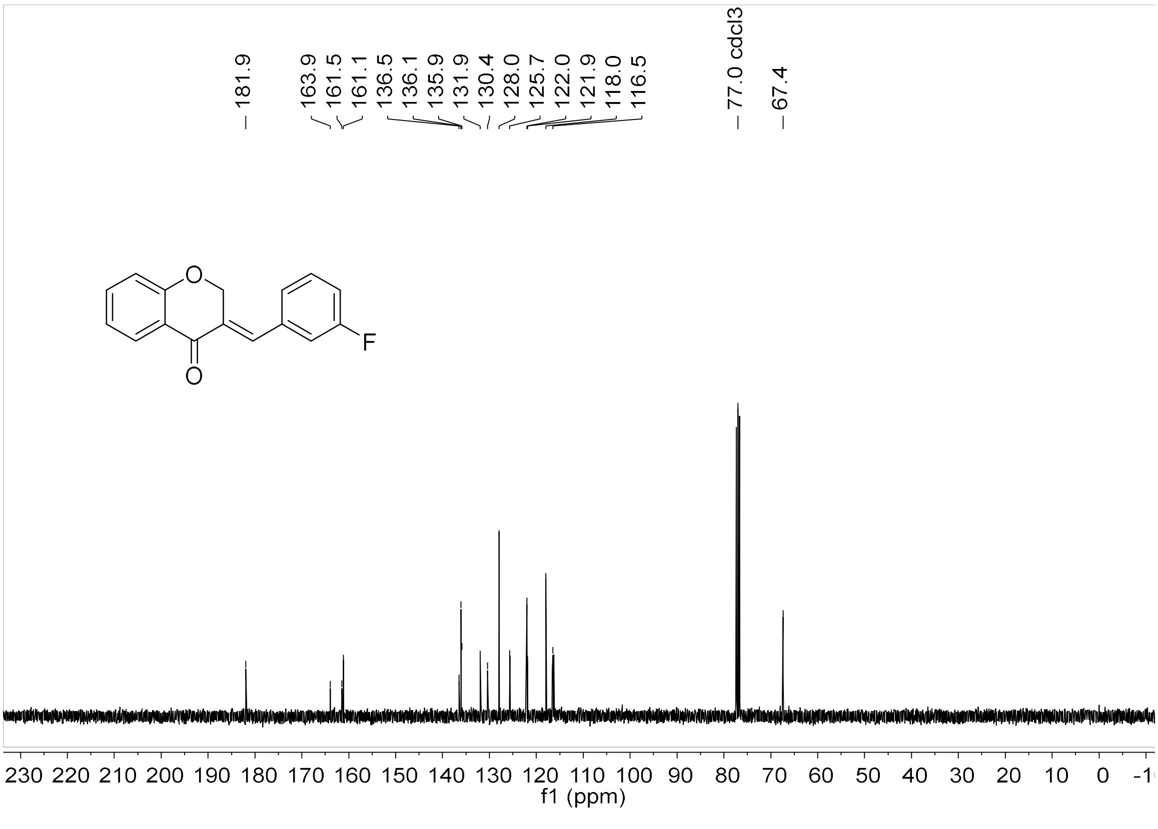

^{1}H NMR (400 MHz, $CDCl_3$) spectrum of 7

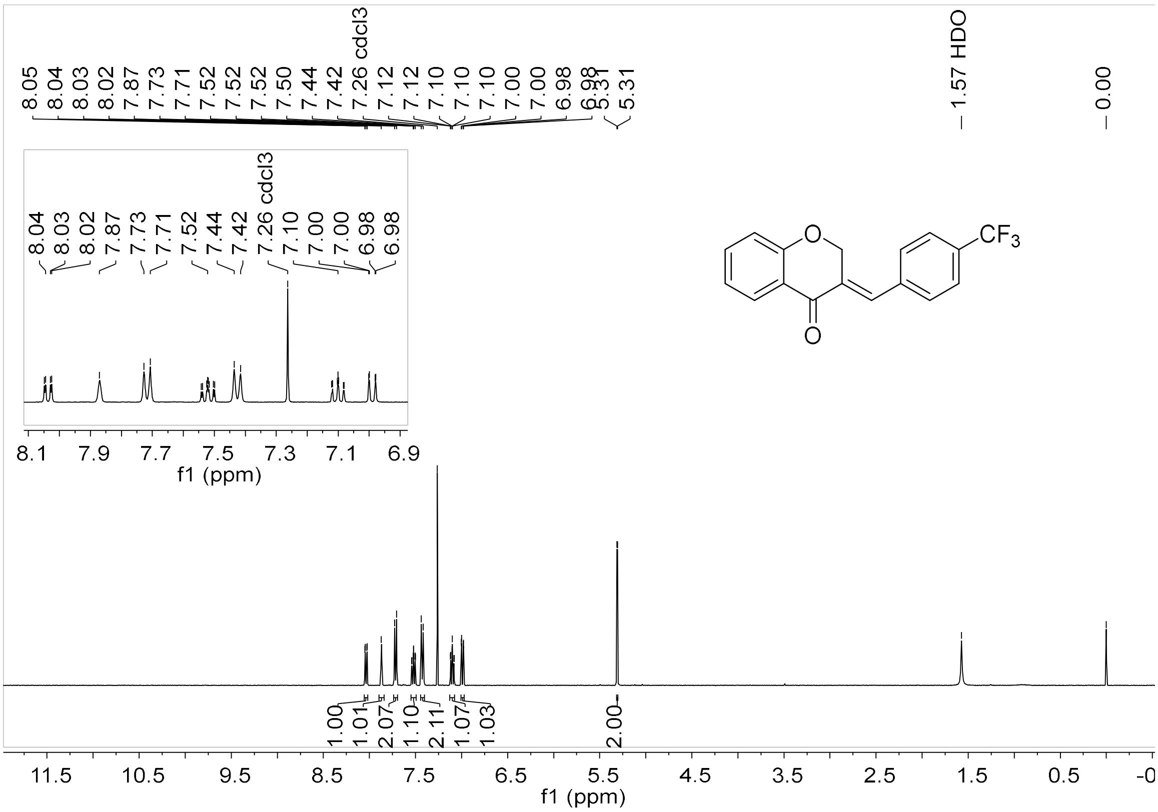

^{13}C NMR (101 MHz, $CDCl_3$) of 7

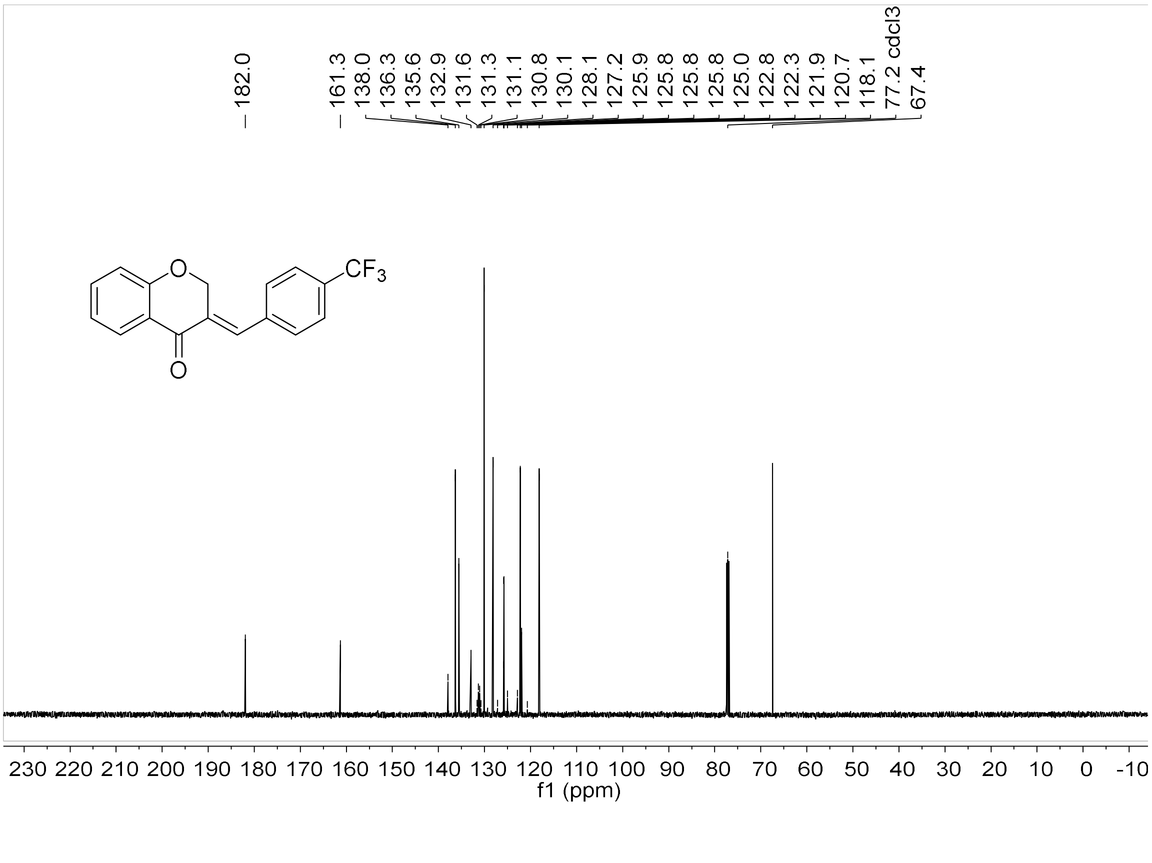

^{1}H NMR (400 MHz, $CDCl_3$) spectrum of 8

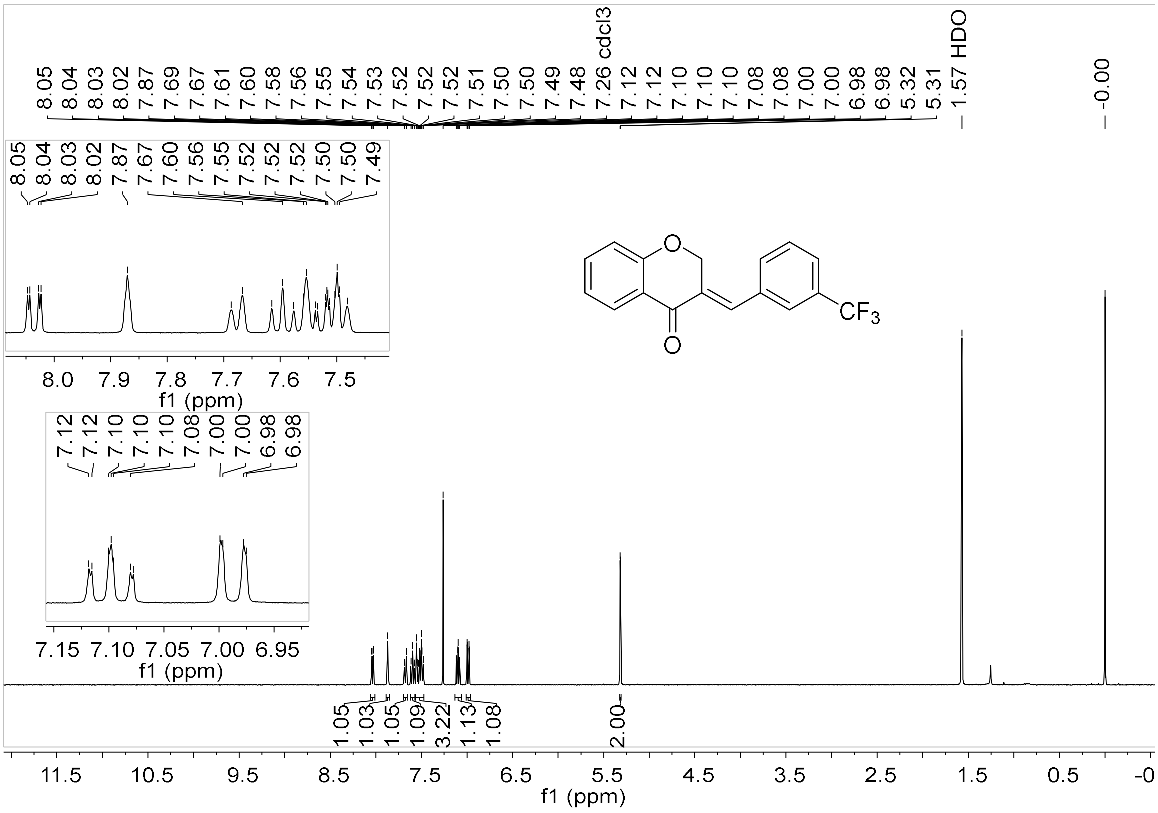

^{13}C NMR (101 MHz, $CDCl_3$) of 8

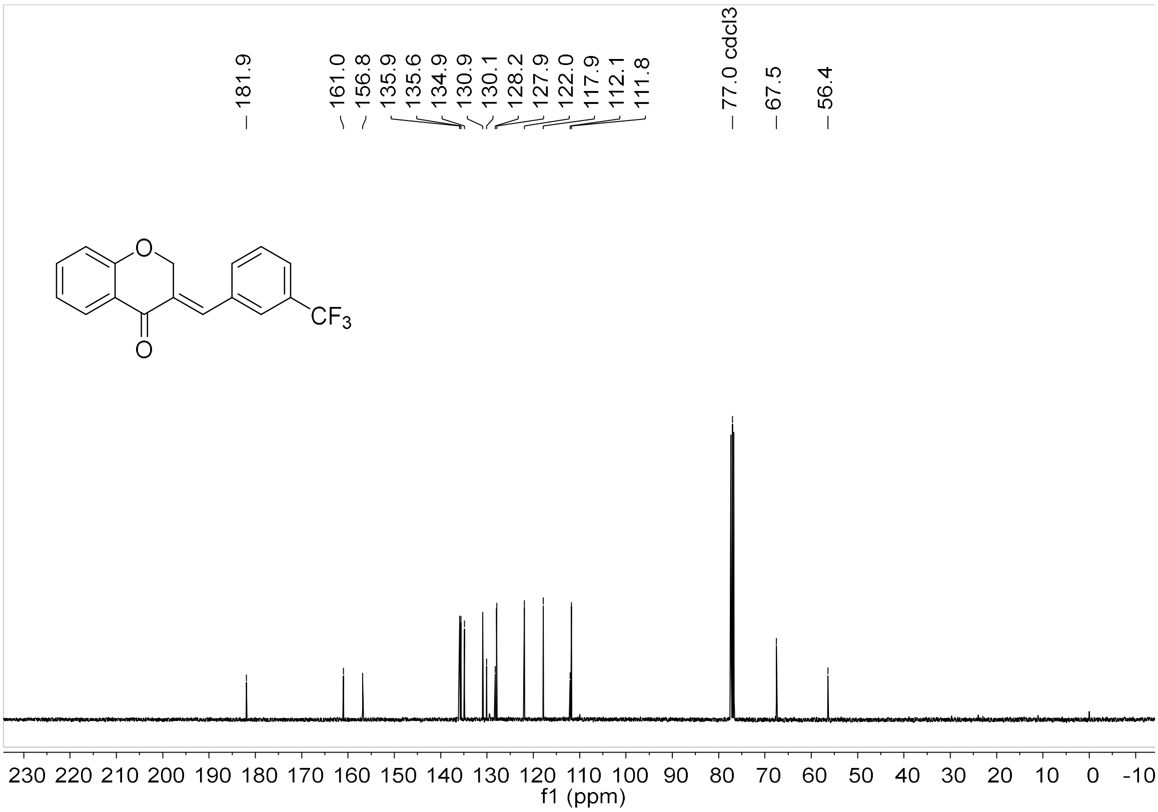

^{1}H NMR (400 MHz, $CDCl_3$) spectrum of 9

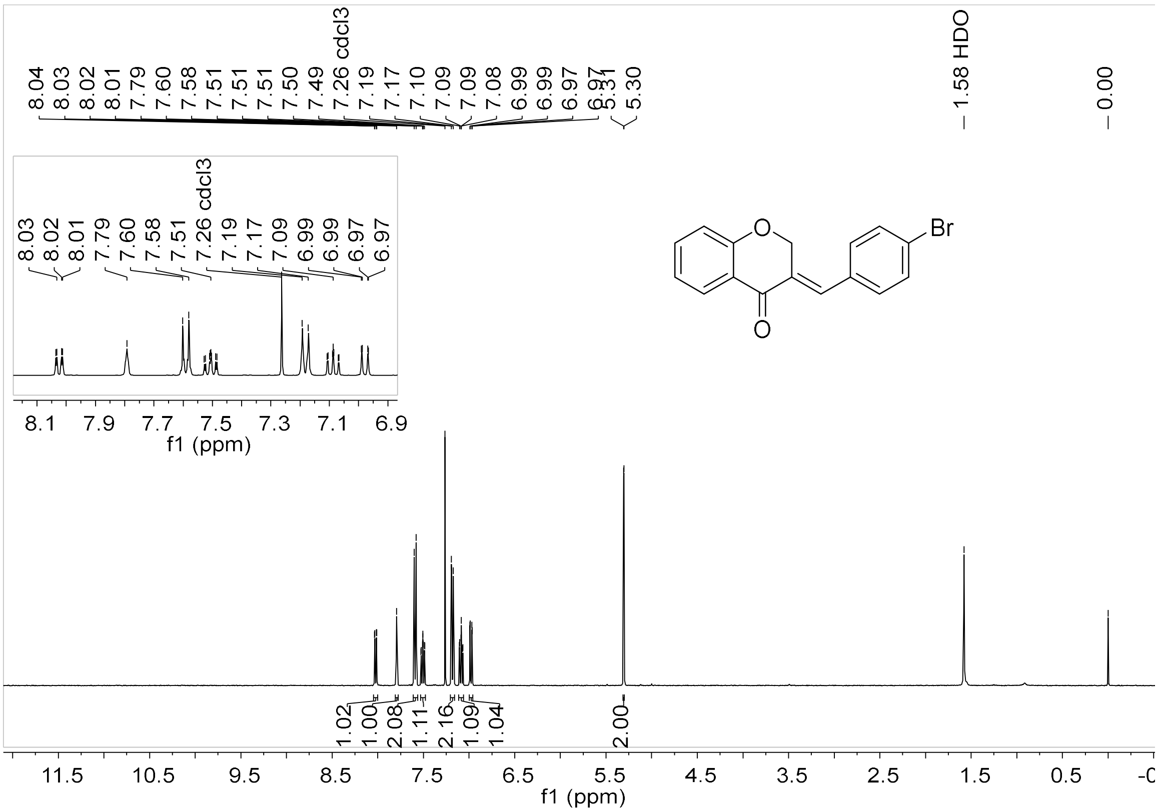

^{13}C NMR (101 MHz, $CDCl_3$) of 9

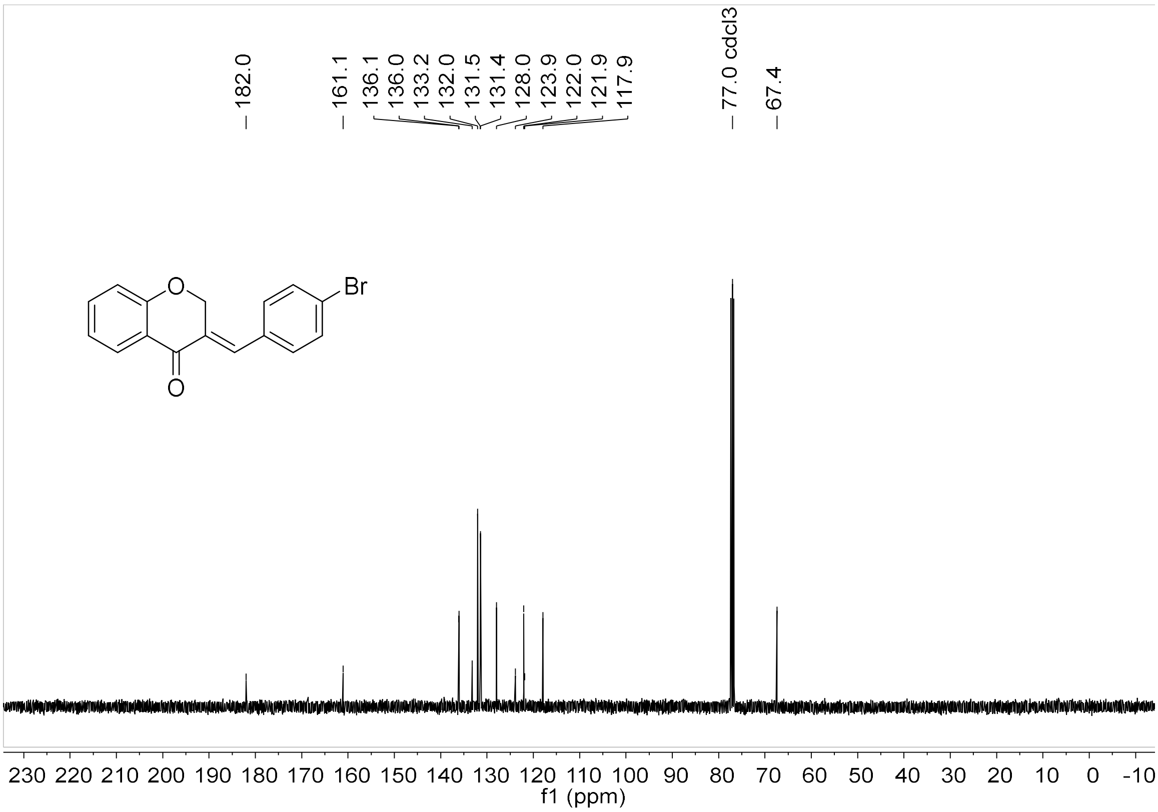

^{1}H NMR (500 MHz, $CDCl_3$) spectrum of 10

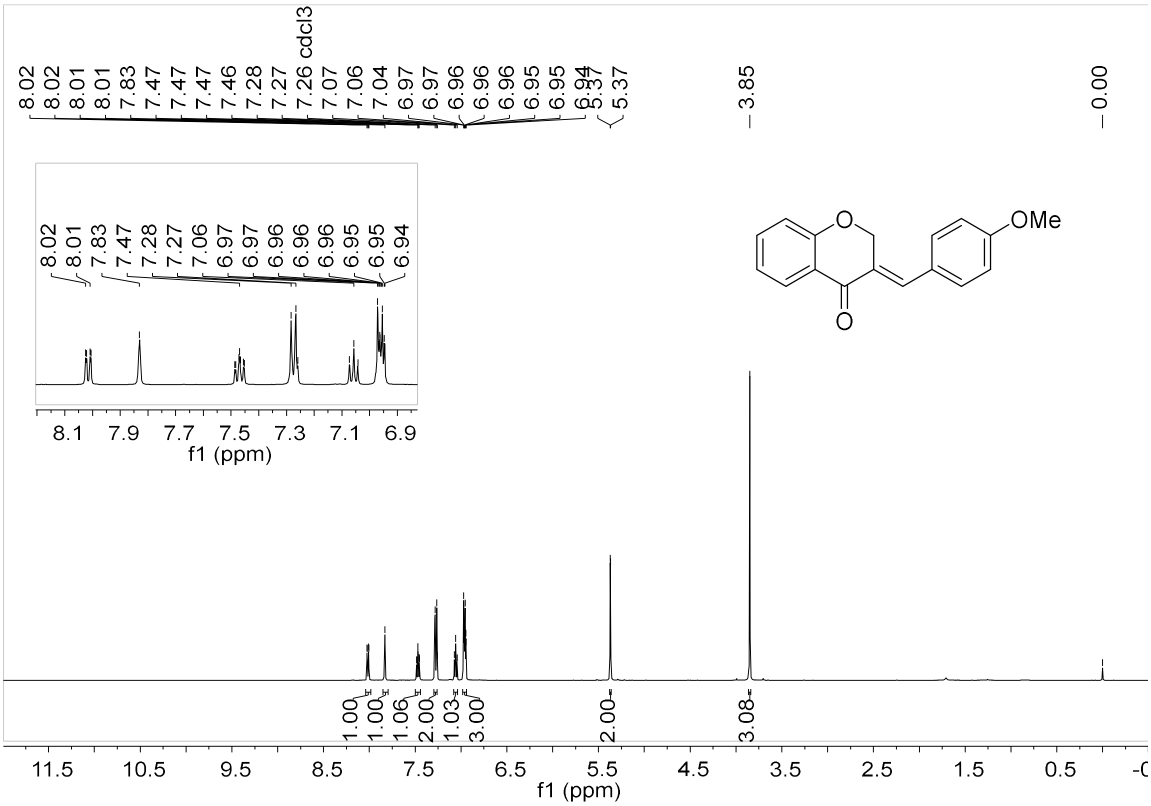

^{13}C NMR (126 MHz, $CDCl_3$) of 10

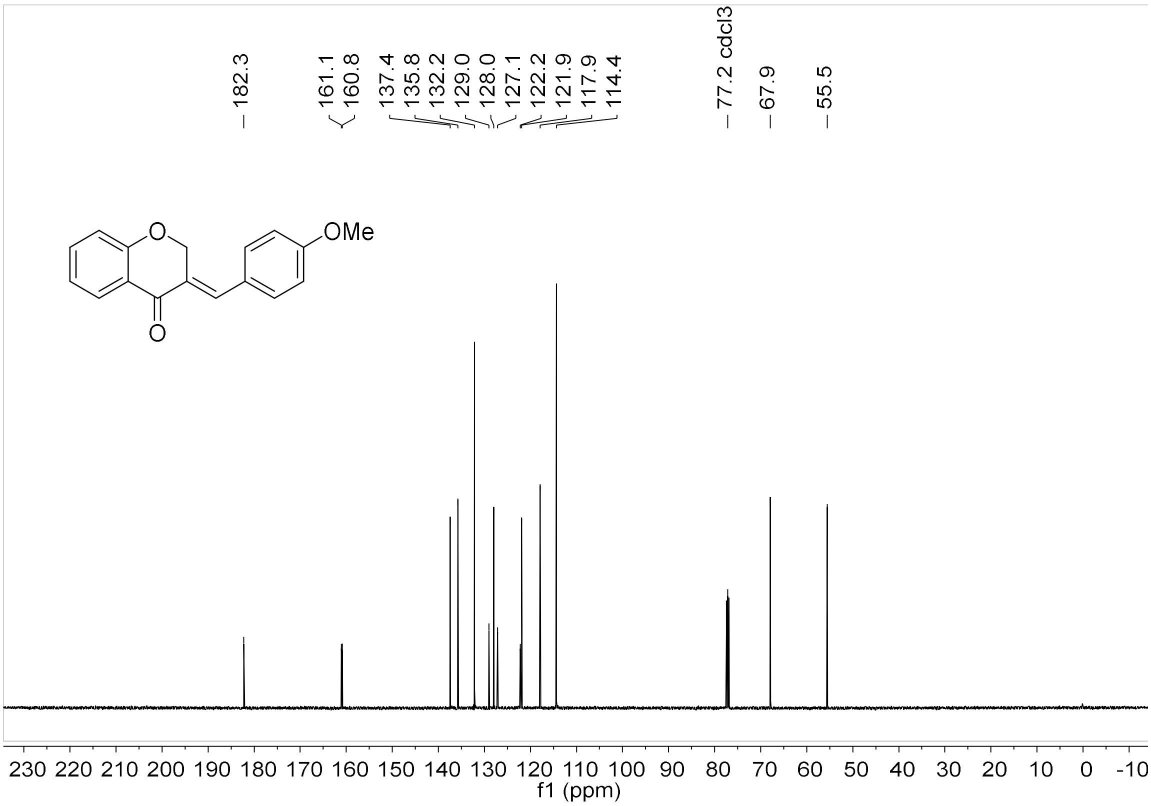

^{1}H NMR (400 MHz, DMSO-d_6) spectrum of 11

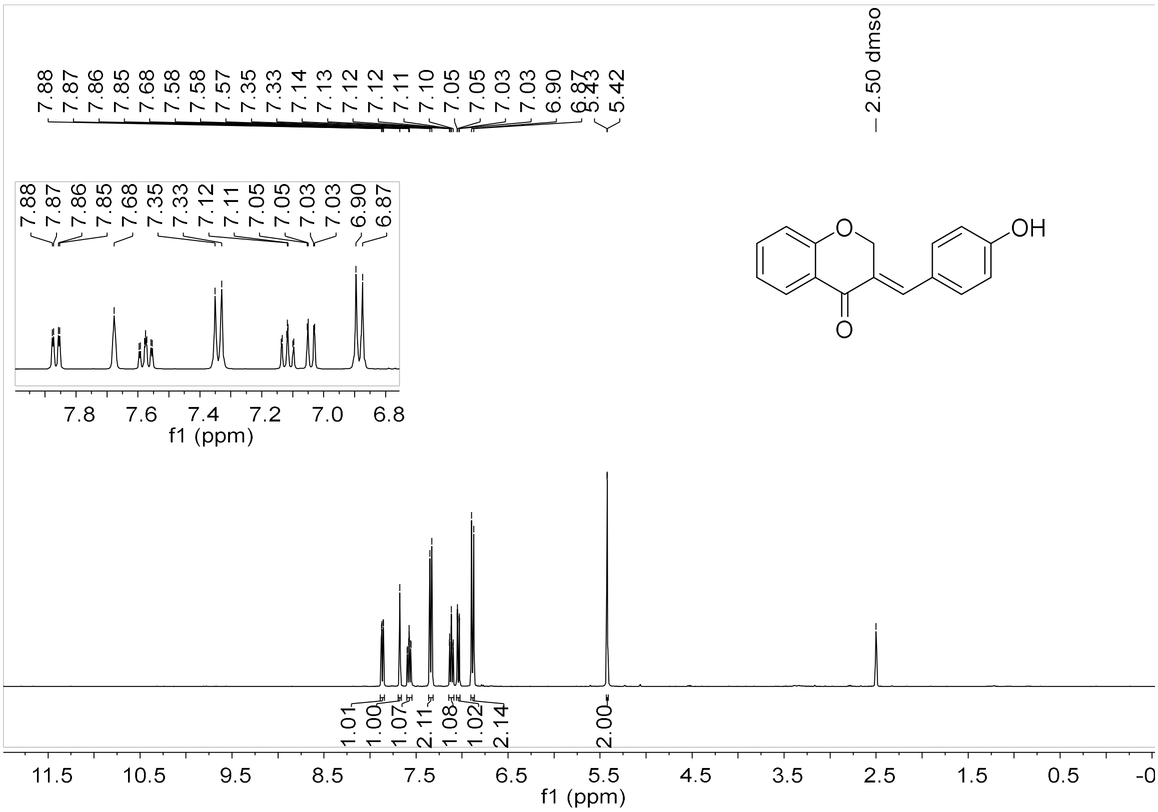

^{13}C NMR (101 MHz, DMSO-d_6) of 11

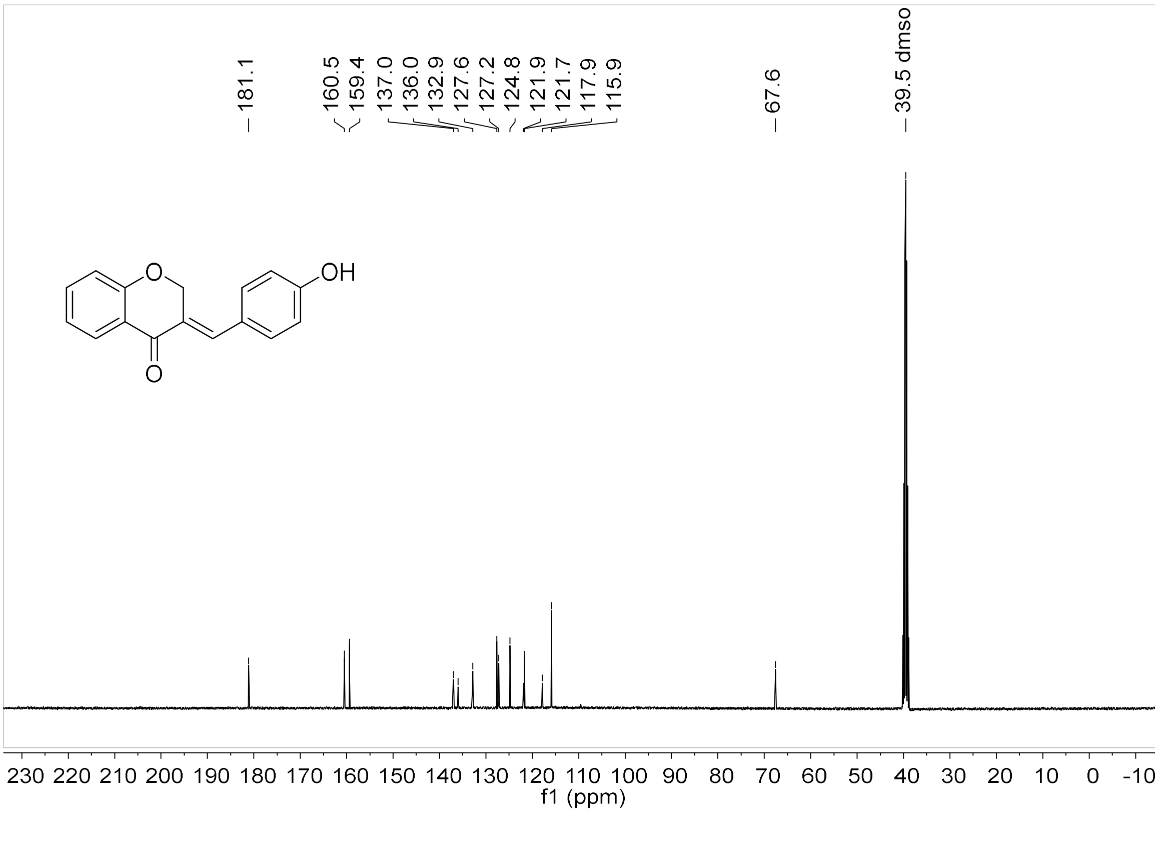

^{1}H NMR (400 MHz, DMSO-d_6) spectrum of 12

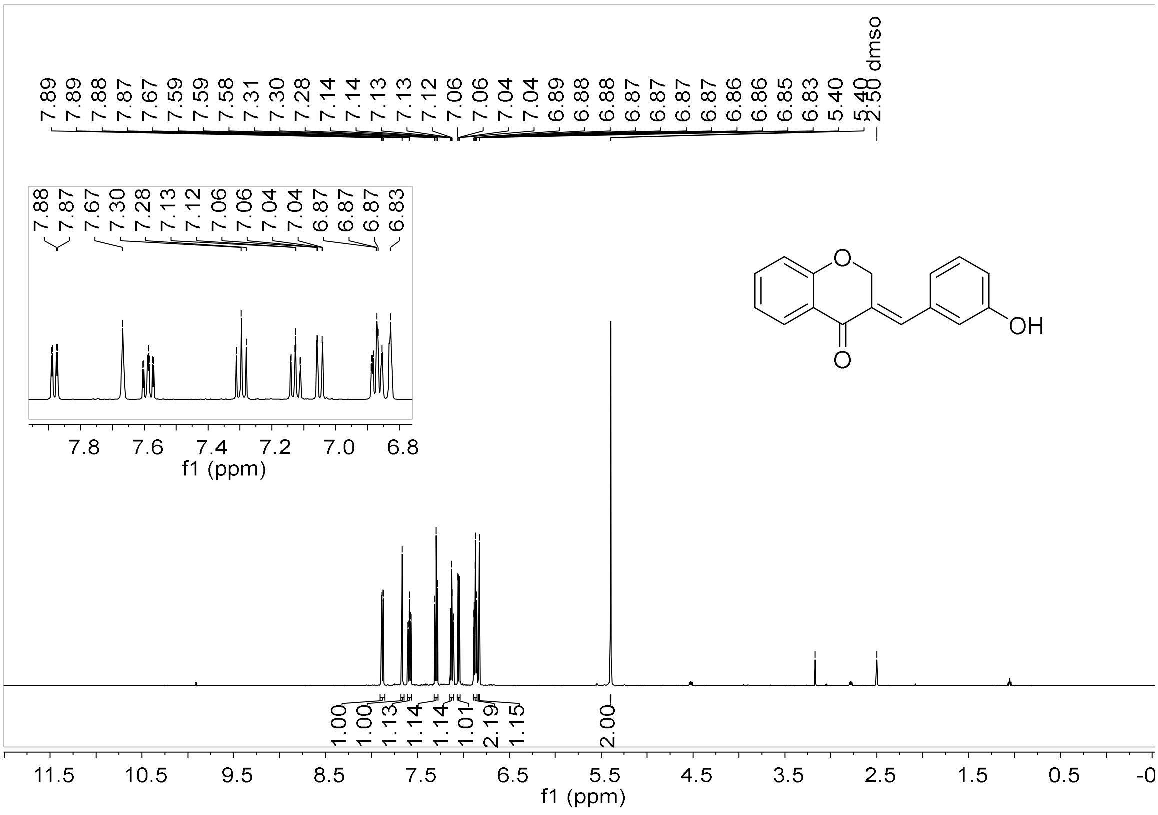

^{13}C NMR (101 MHz, DMSO-d_6) of 12

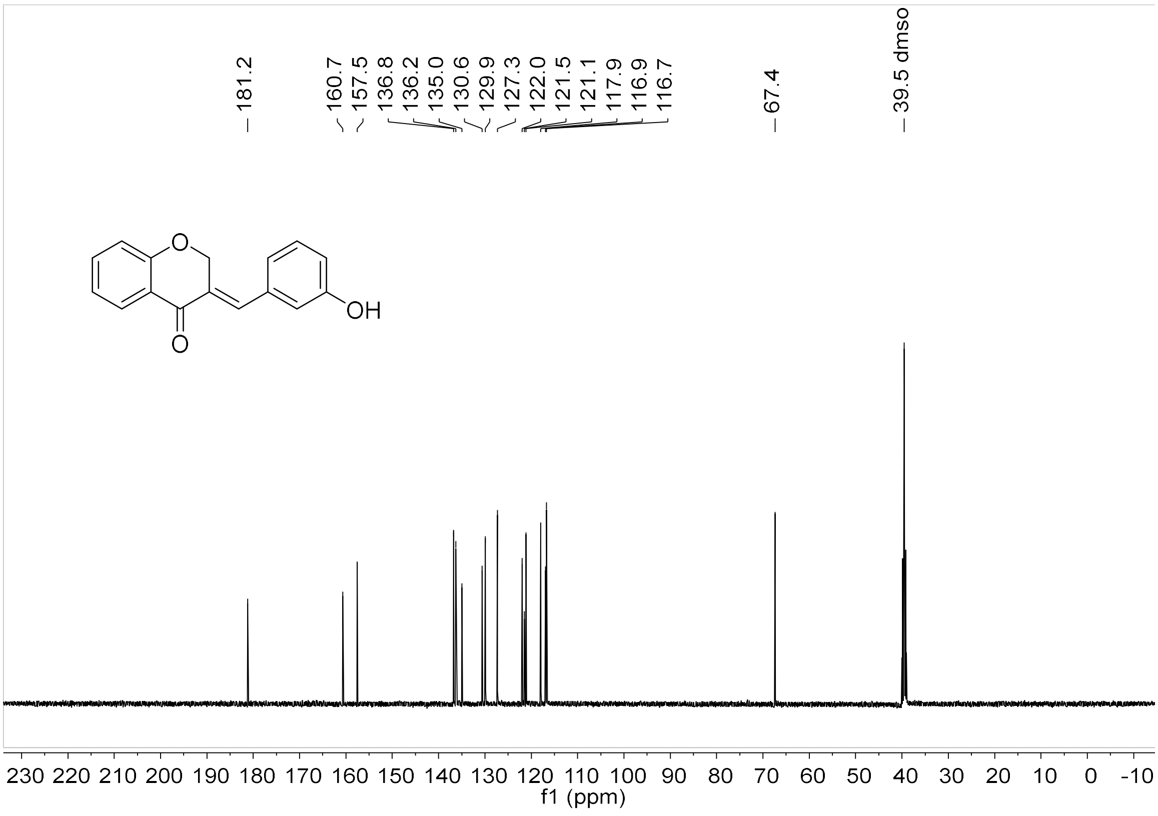

^{1}H NMR (400 MHz, CDCl$_3$) spectrum of 13

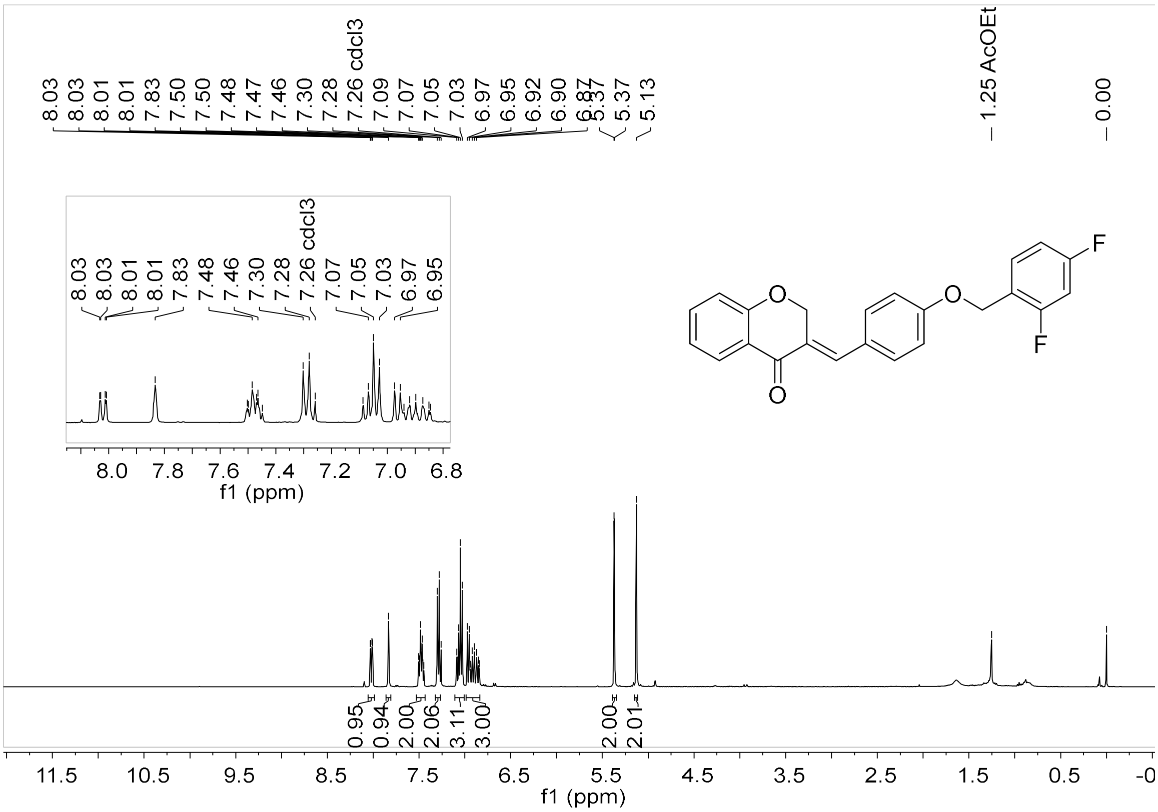

^{13}C NMR (101 MHz, CDCl$_3$) of 13

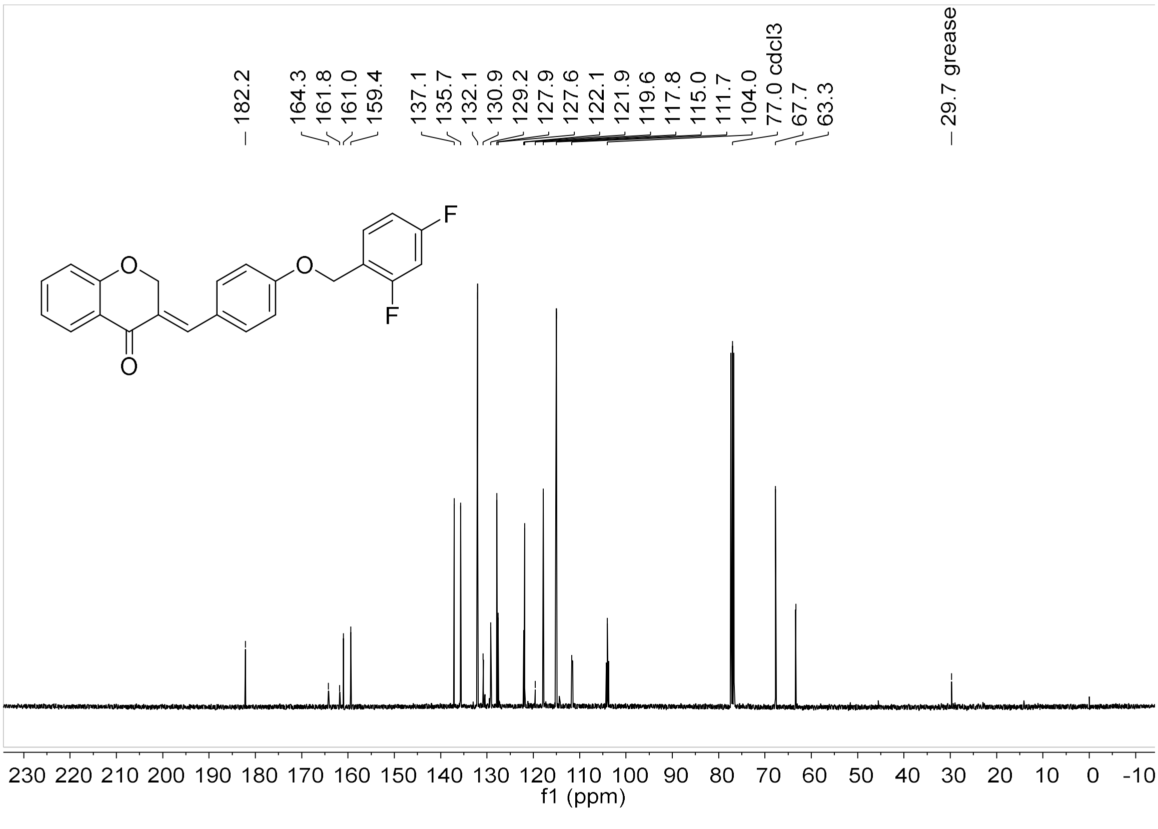

^{1}H NMR (500 MHz, $CDCl_3$) spectrum of 14

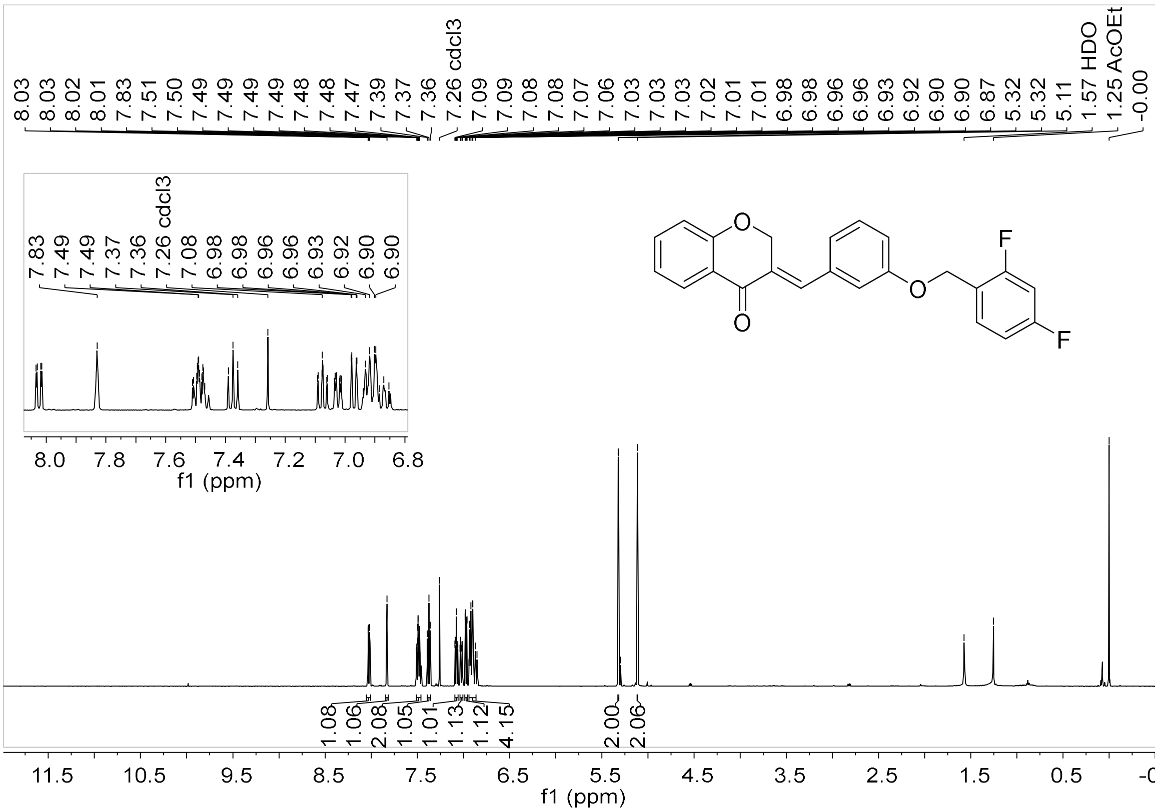

^{13}C NMR (126 MHz, $CDCl_3$) of 14

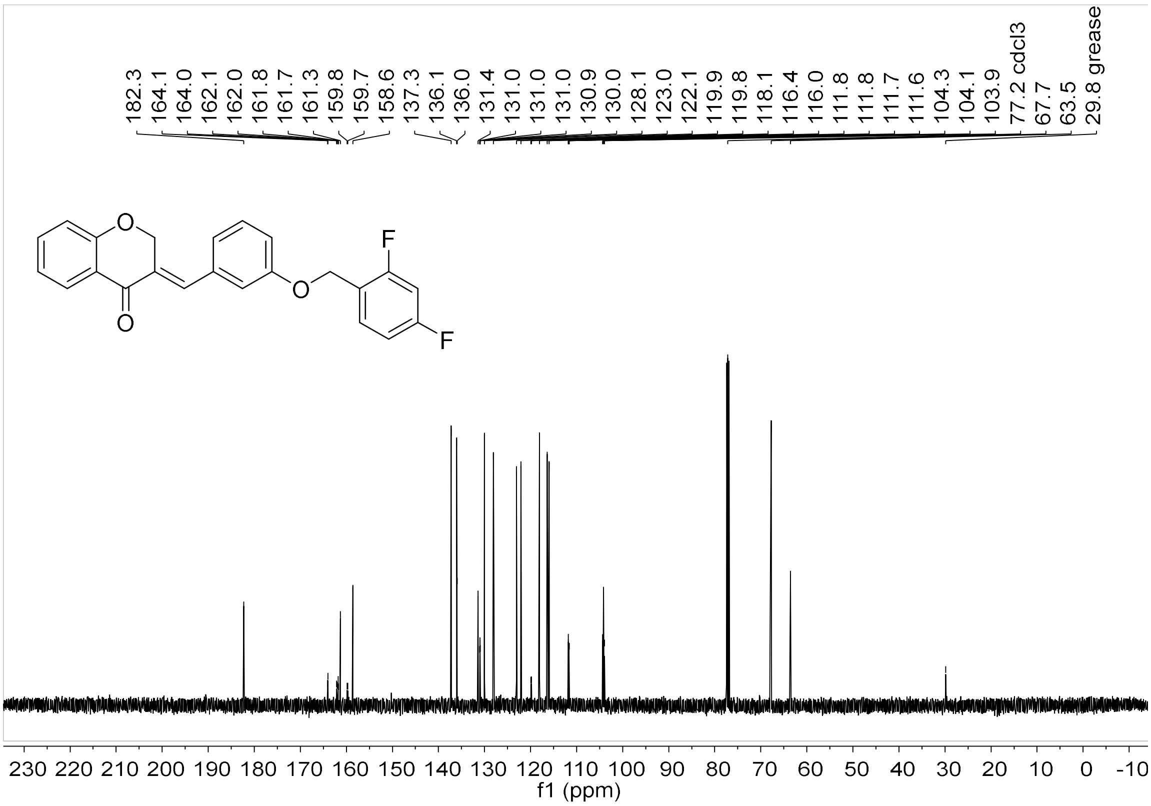

^{1}H NMR (500 MHz, CDCl$_3$) spectrum of 15

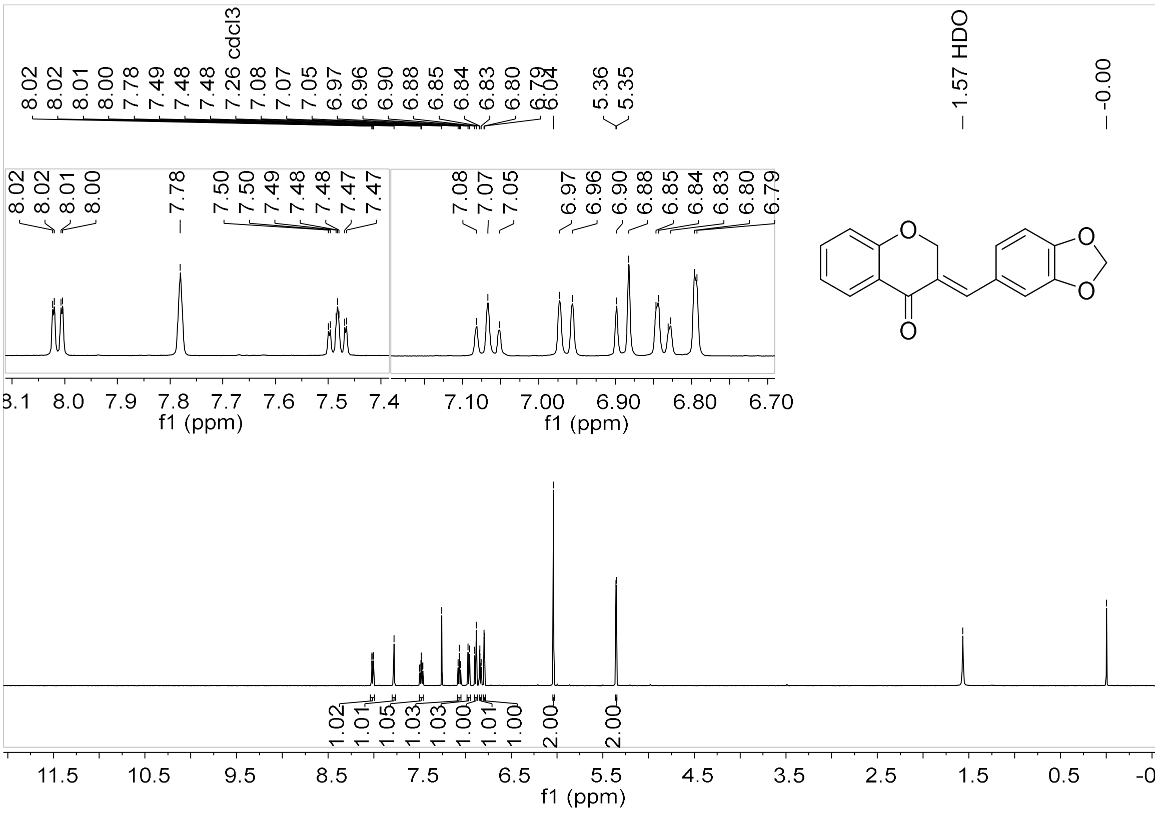

^{13}C NMR (101 MHz, CDCl$_3$) of 15

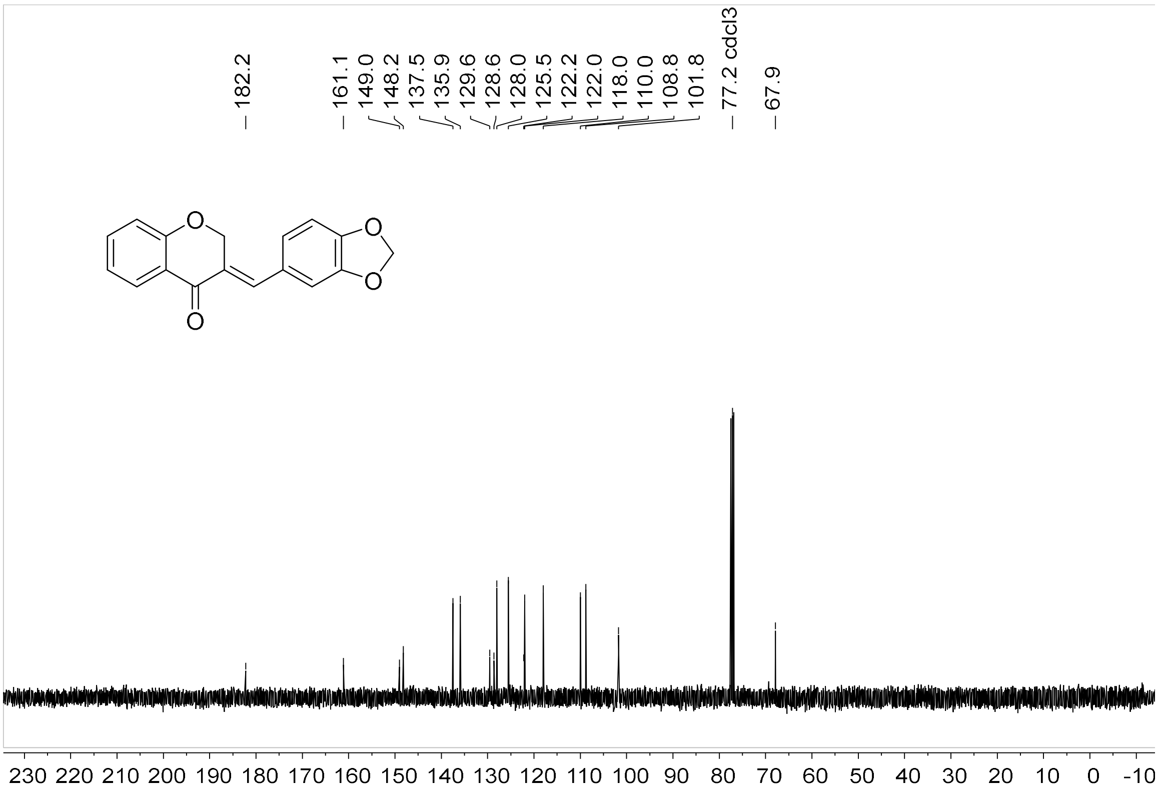

^{1}H NMR (400 MHz, DMSO-d_6) spectrum of 16

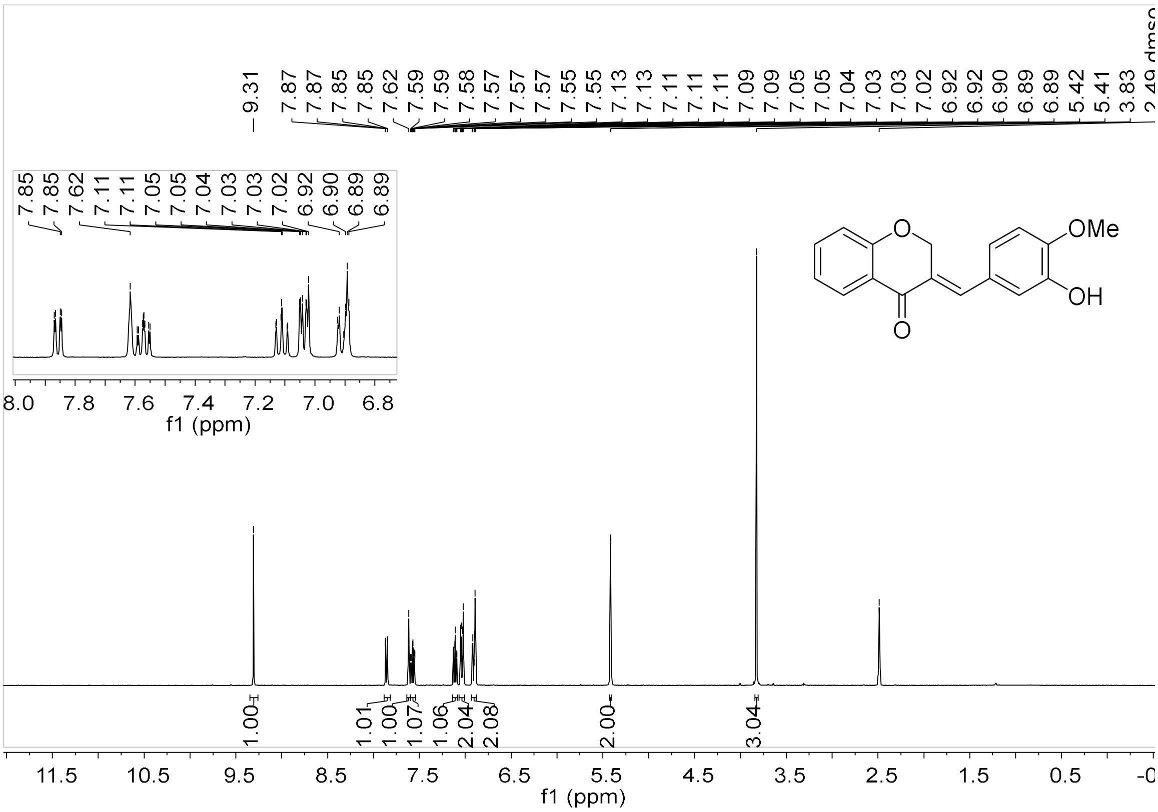

^{13}C NMR (126 MHz, $CDCl_3$) of 16

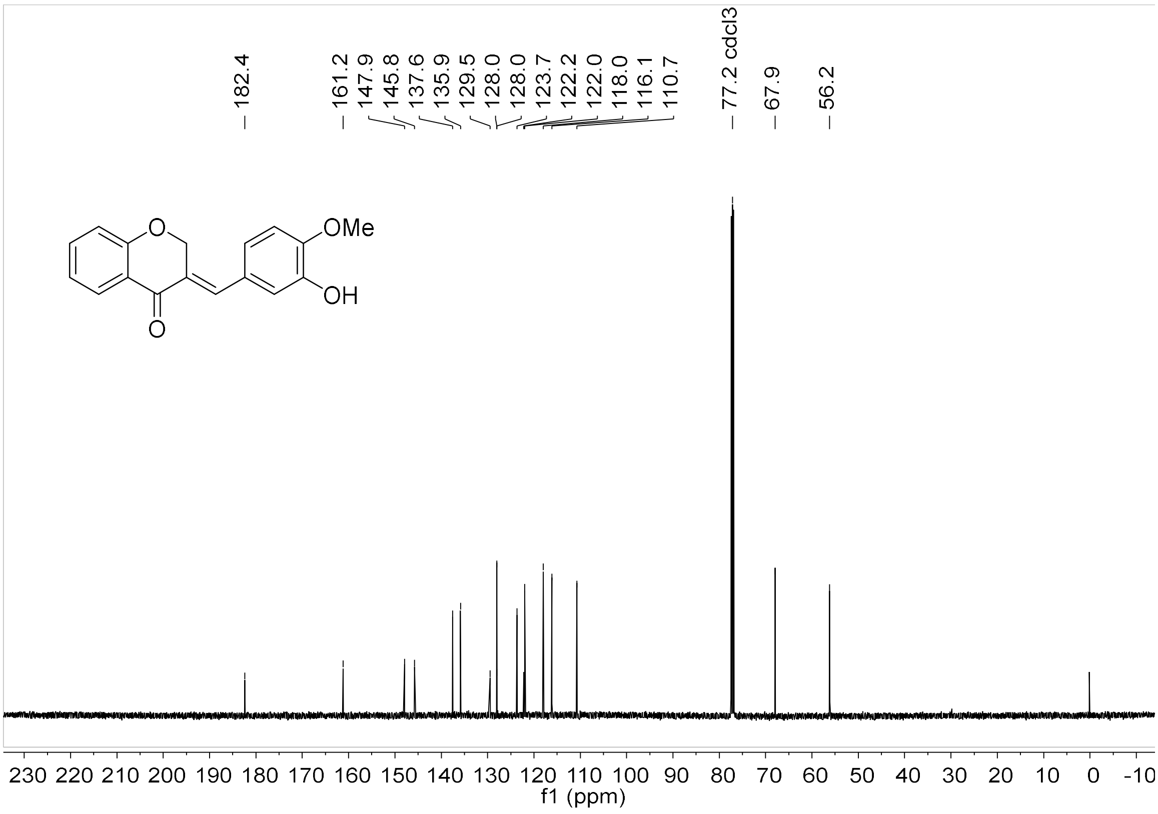

^{1}H NMR (400 MHz, CDCl₃) spectrum of 17

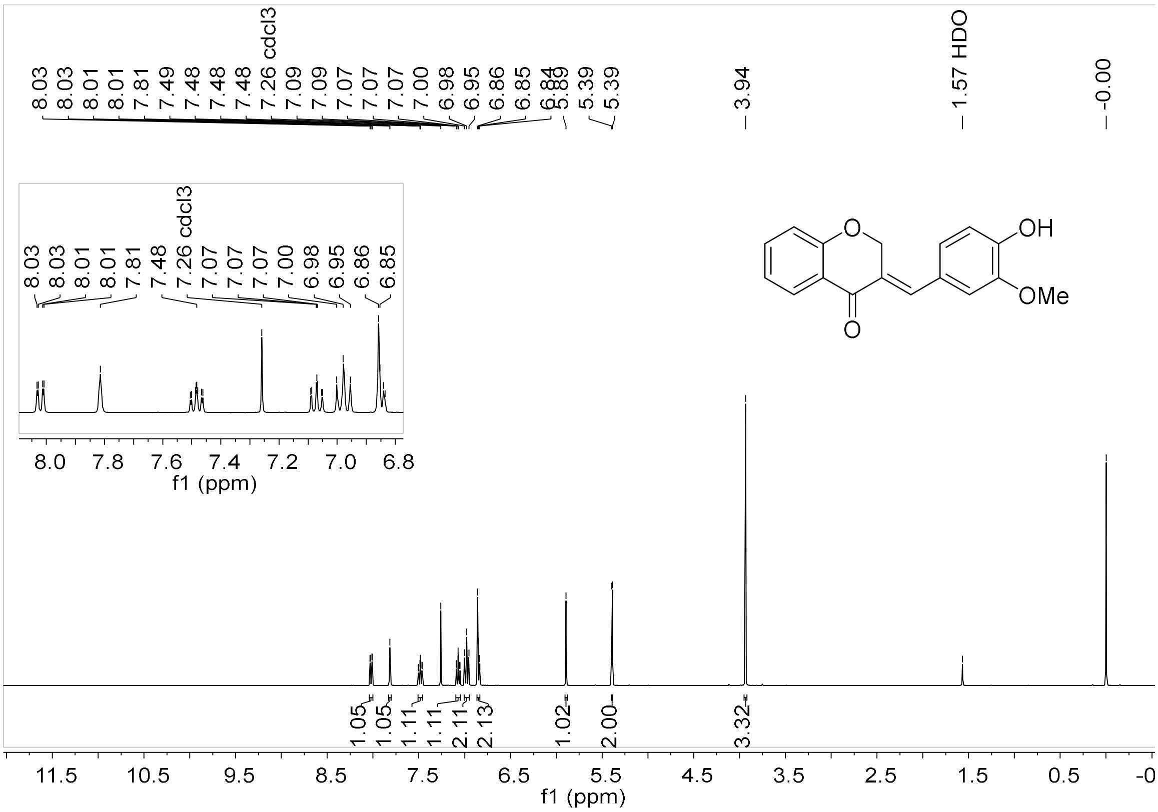

^{13}C NMR (126 MHz, CDCl₃) of 17

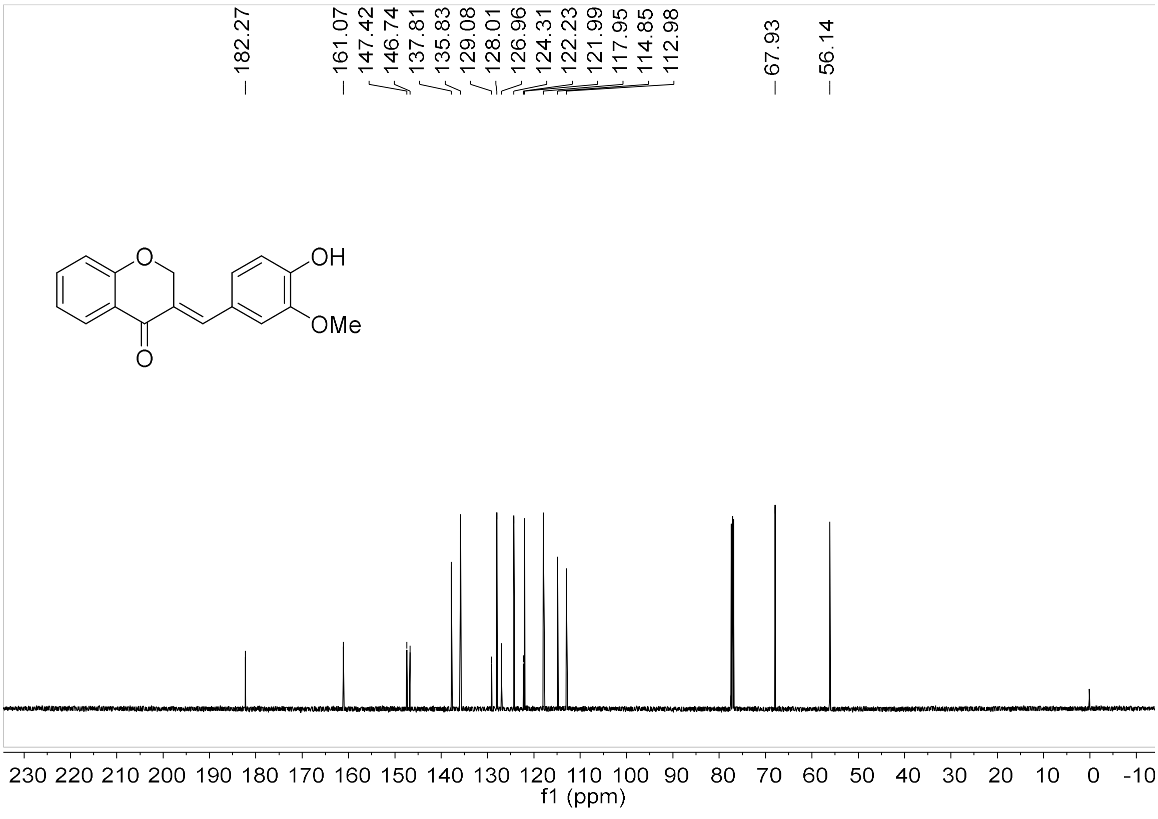

^{1}H NMR (400 MHz, $CDCl_3$) spectrum of 18

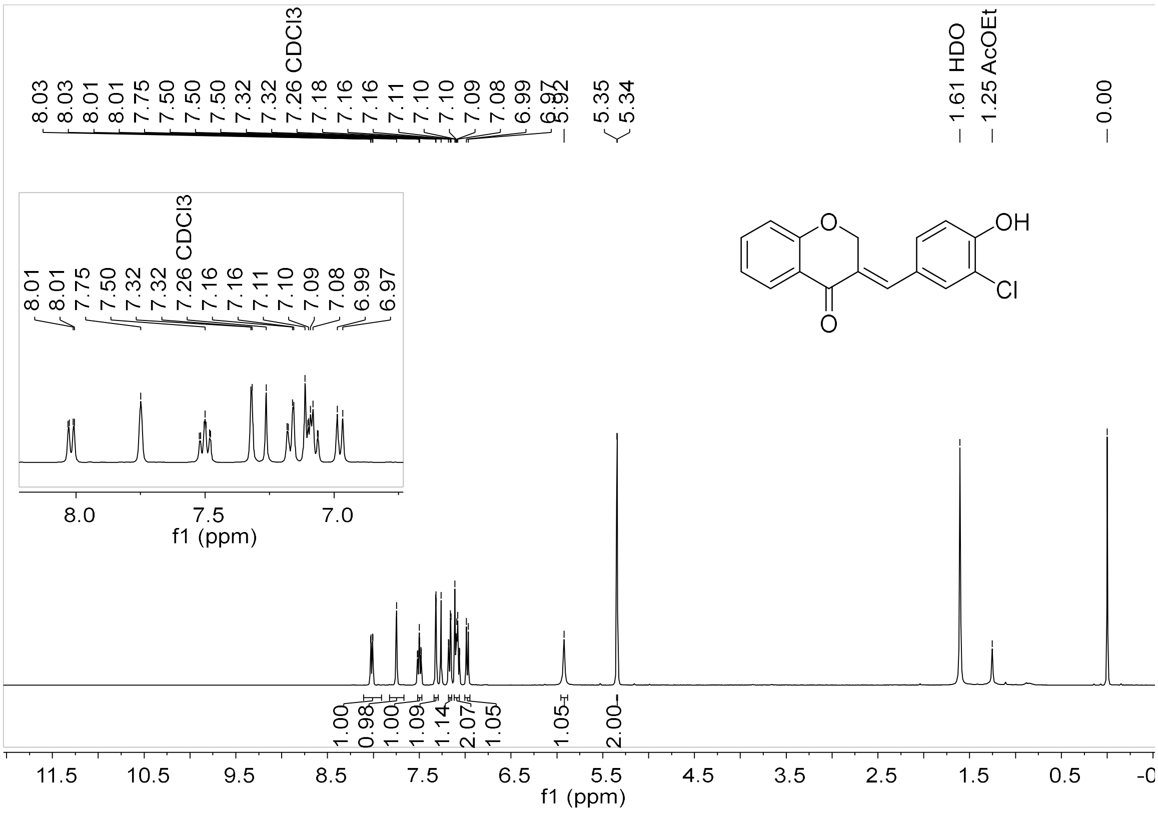

^{13}C NMR (101 MHz, DMSO-d_6) spectrum of 18

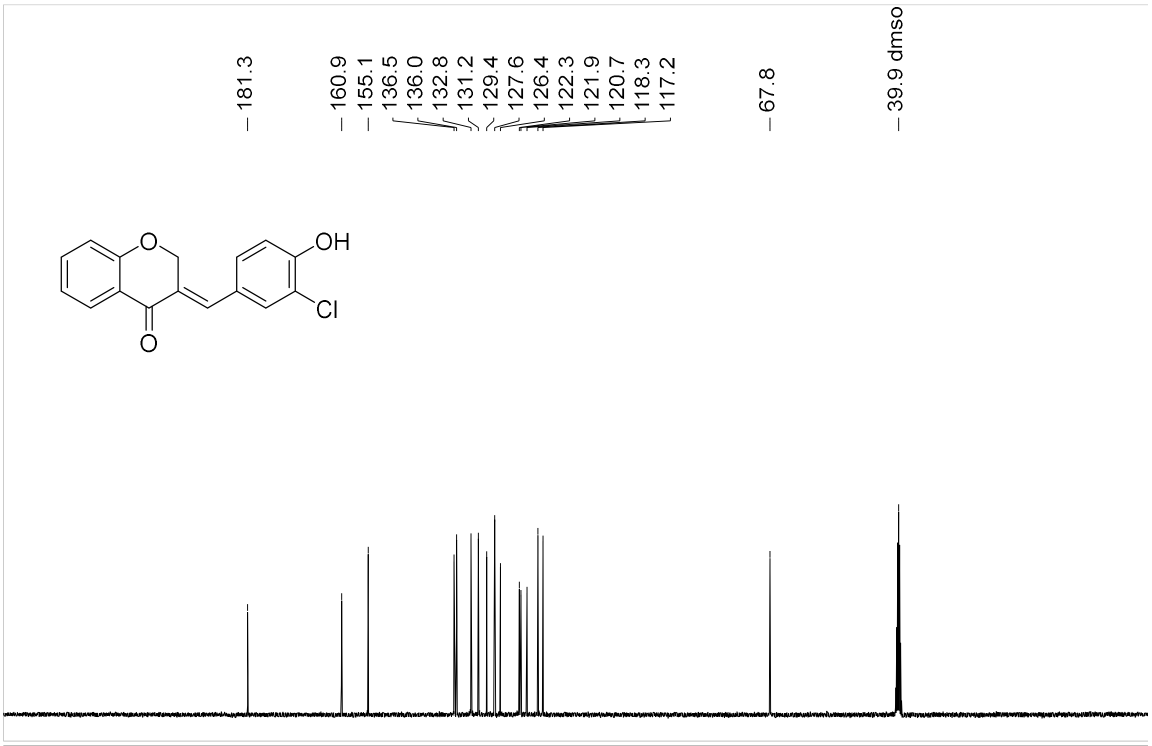

^{1}H NMR (400 MHz, CDCl$_3$) spectrum of 19

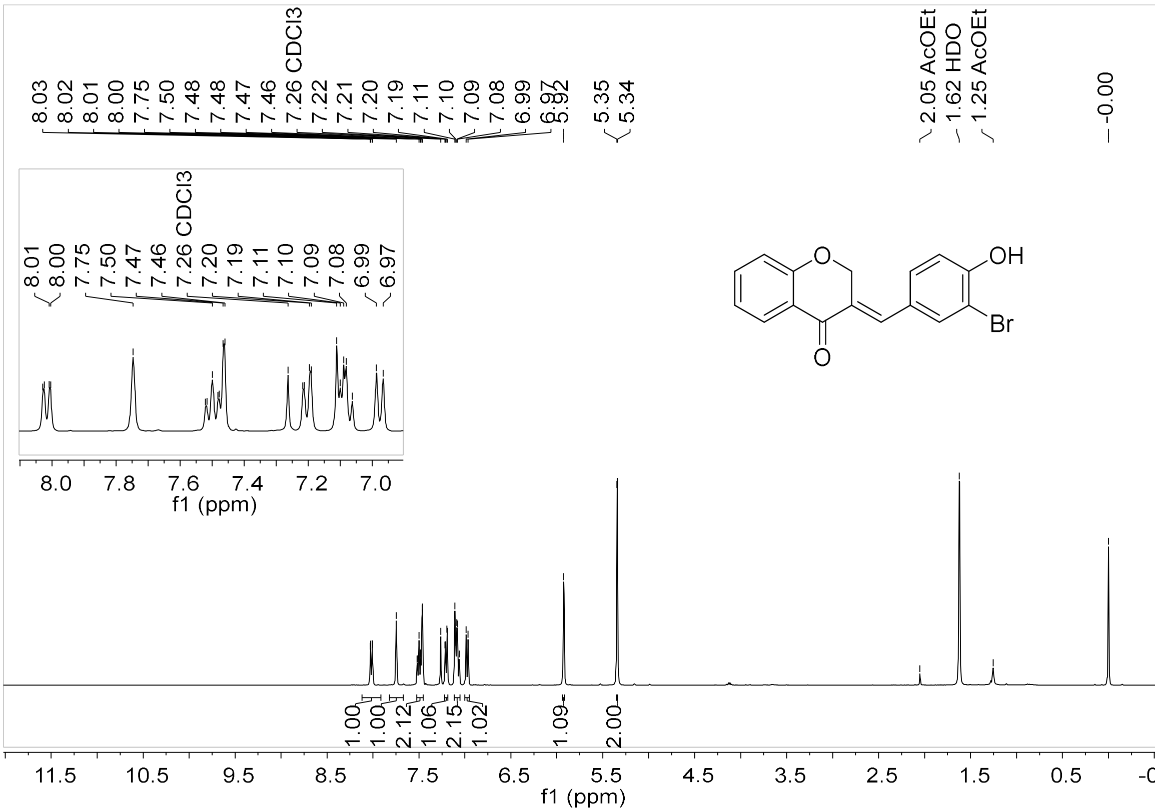

^{13}C NMR (126 MHz, CDCl$_3$) of 19

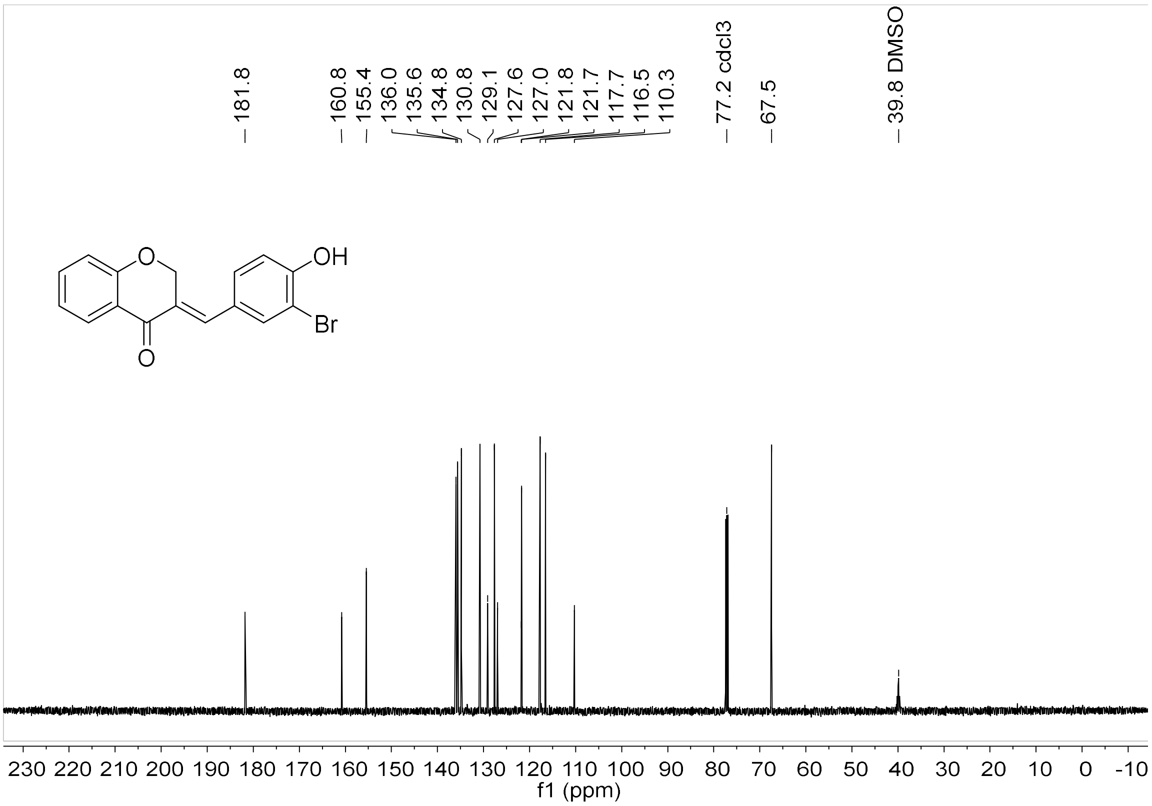

^{1}H NMR (500 MHz, CDCl$_3$) spectrum of 20

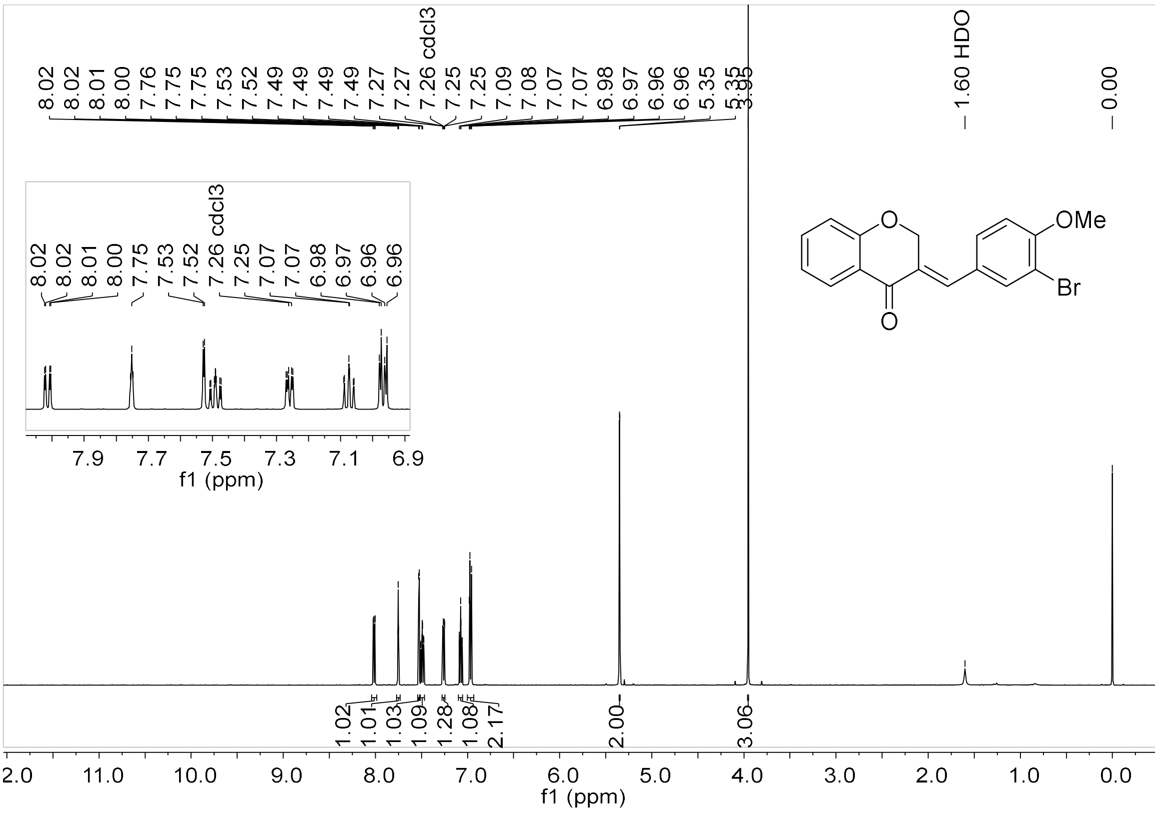

^{13}C NMR (126 MHz, CDCl$_3$) of 20

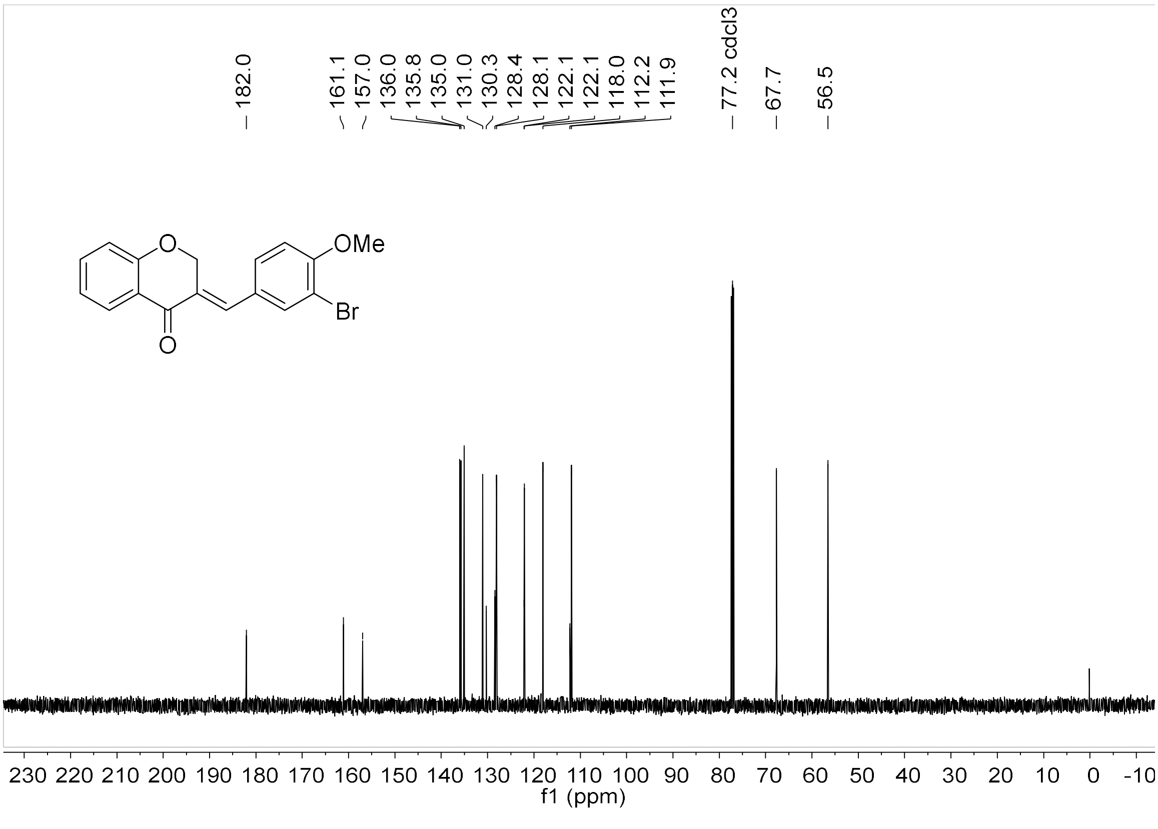

^{1}H NMR (500 MHz, DMSO-d_6) spectrum of 21

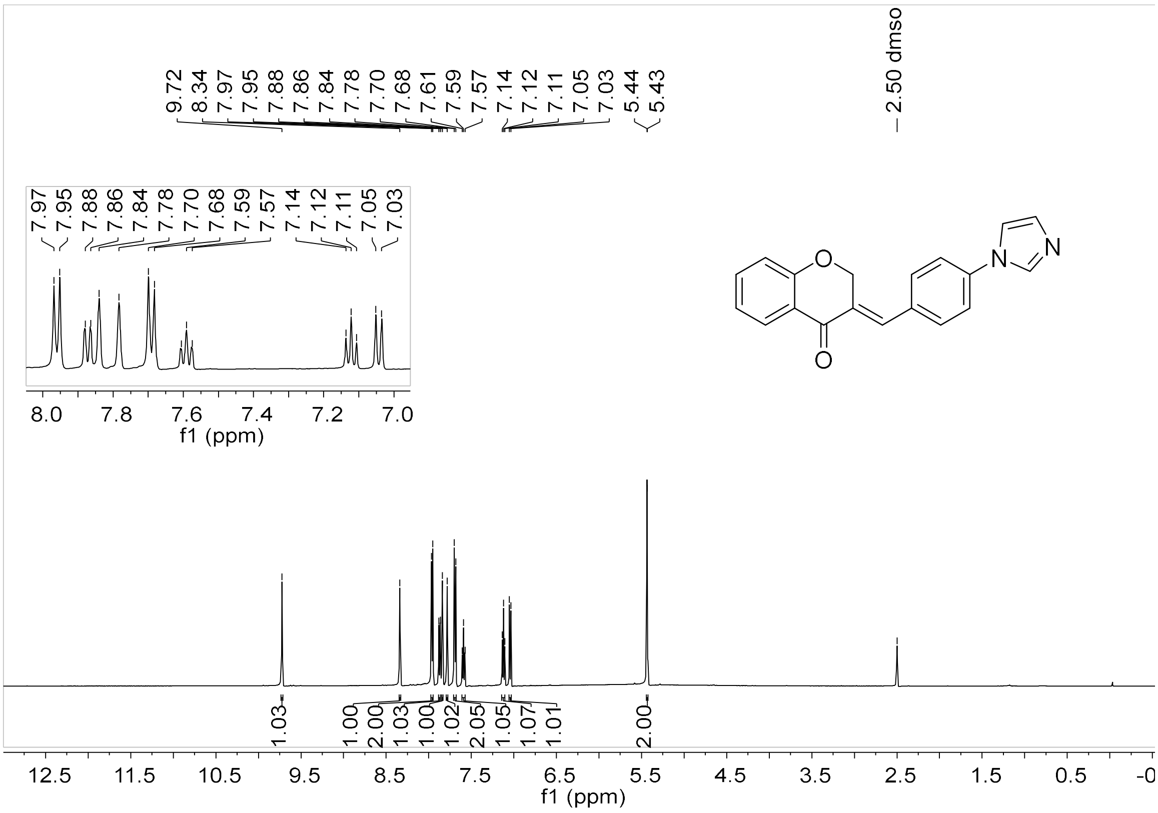

^{13}C NMR (126 MHz, DMSO-d_6) of 21

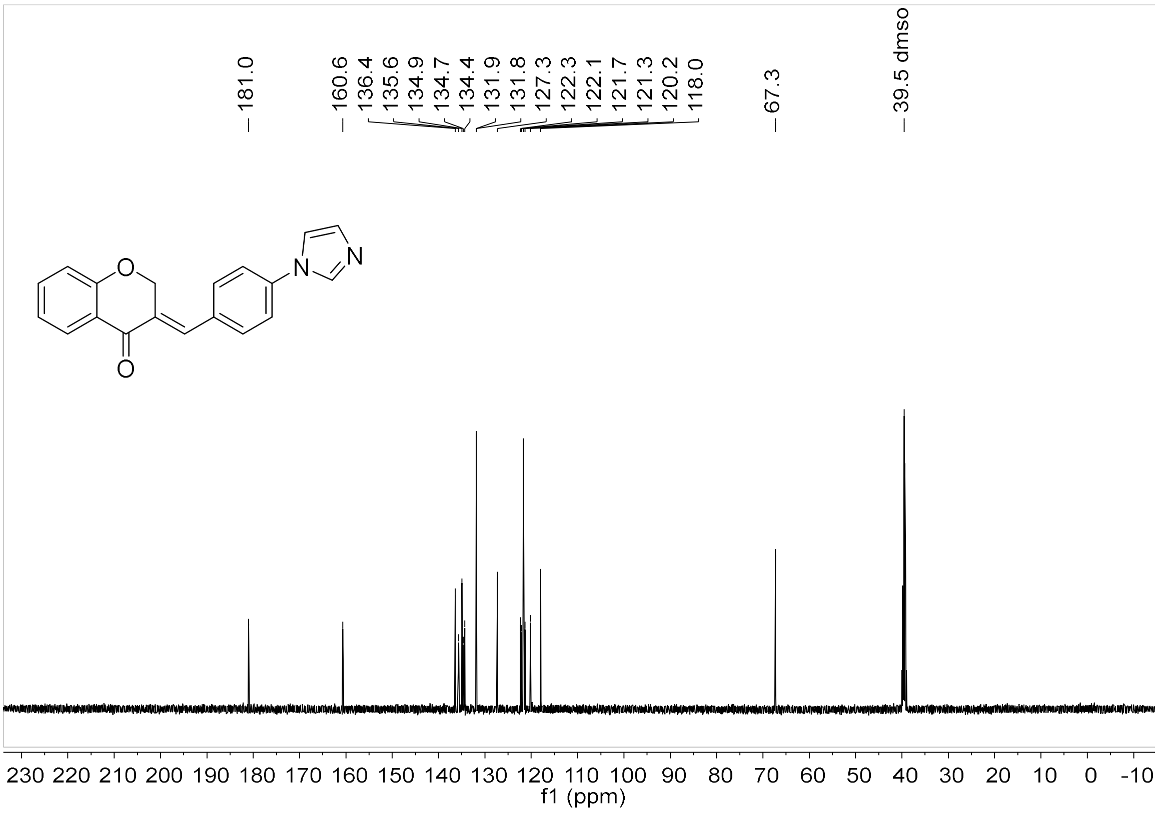

^{1}H NMR (500 MHz, CDCl$_3$) spectrum of 22

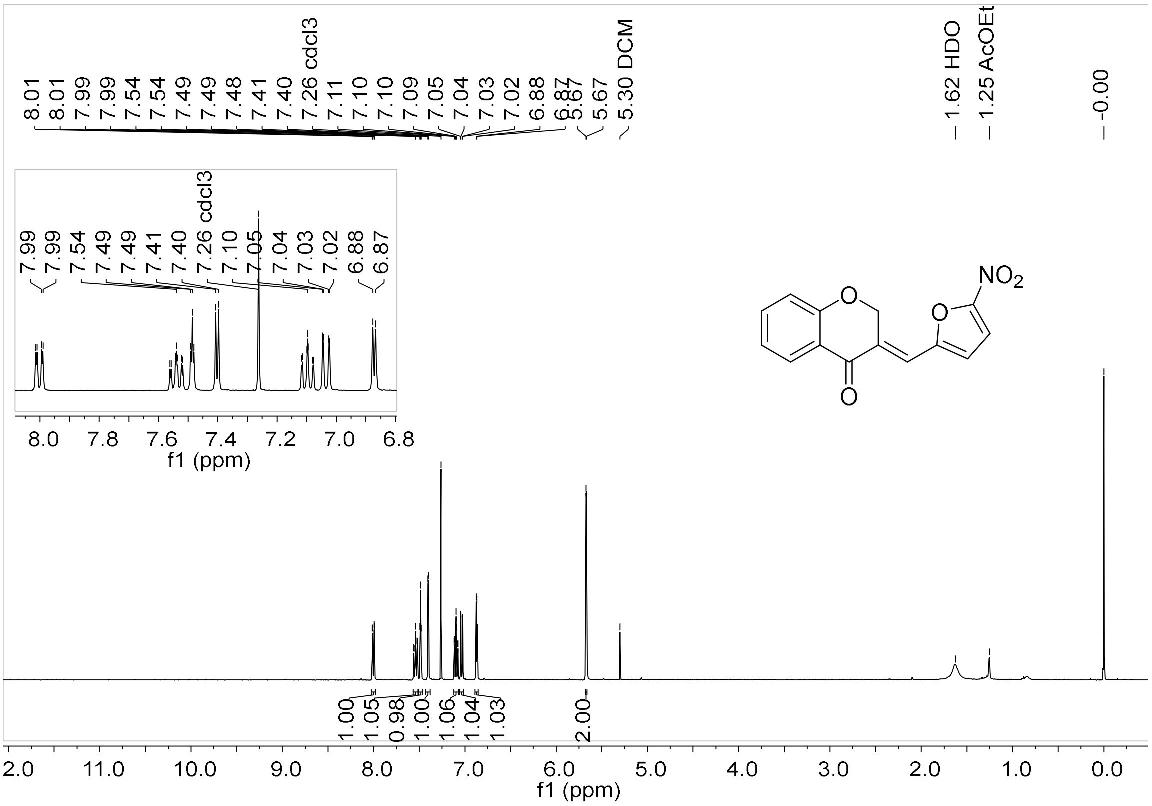

^{13}C NMR (126 MHz, CDCl$_3$) of 22

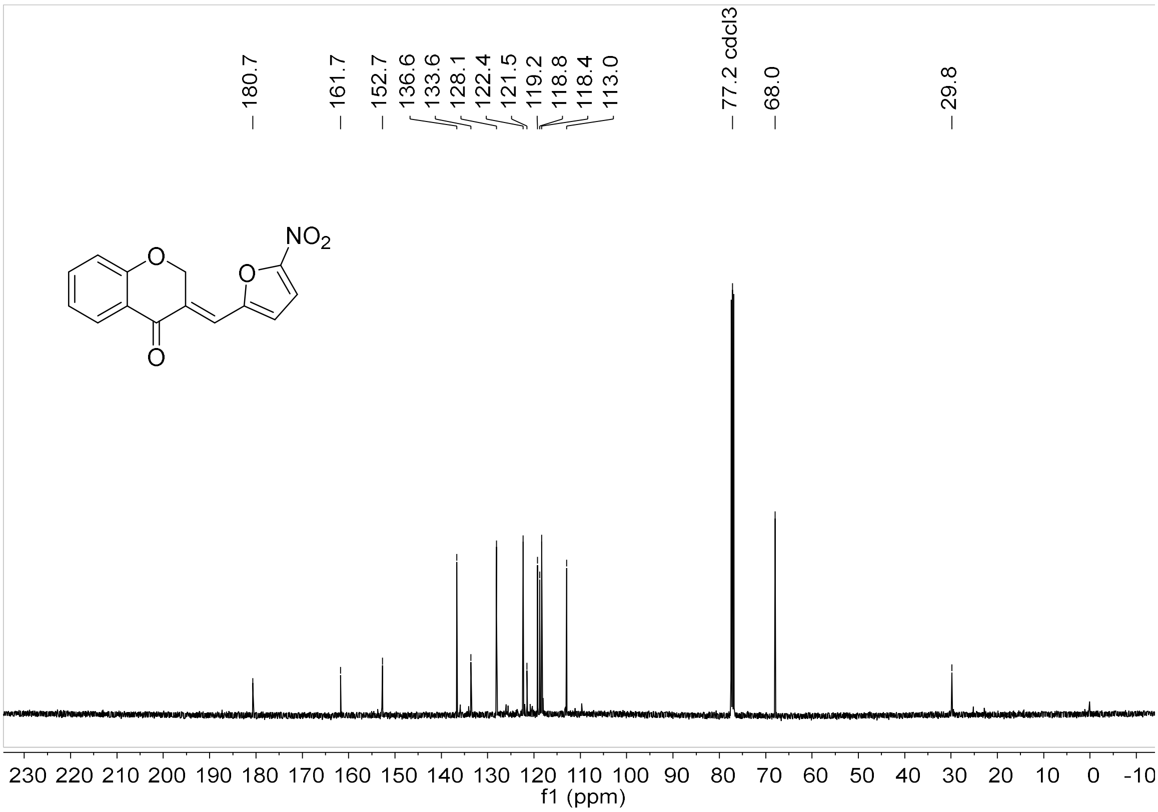

^{1}H NMR (500 MHz, DMSO-d_6) spectrum of 23

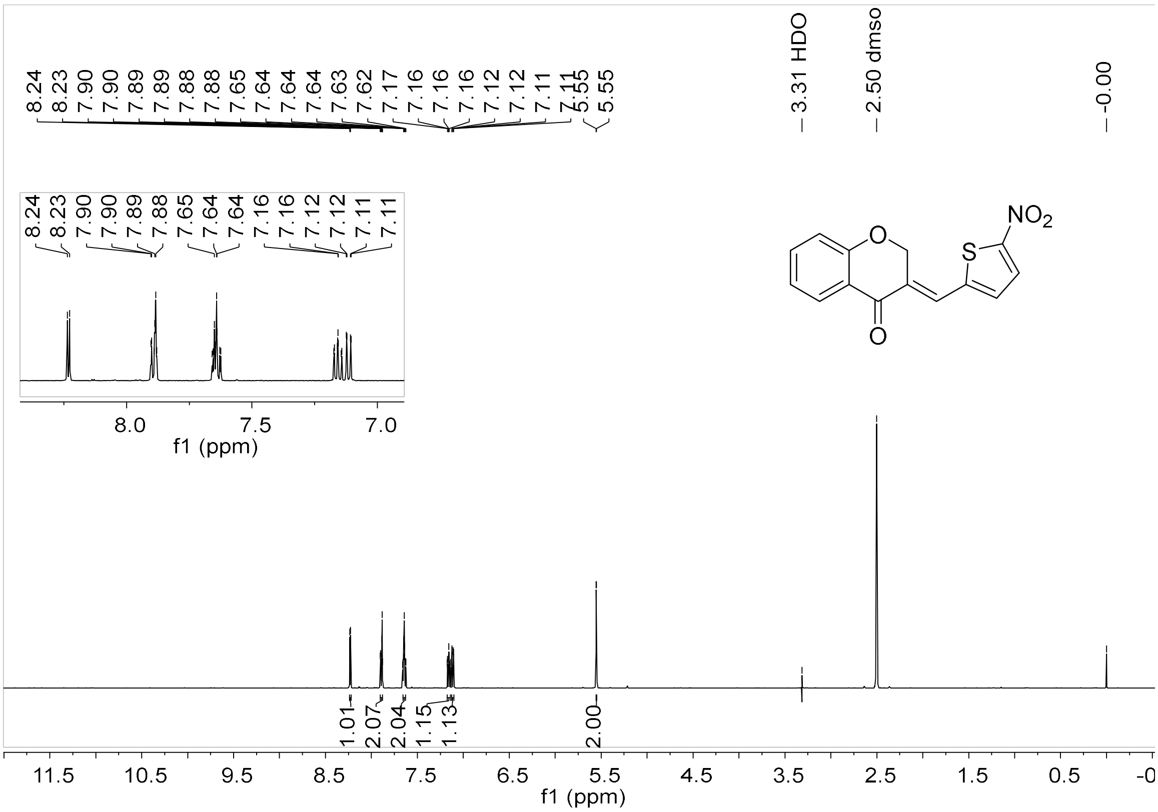

^{13}C NMR (126 MHz, DMSO-d_6) of 23

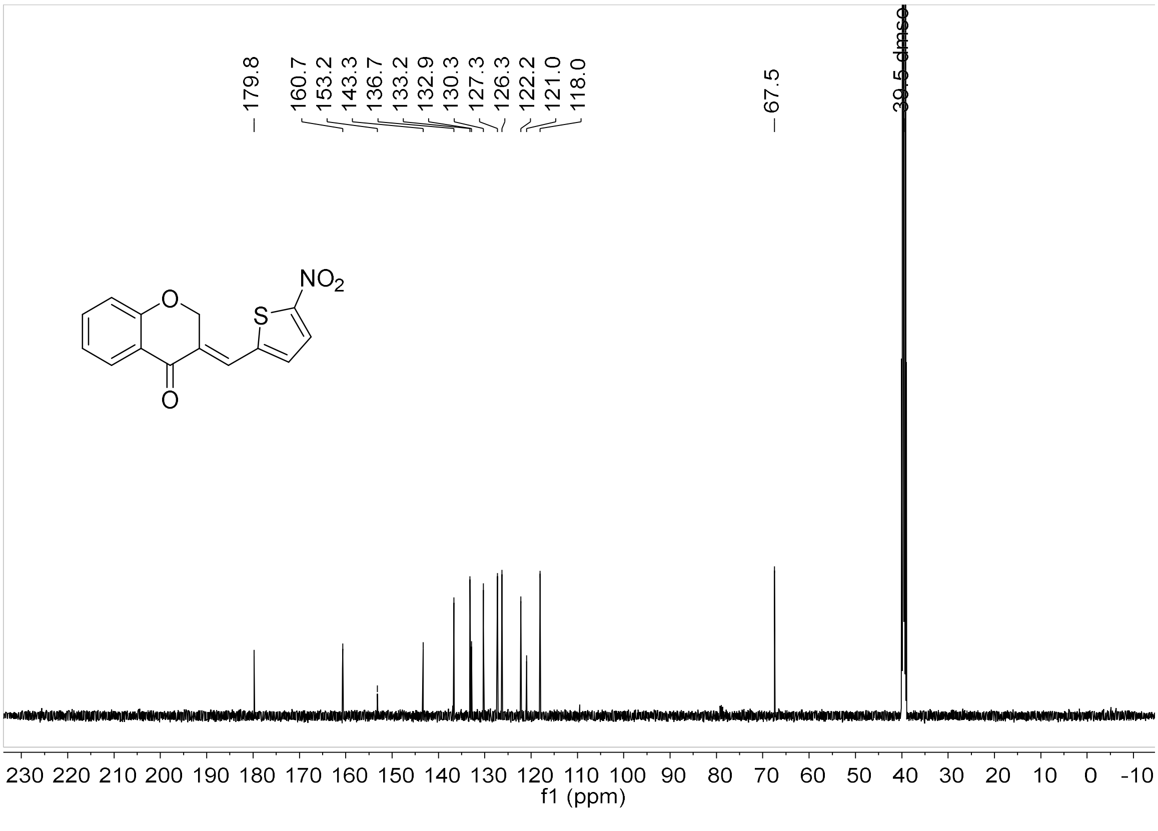

^{1}H NMR (500 MHz, CDCl$_3$) spectrum of 24

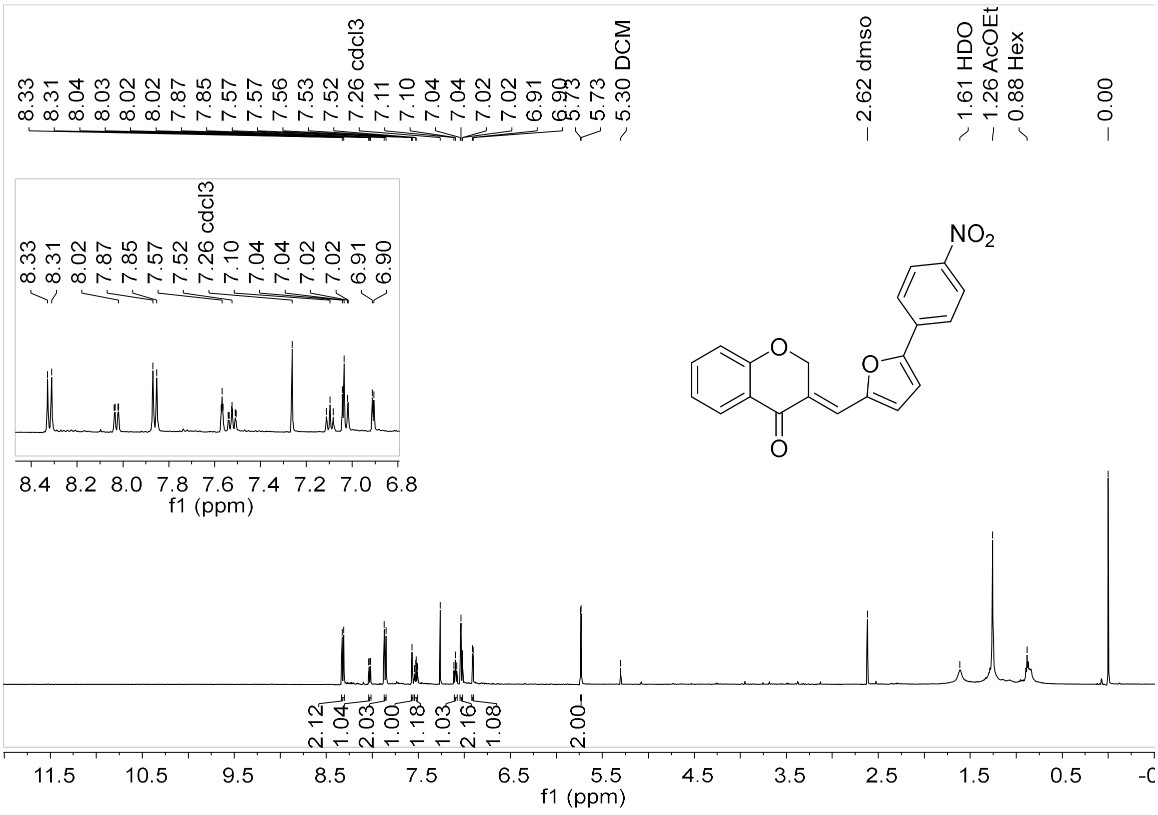

^{13}C NMR (126 MHz, CDCl$_3$) spectrum of 24

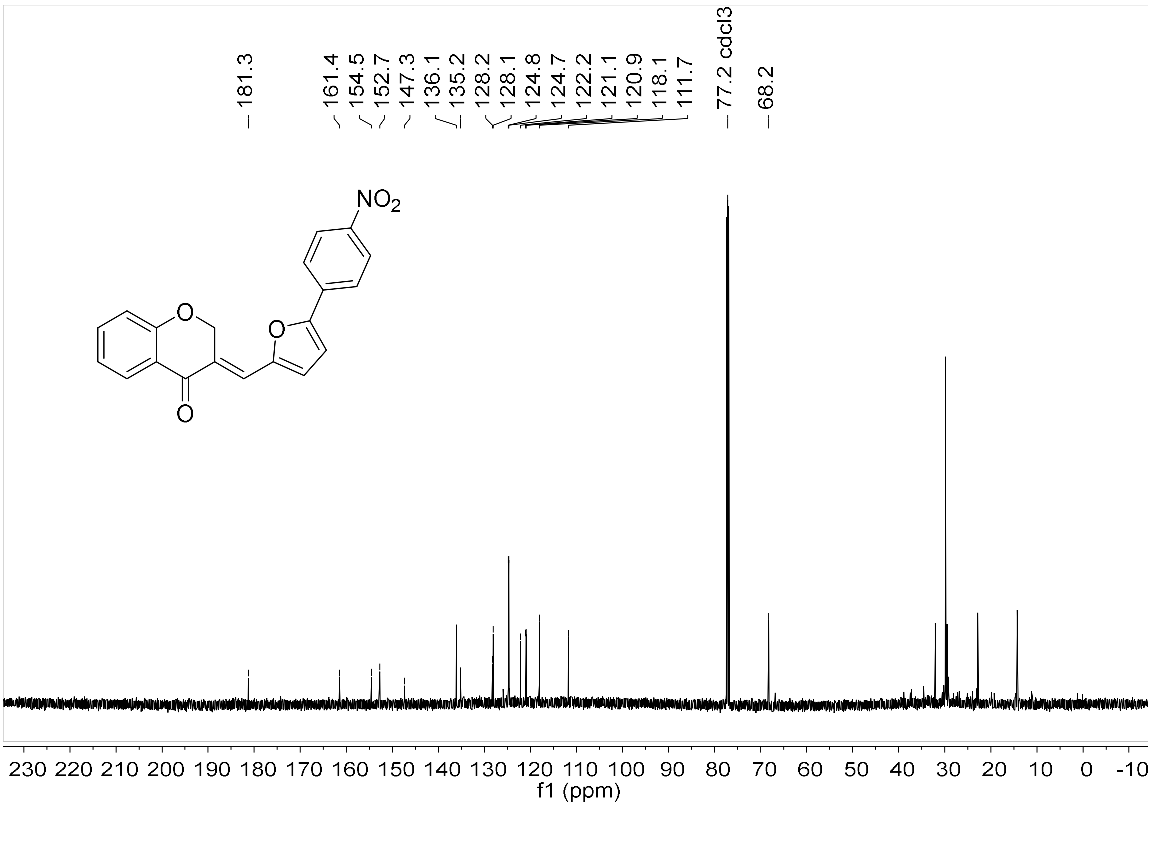

^{1}H NMR (500 MHz, CDCl$_3$) spectrum of 25

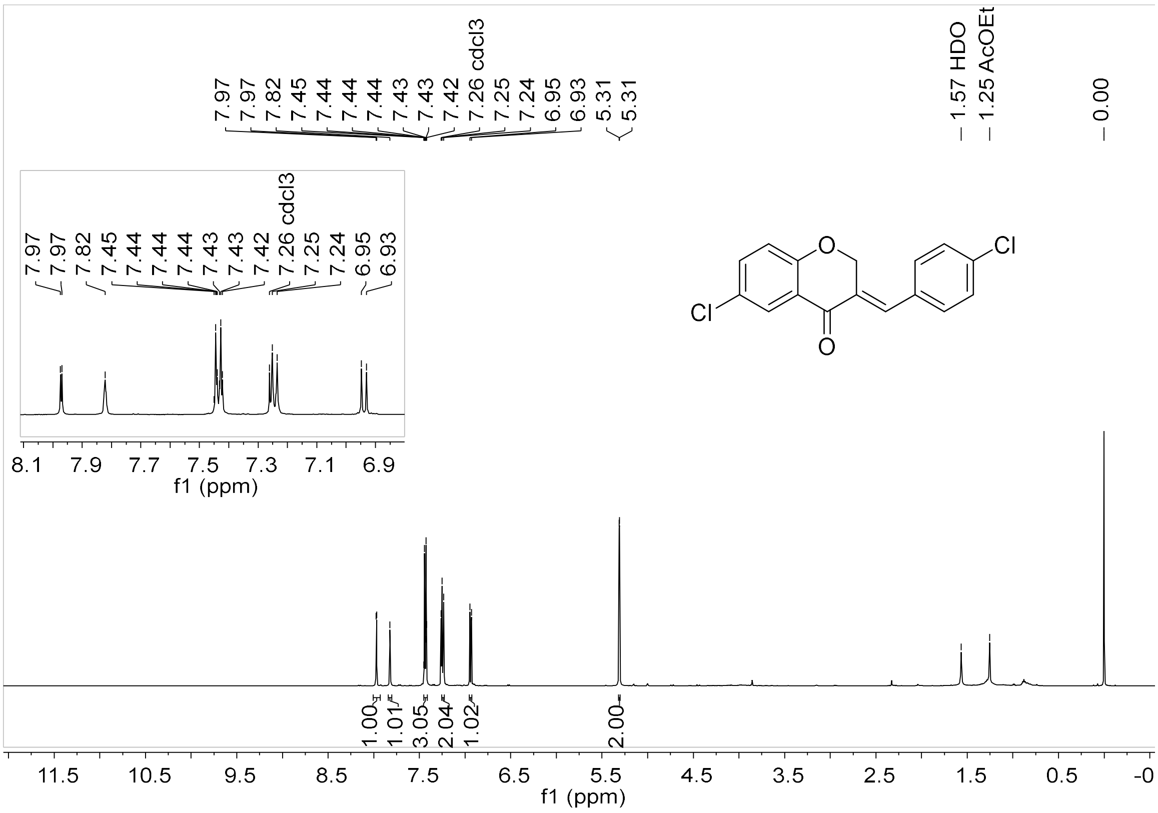

^{13}C NMR (126 MHz, CDCl$_3$) of 25

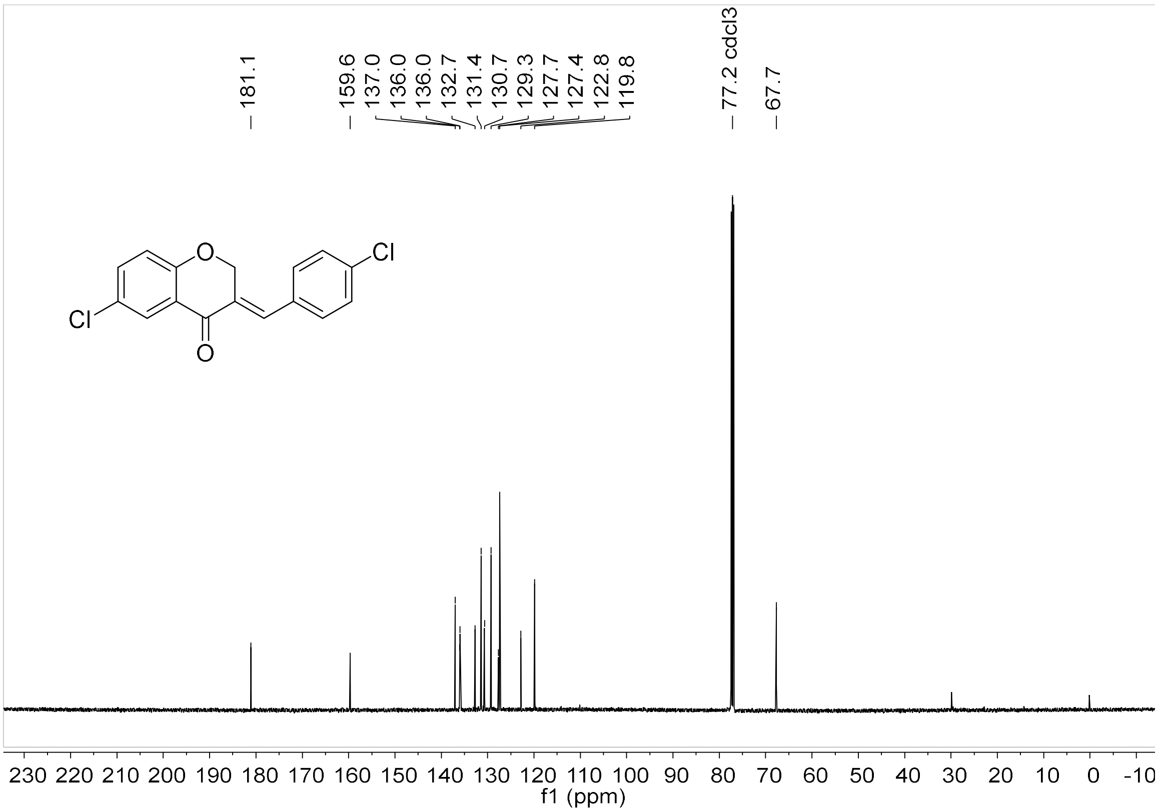

^{1}H NMR (500 MHz, $CDCl_3$) spectrum of 26

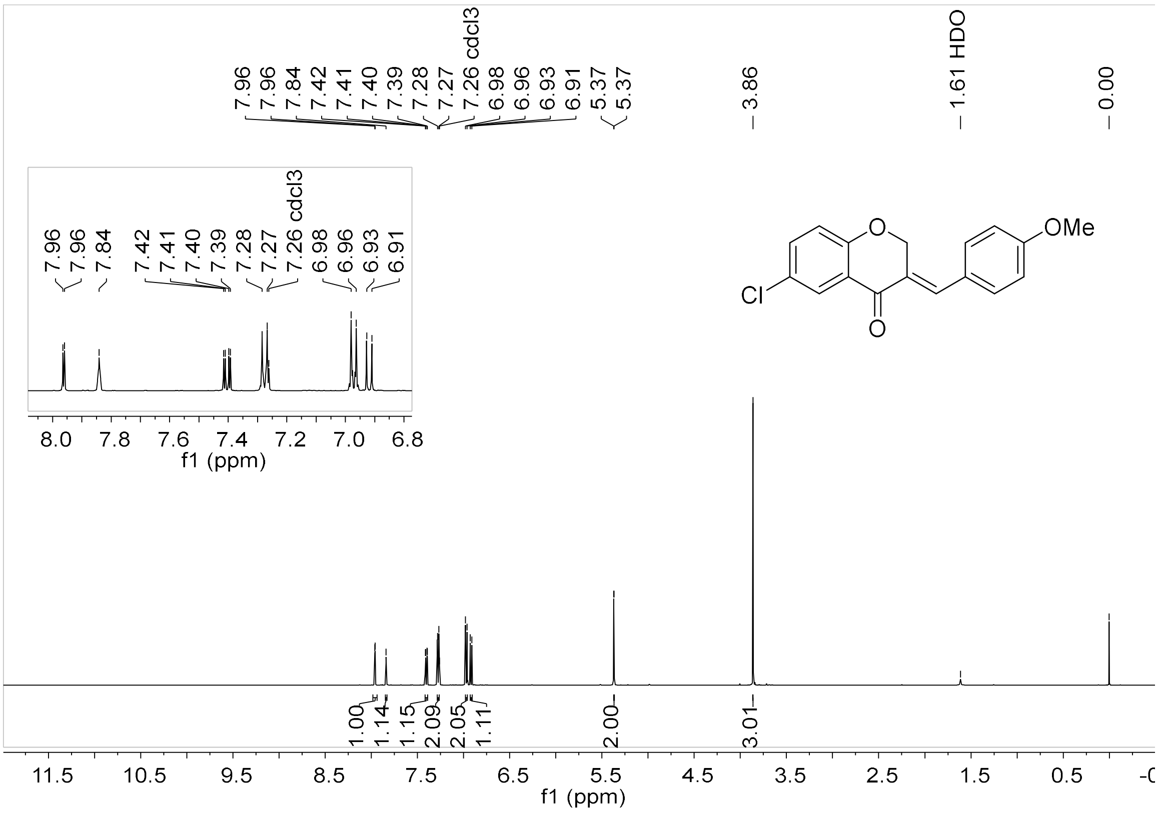

^{13}C NMR (126 MHz, $CDCl_3$) of 26

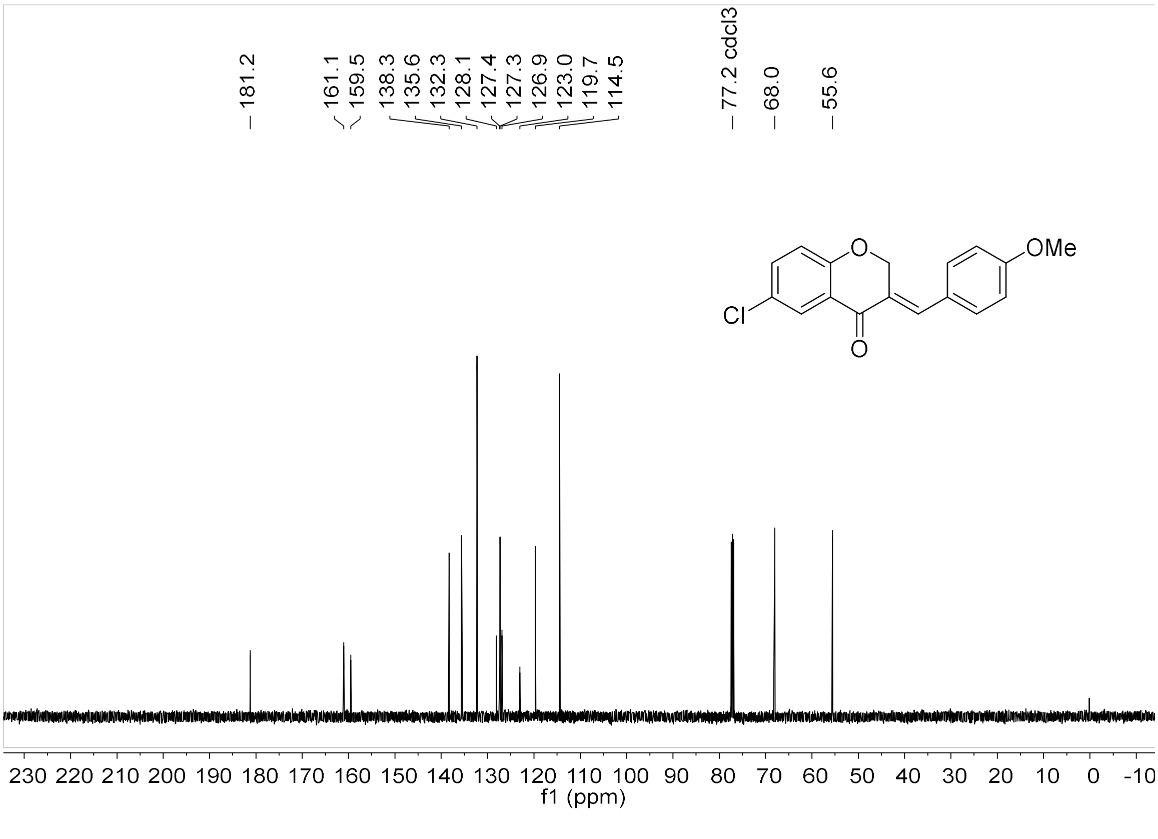

^{1}H NMR (500 MHz, $CDCl_3$) spectrum of 27

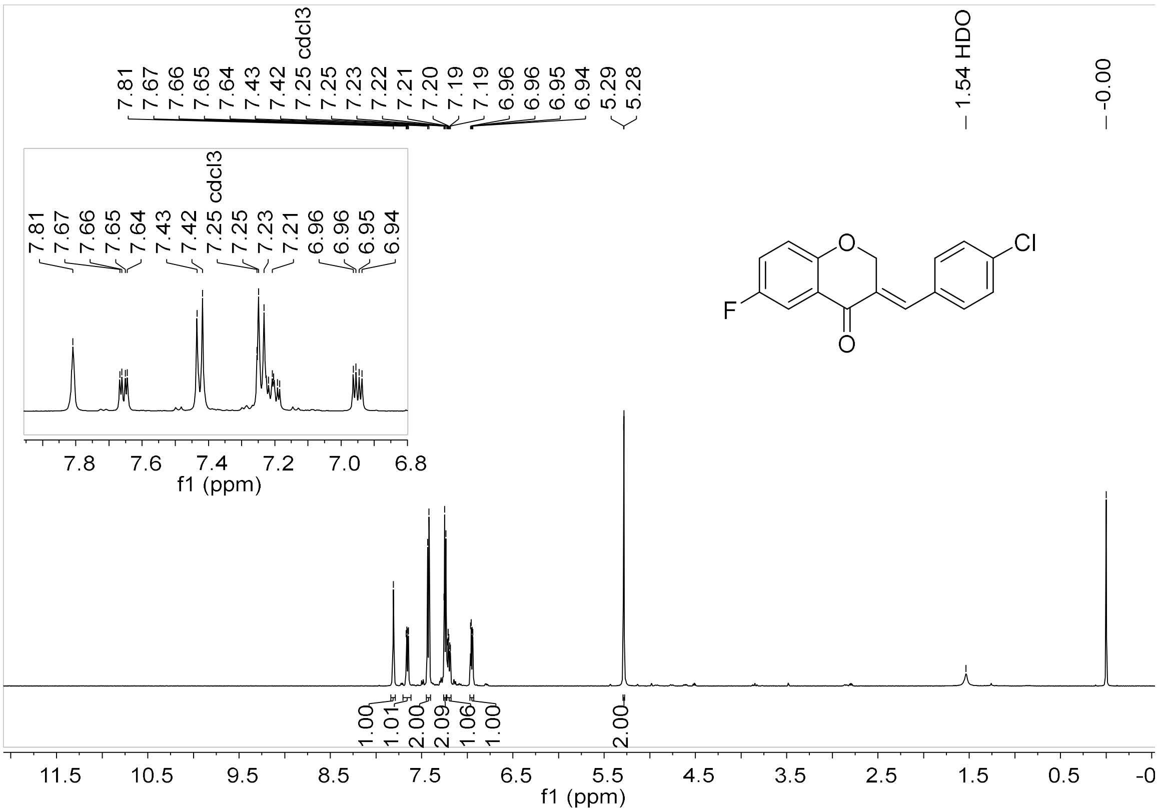

^{13}C NMR (126 MHz, $CDCl_3$) of 27

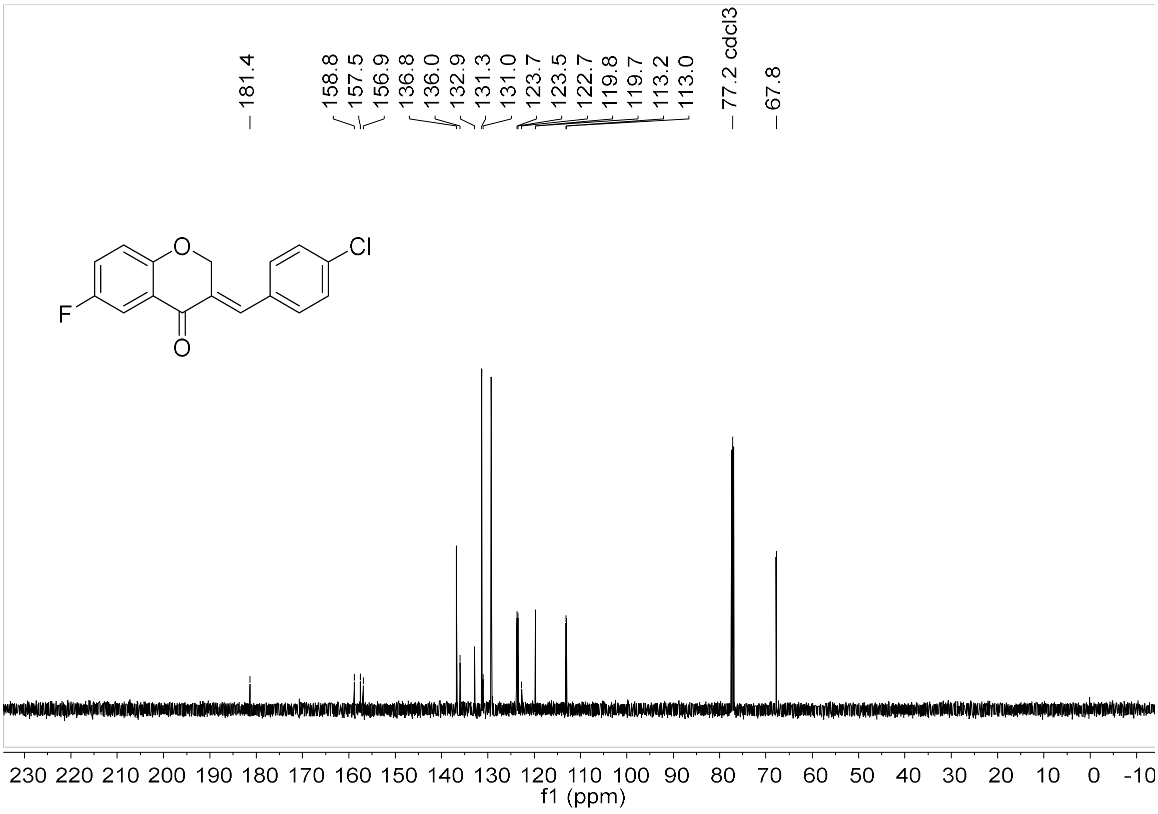

^{1}H NMR (500 MHz, CDCl$_3$) spectrum of 28

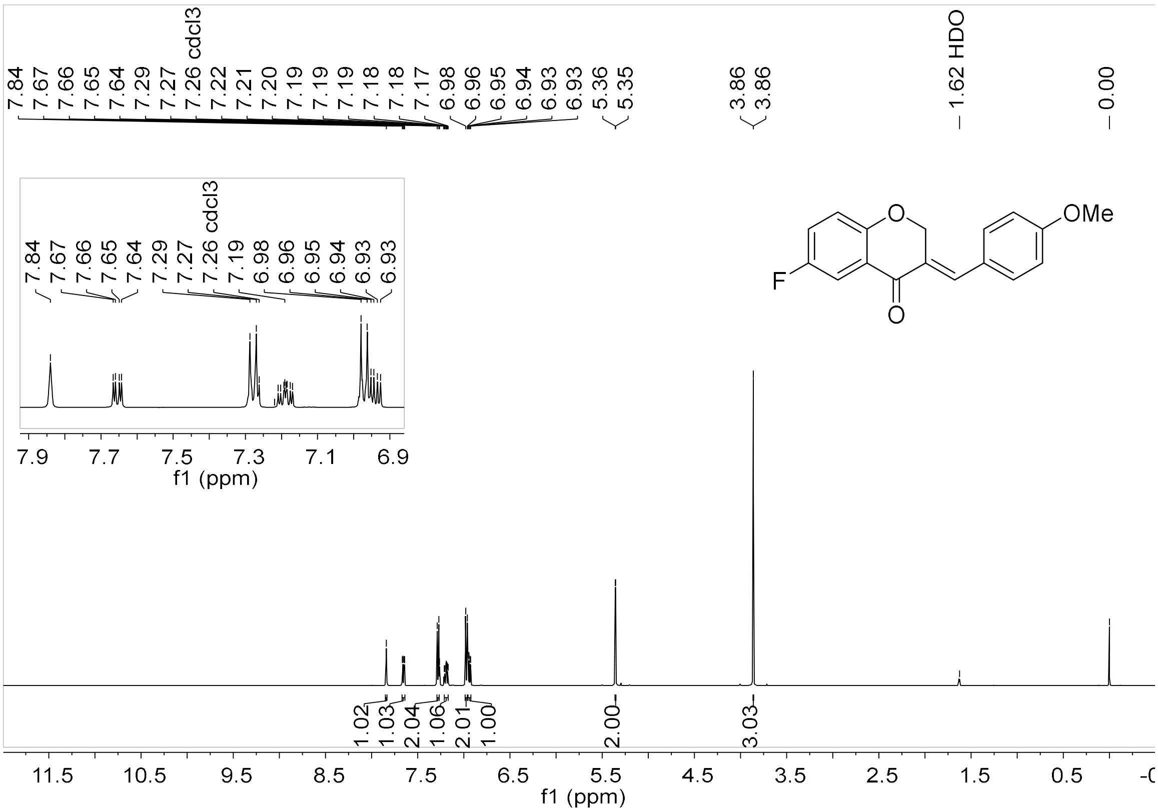

^{13}C NMR (126 MHz, CDCl$_3$) of 28

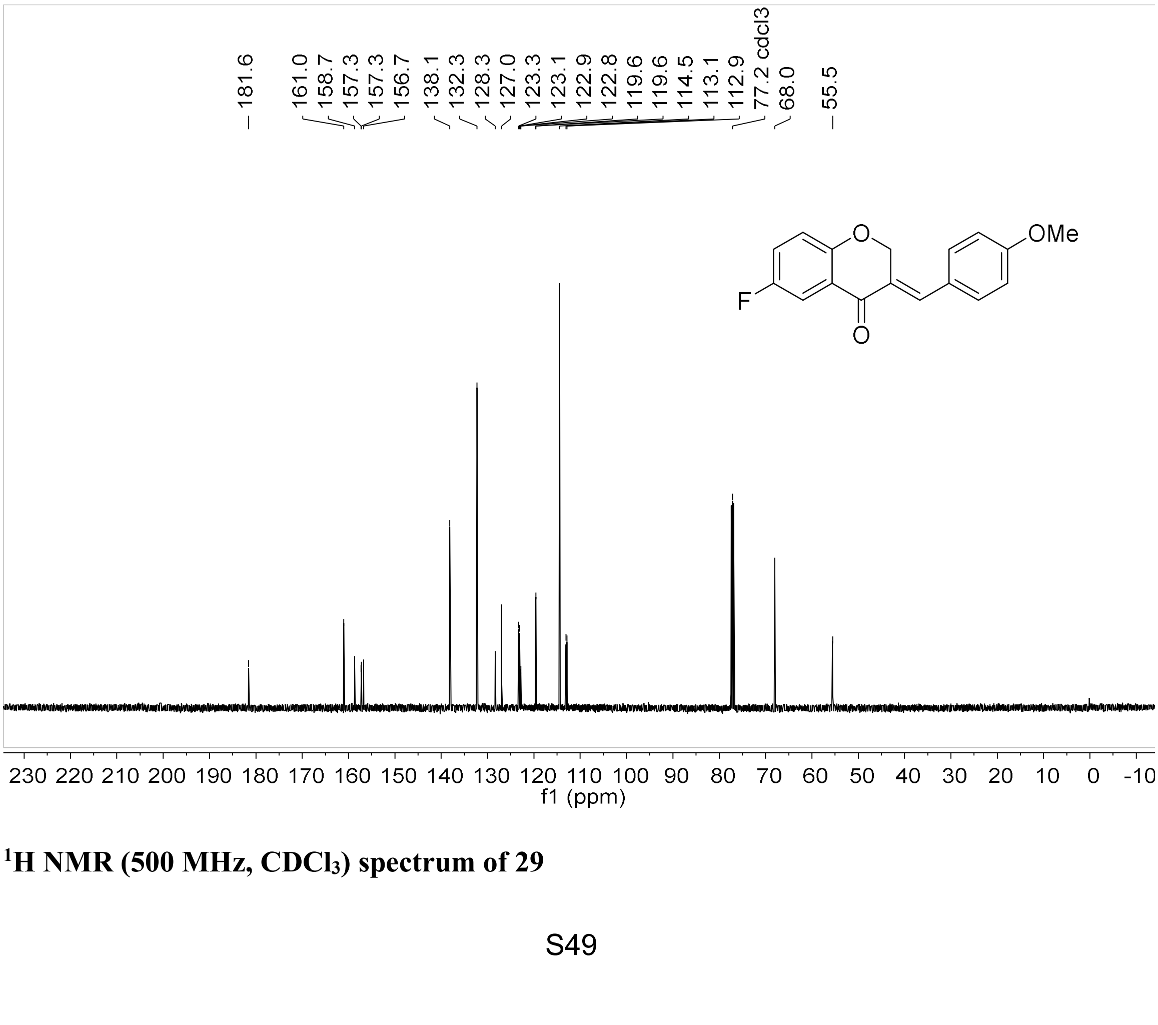

^{1}H NMR (500 MHz, CDCl$_3$) spectrum of 29

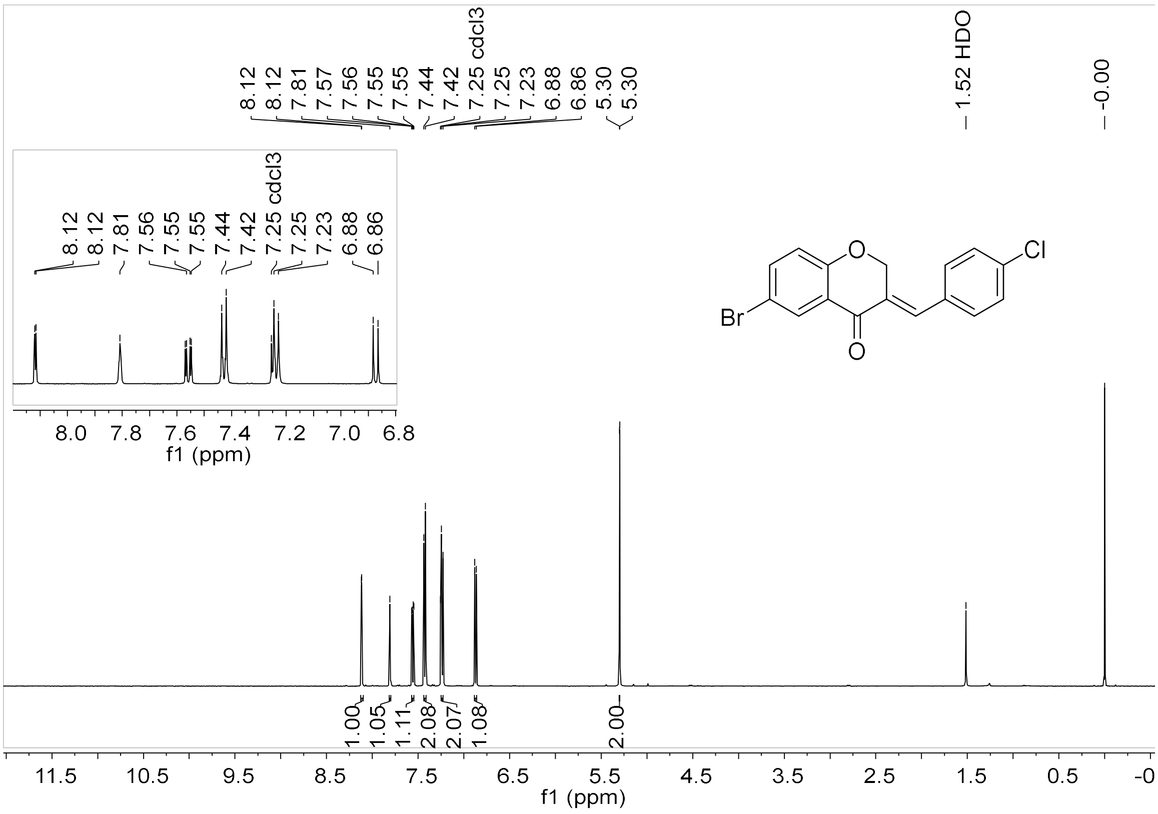

^{13}C NMR (126 MHz, CDCl₃) of 29

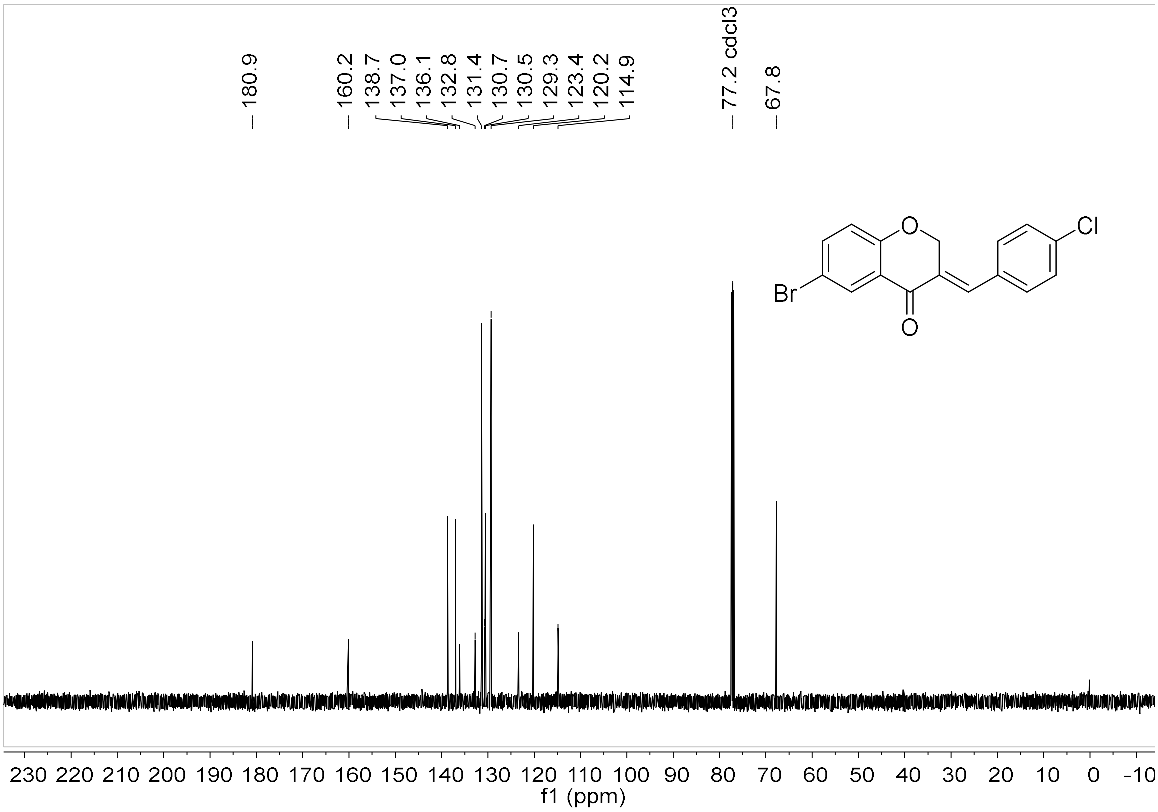

^{1}H NMR (500 MHz, $CDCl_3$) spectrum of 30

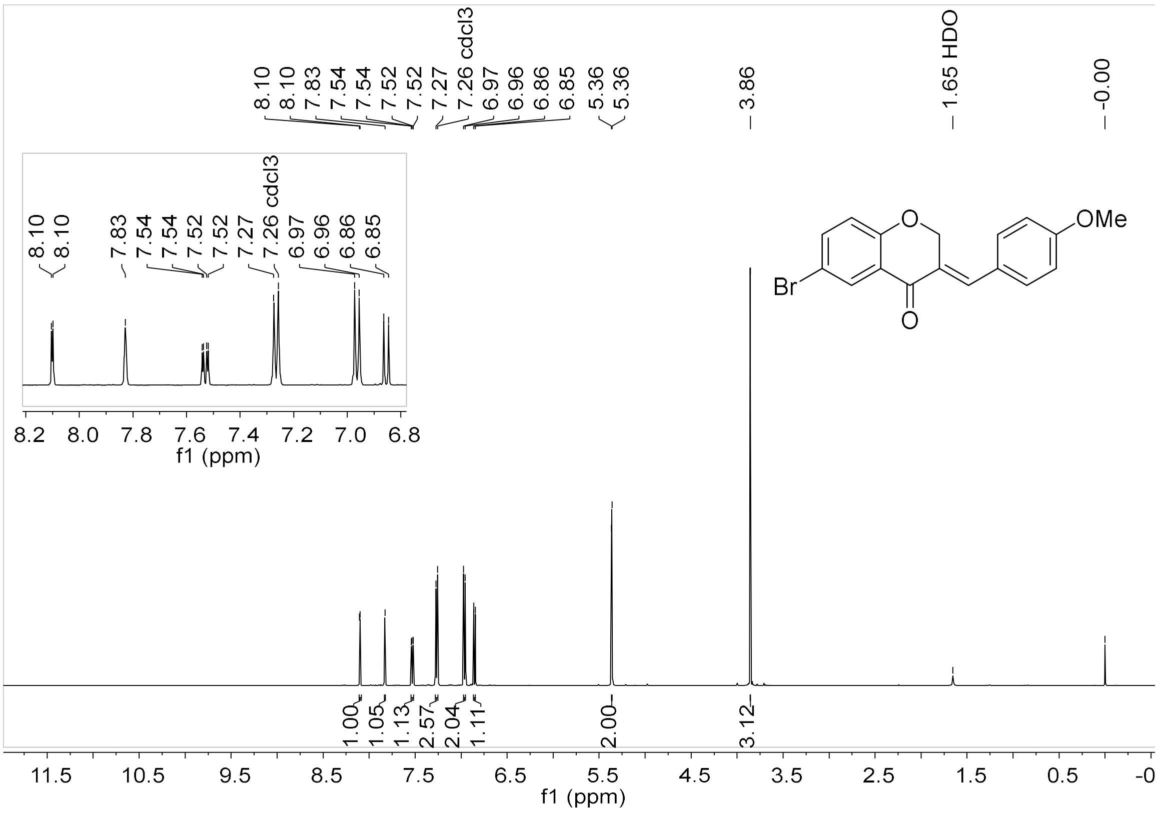

^{13}C NMR (126 MHz, $CDCl_3$) of 30

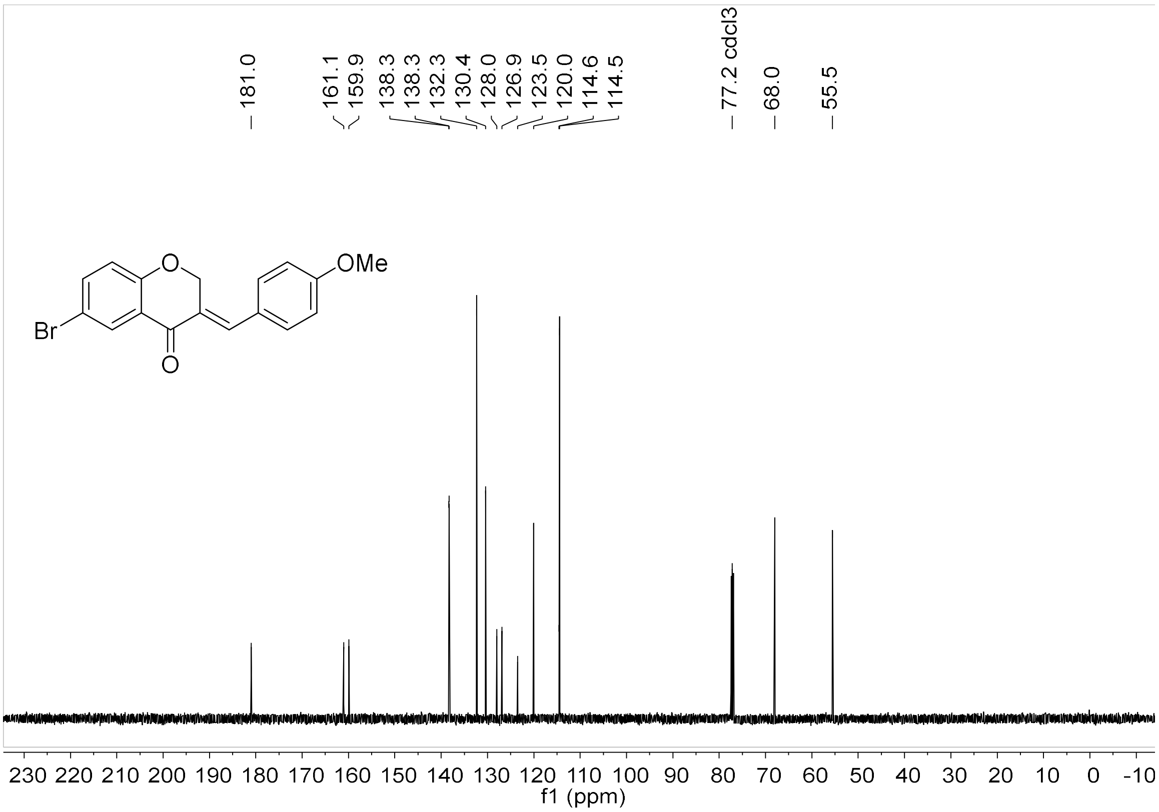

^{1}H NMR (400 MHz, CDCl$_3$) spectrum of 31

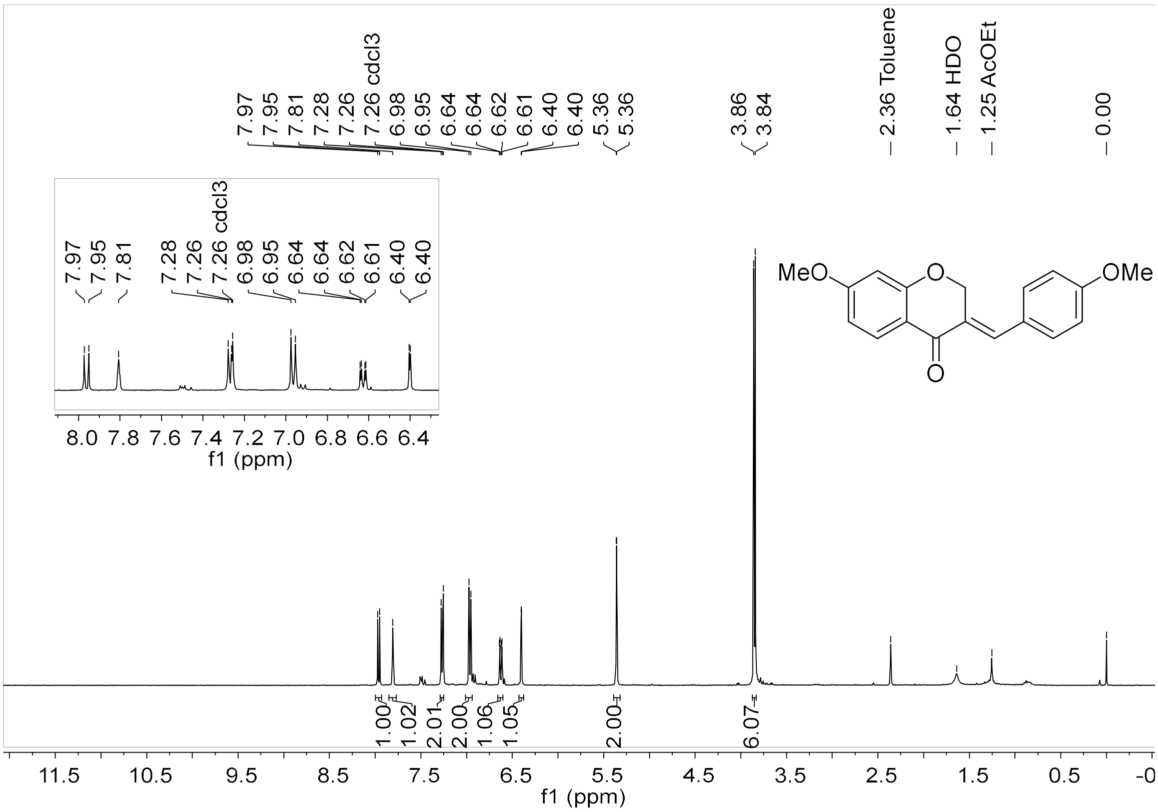

^{13}C NMR (101 MHz, CDCl$_3$) of 31

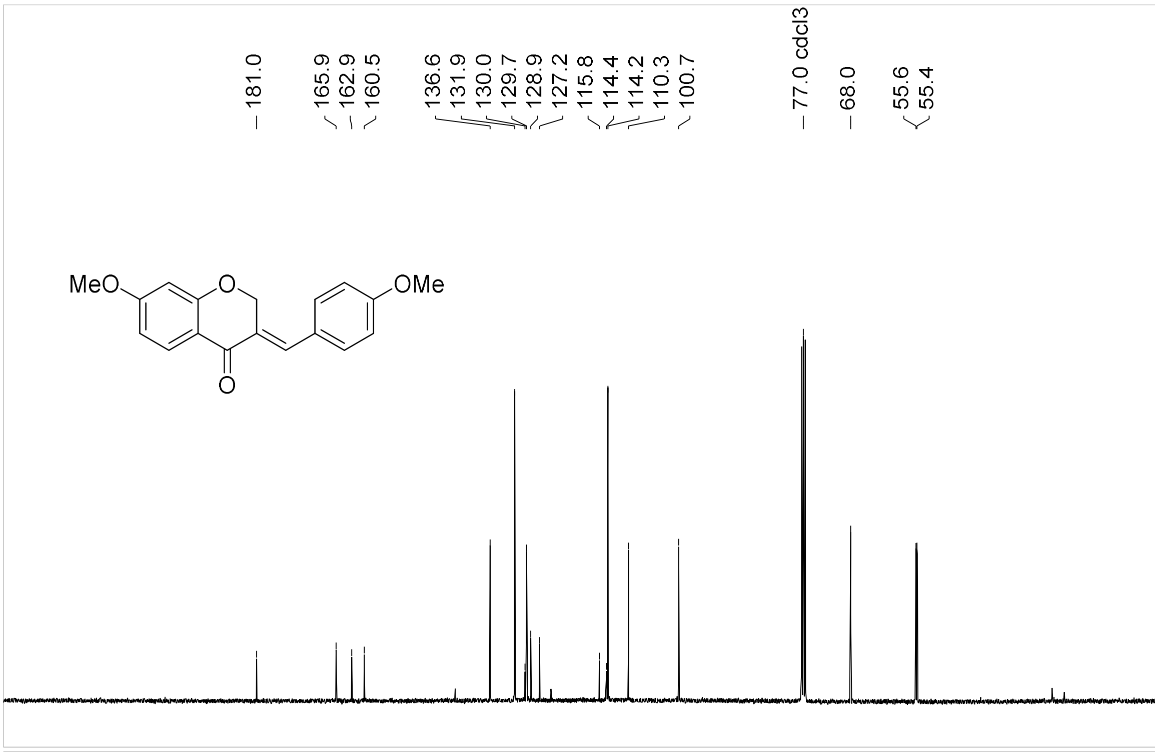

^{1}H NMR (400 MHz, $CDCl_3$) spectrum of 32

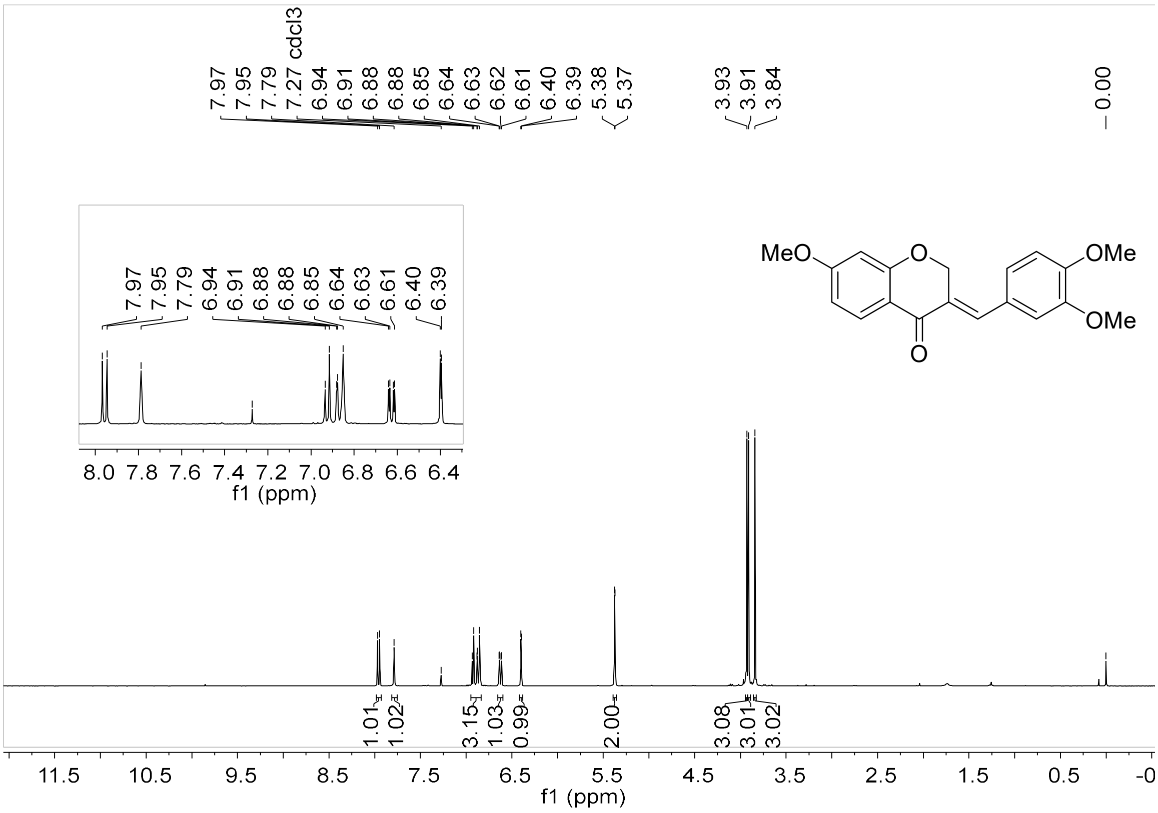

^{13}C NMR (101 MHz, $CDCl_3$) of 32

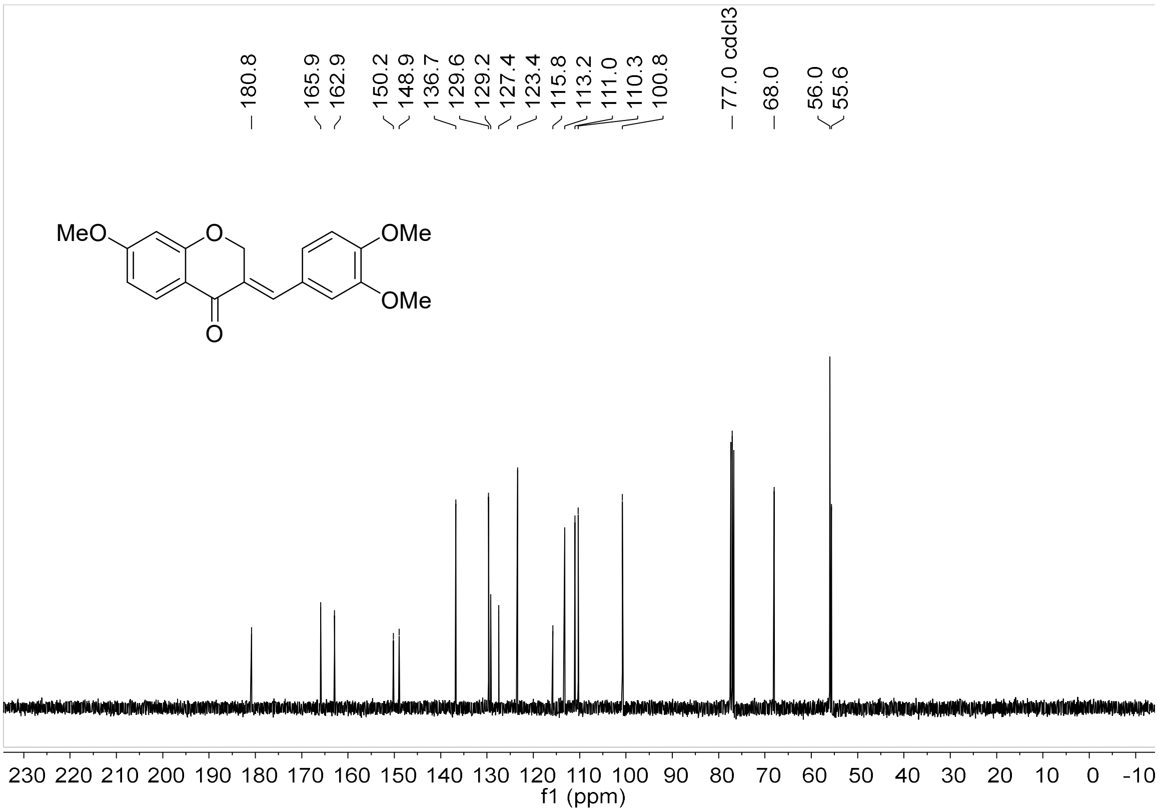

^{1}H NMR (500 MHz, DMSO-d_6) spectrum of 33

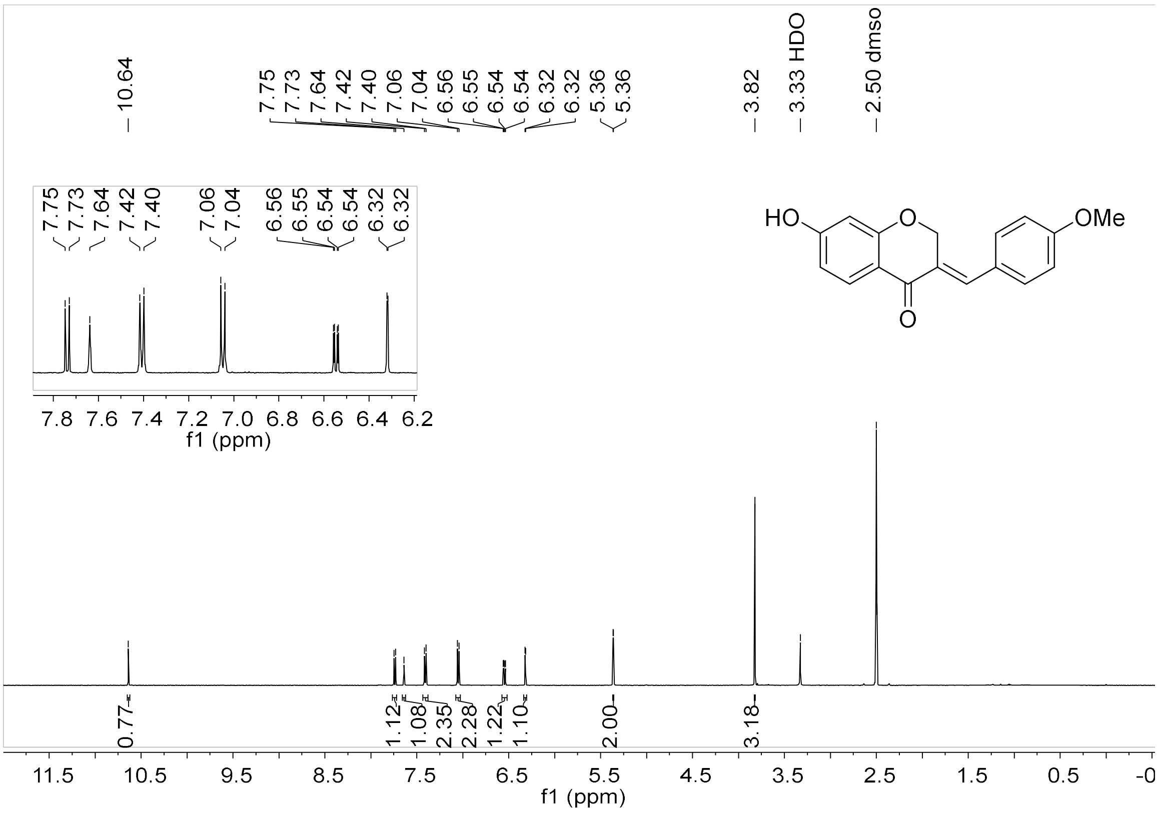

^{13}C NMR (126 MHz, DMSO-d_6) of 33

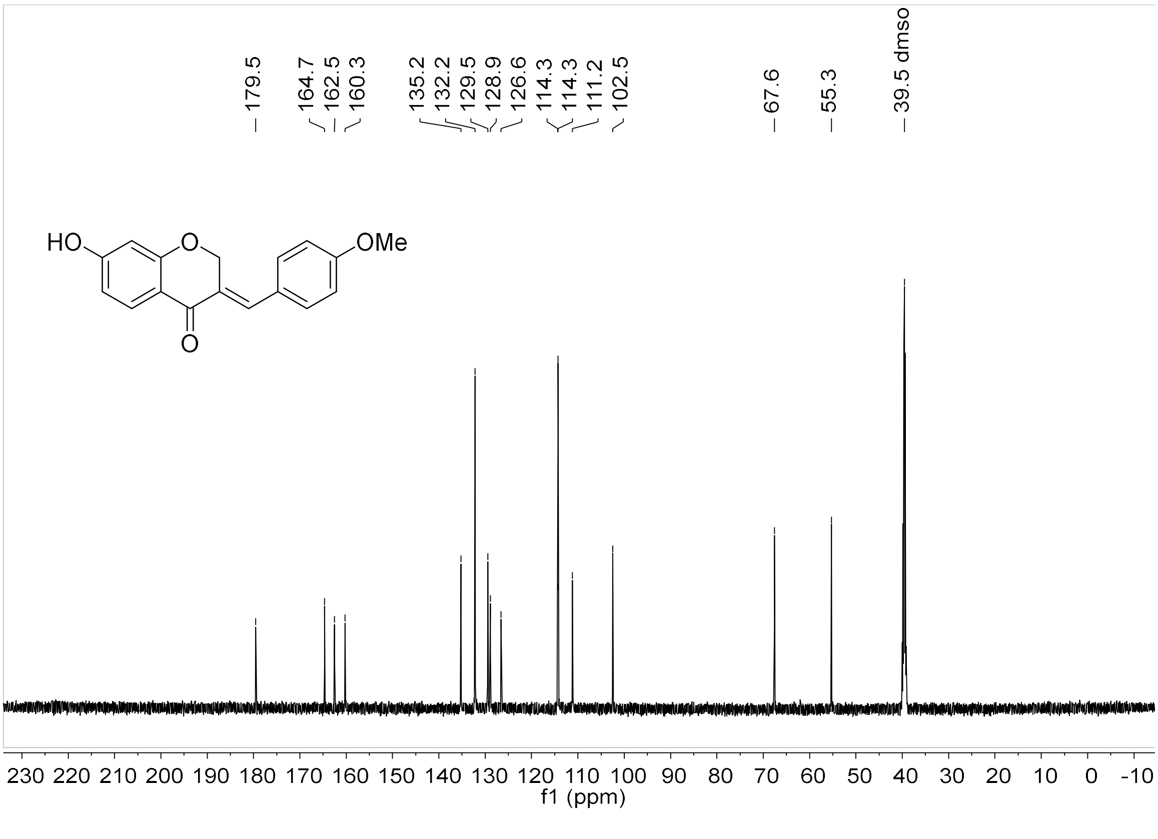

^{1}H NMR (500 MHz, DMSO-d_6) spectrum of 34

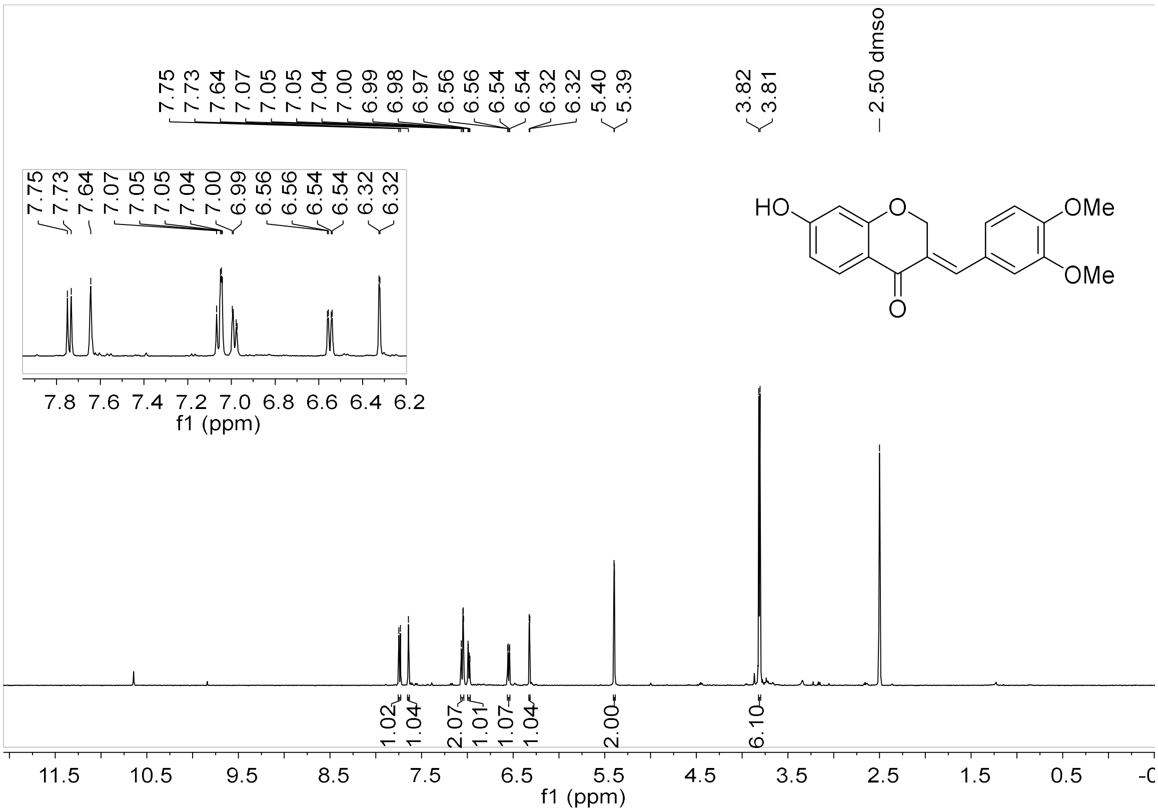

^{13}C NMR (101 MHz, DMSO-d_6) of 34

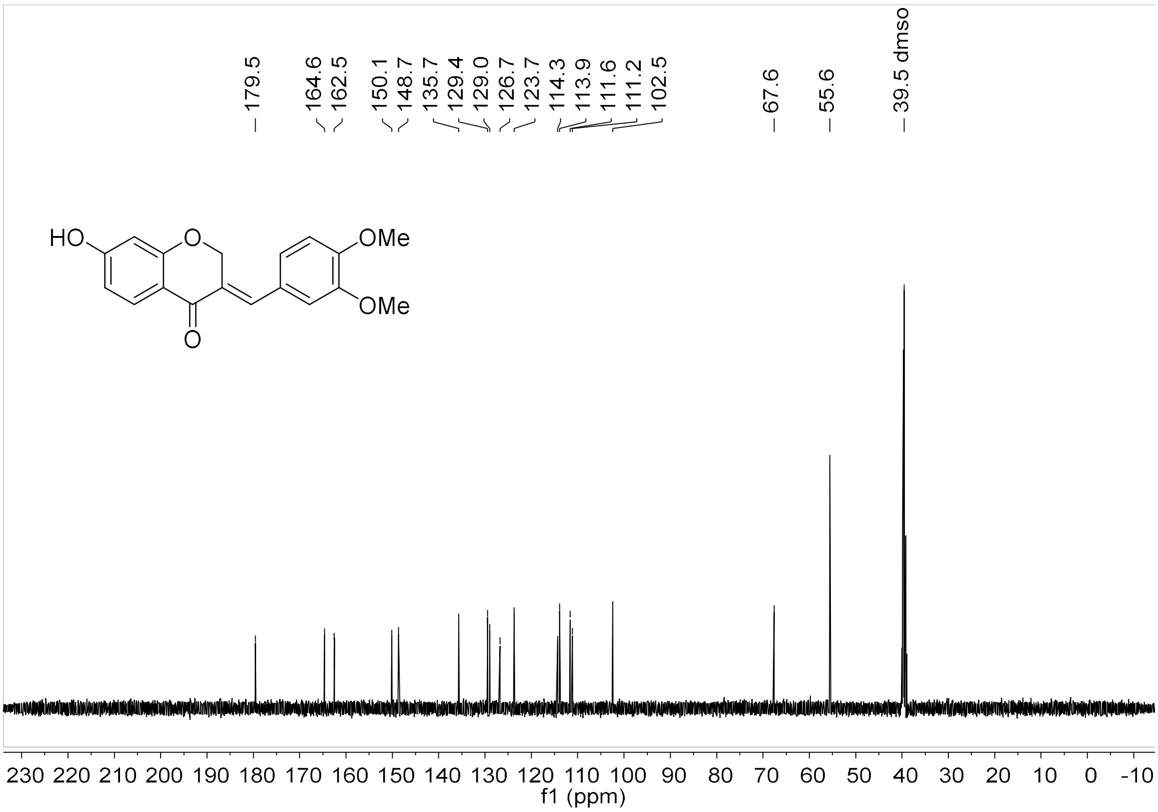

^{1}H NMR (400 MHz, CDCl₃) spectrum of 35

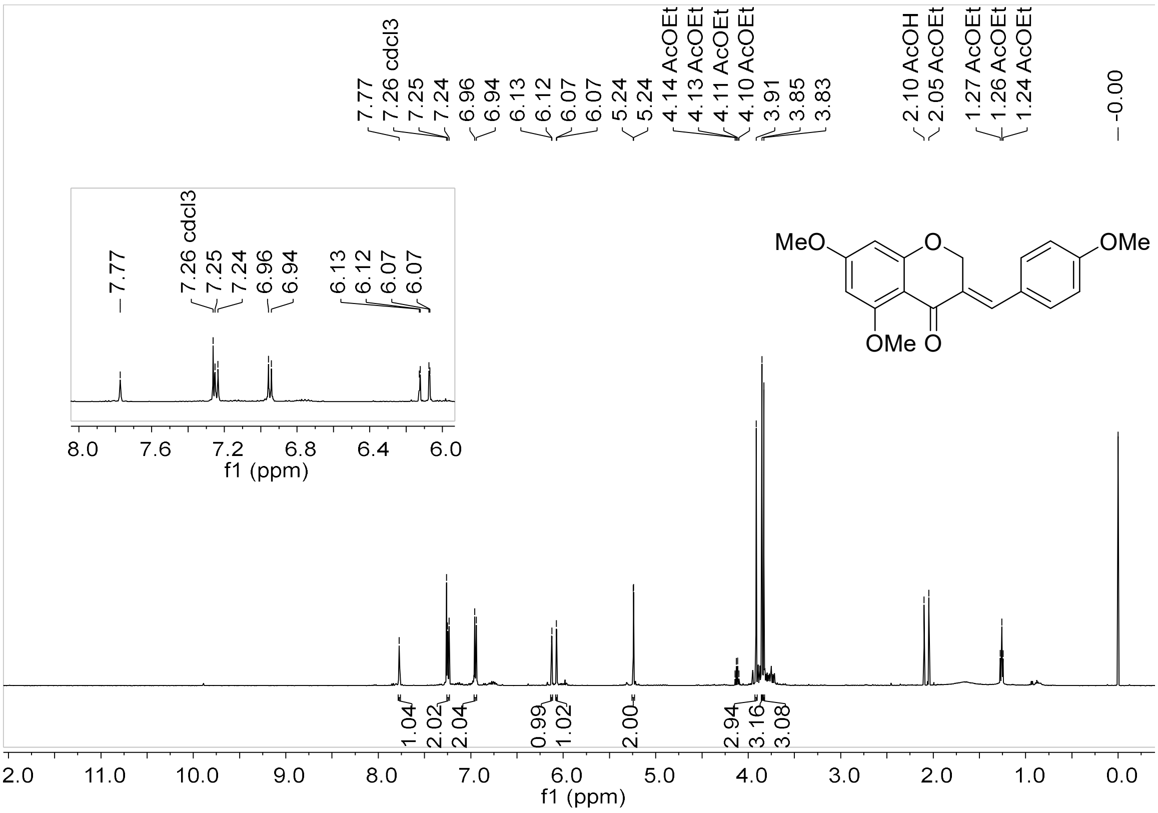

^{13}C NMR (101 MHz, CDCl₃) of 35

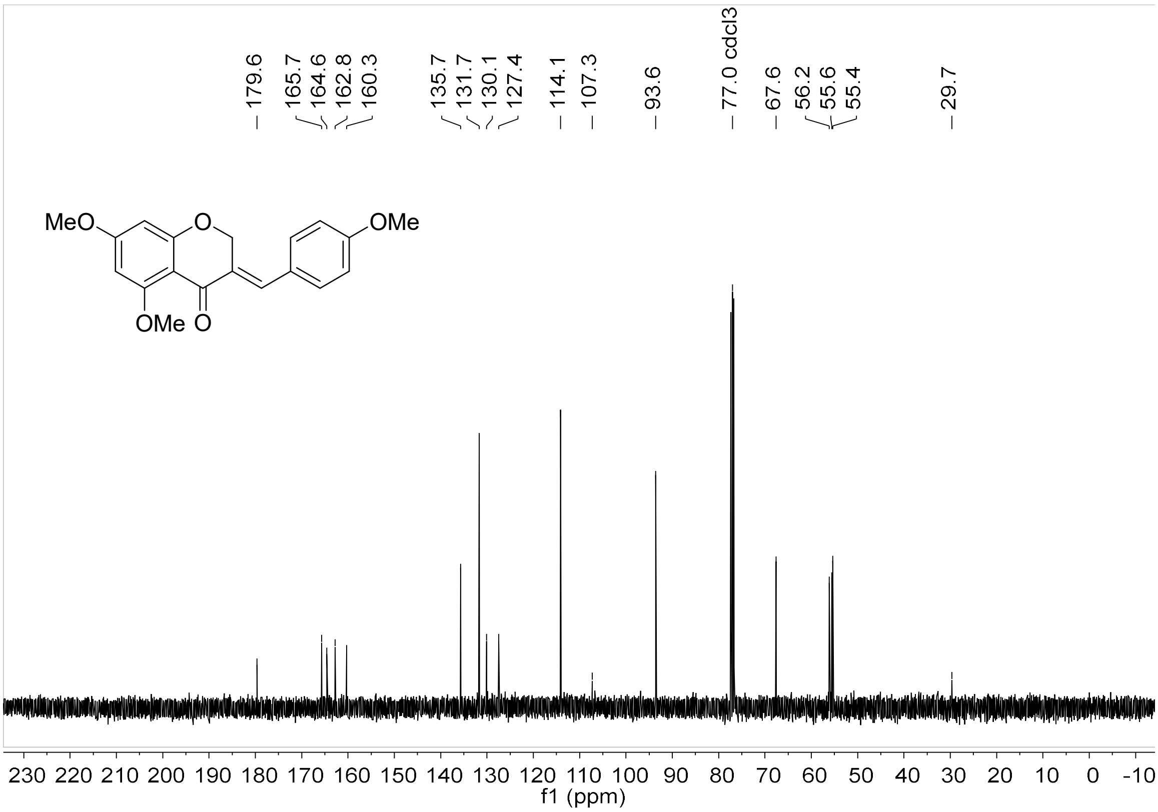

^{1}H NMR (500 MHz, DMSO-d_6) spectrum of 36

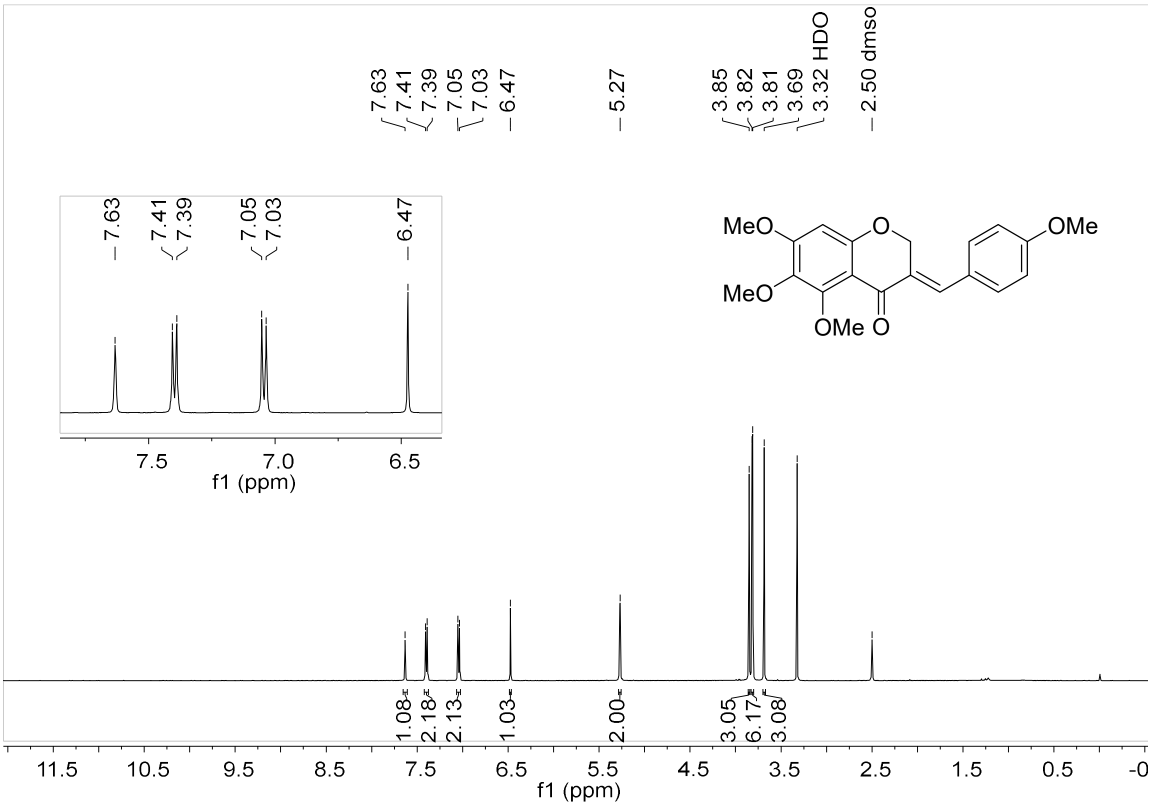

^{13}C NMR (126 MHz, DMSO-d_6) spectrum of 36

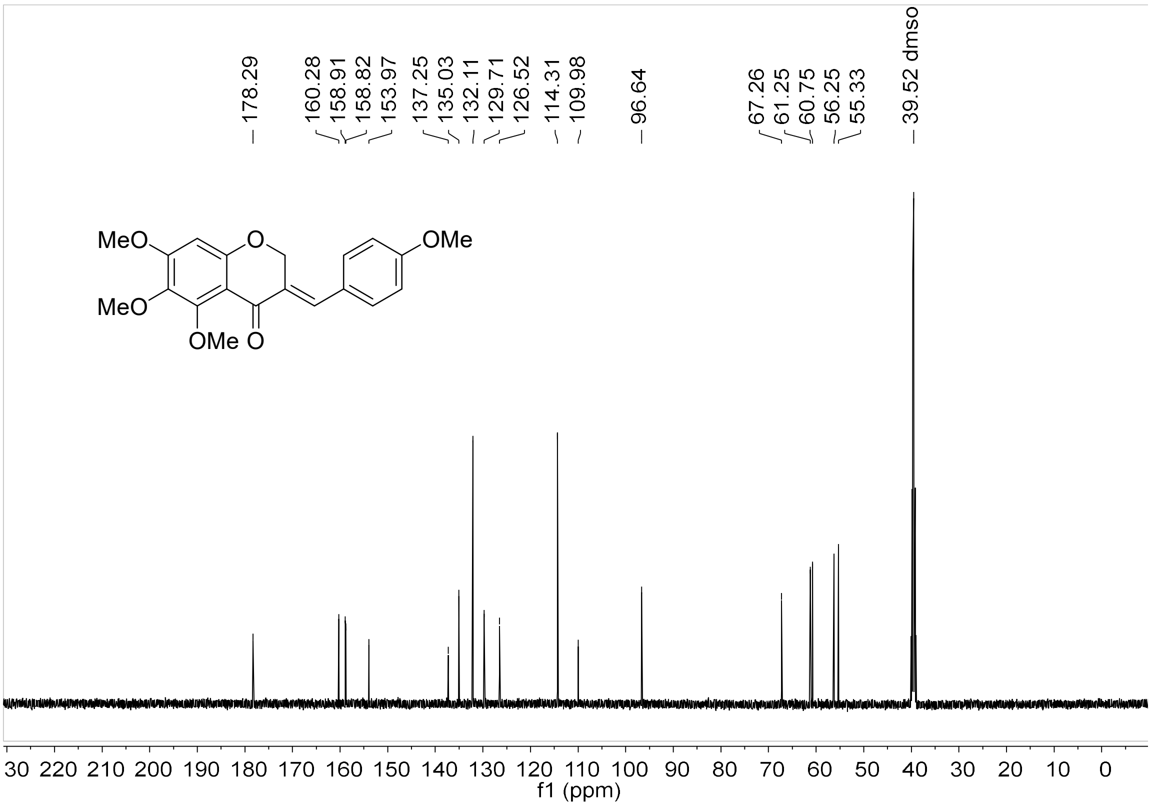

^{1}H NMR (400 MHz, CDCl₃) spectrum of 37

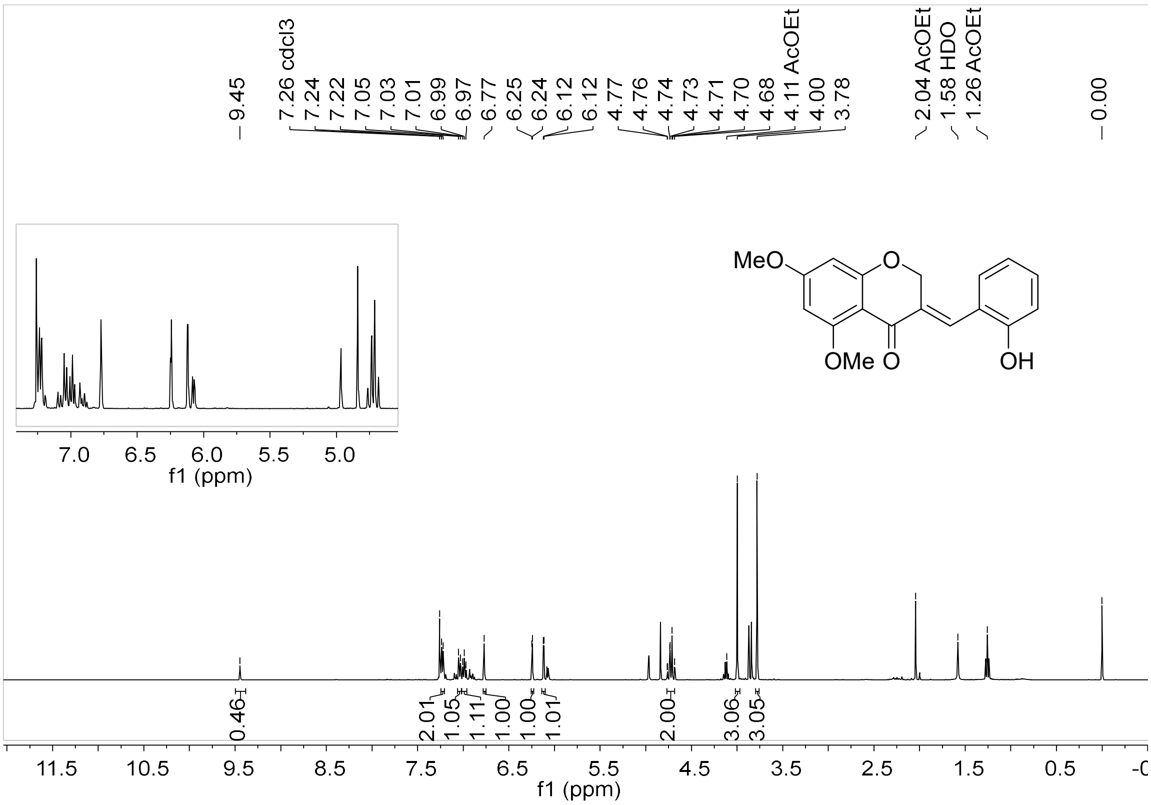

^{13}C NMR (101 MHz, Acetone-d_6) spectrum of 37

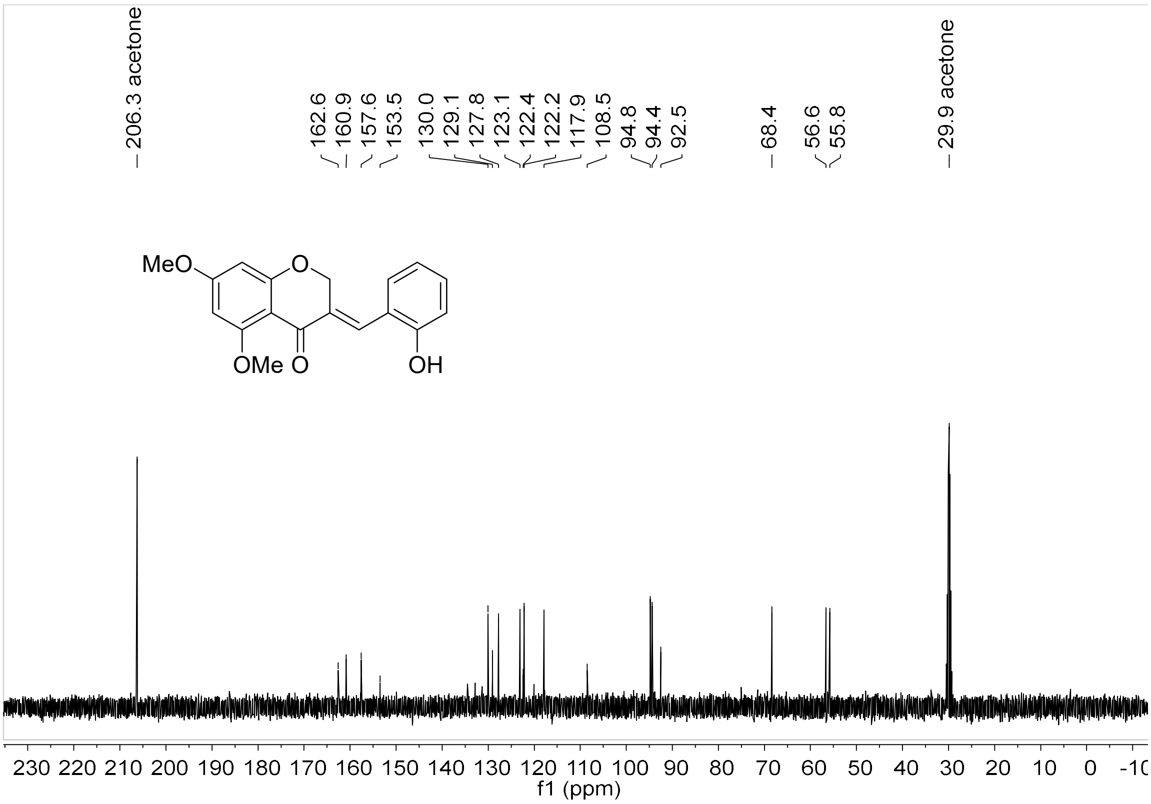

^{1}H NMR (500 MHz, CDCl$_3$) spectrum of 38

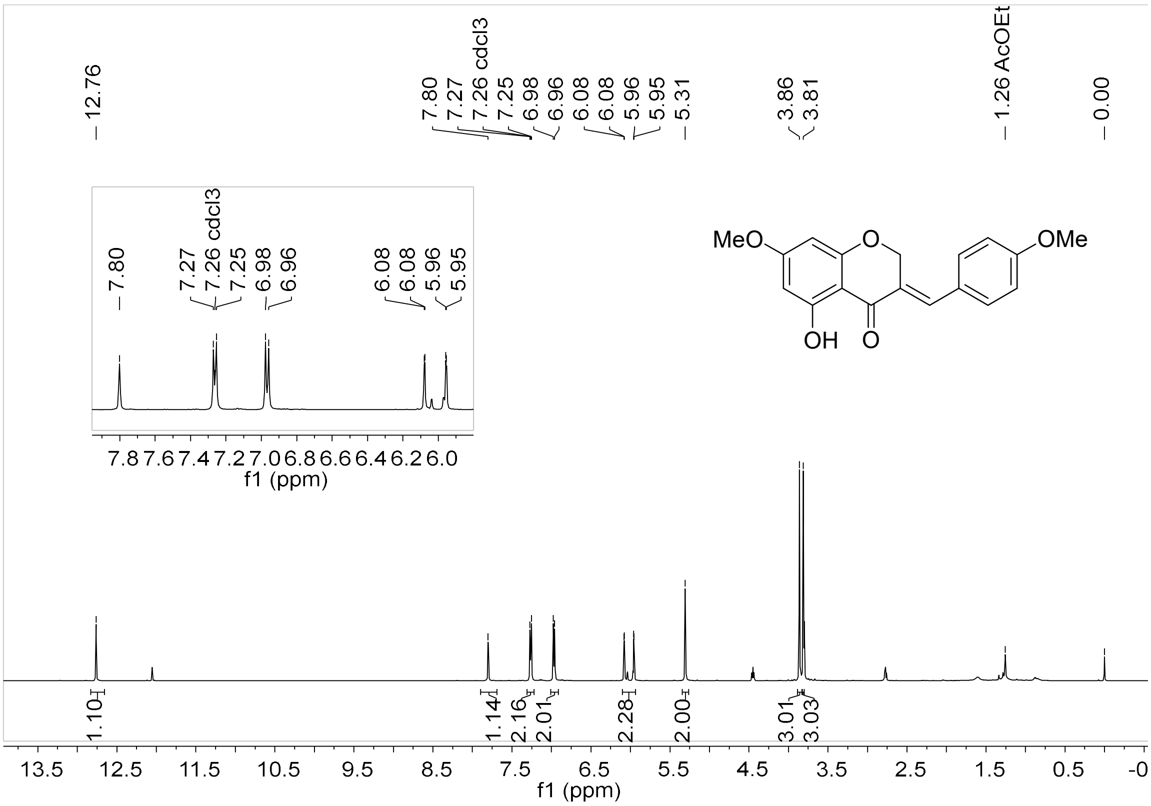

^{13}C NMR (126 MHz, CDCl$_3$) spectrum of 38

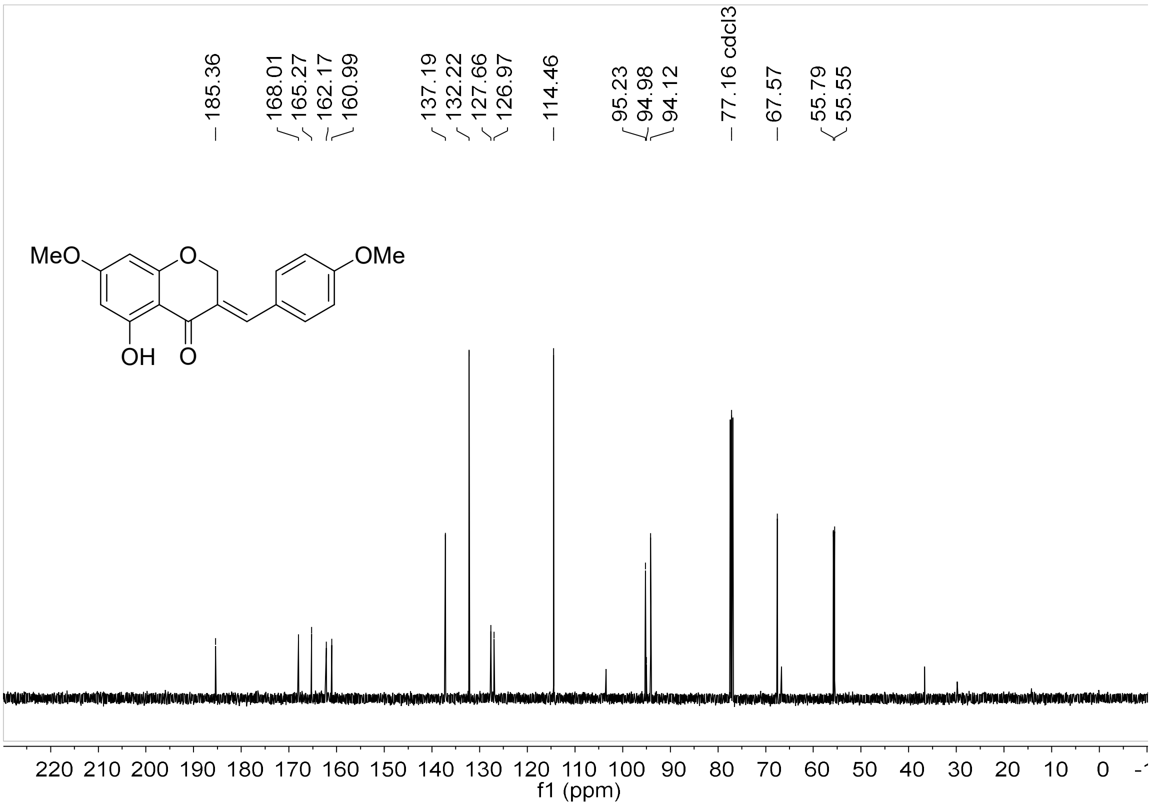

^{1}H NMR (500 MHz, $CDCl_3$) spectrum of 39

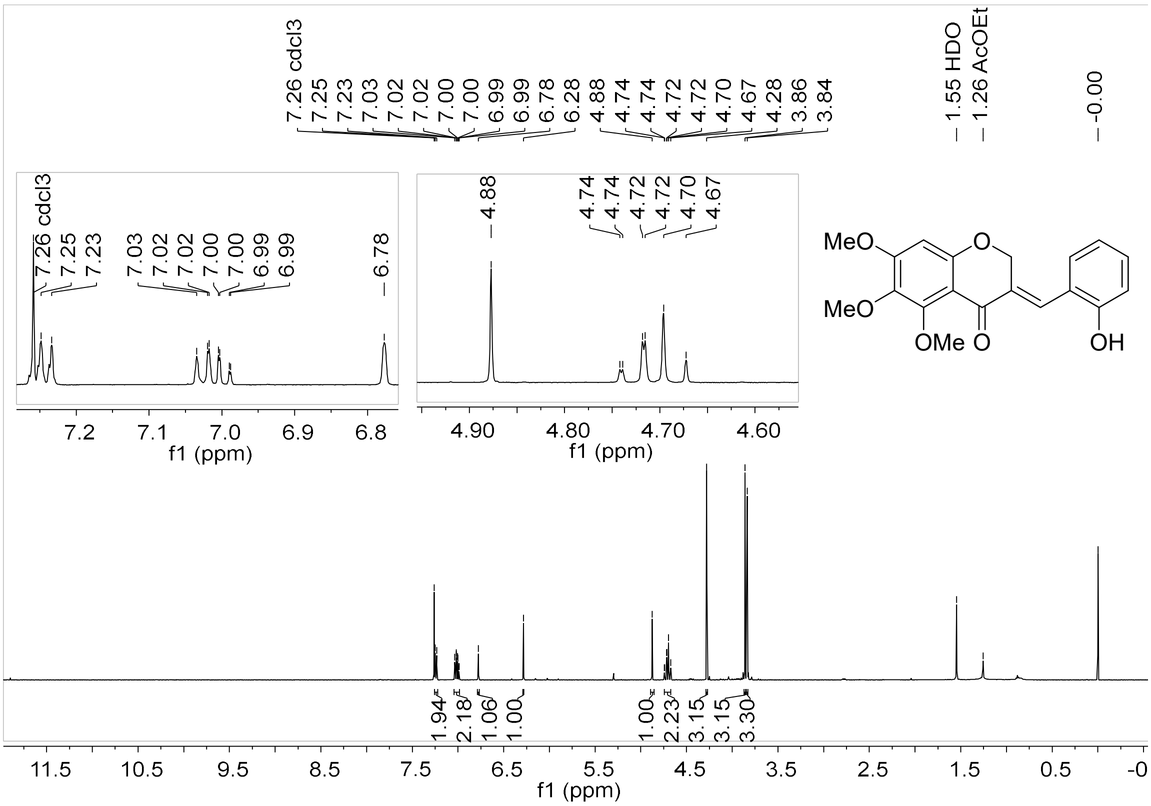

^{13}C NMR (126 MHz, Acetone-d_6) spectrum of 39

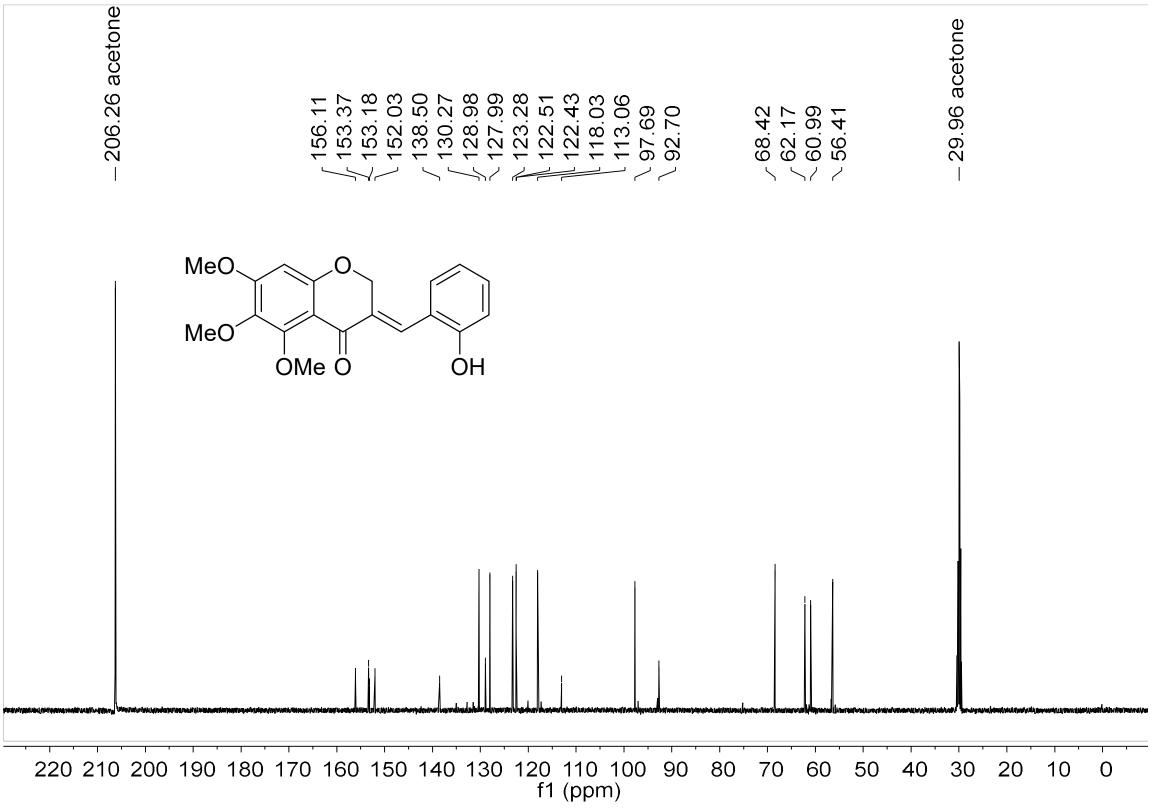

^{1}H NMR (400 MHz, $CDCl_3$) spectrum of 40

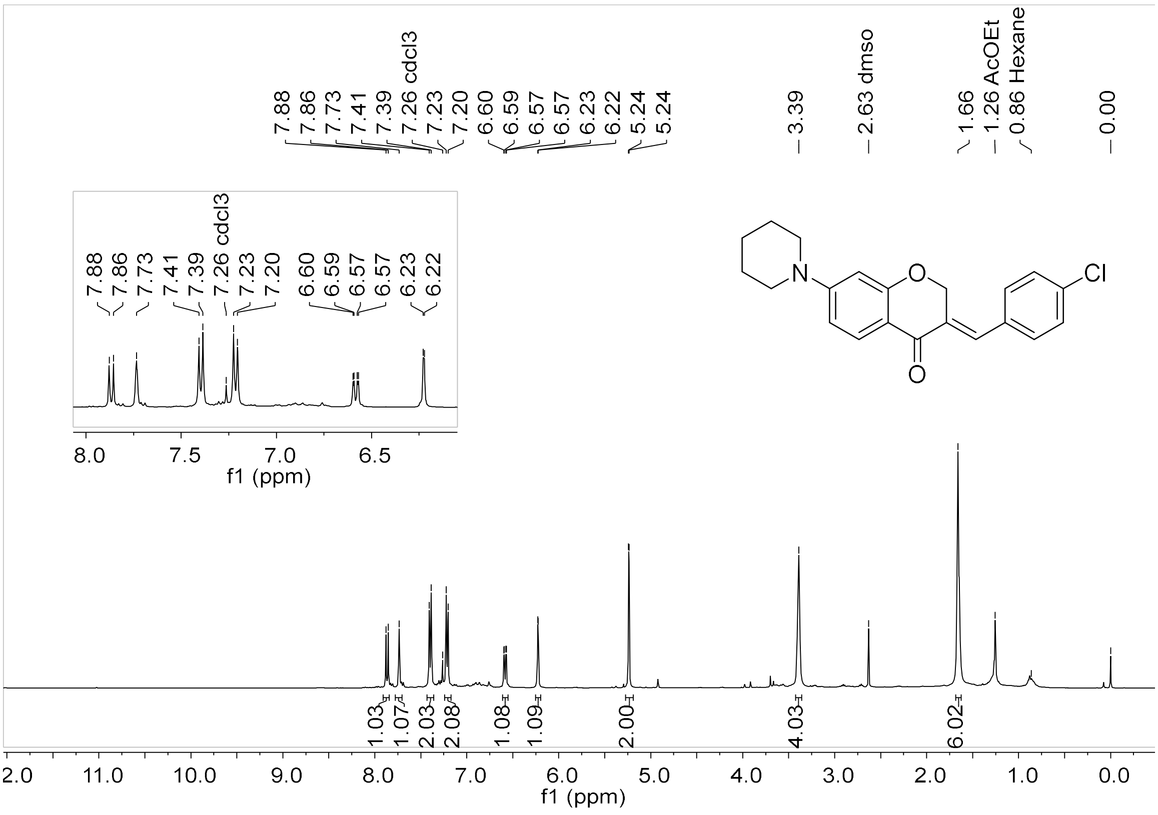

^{13}C NMR (101 MHz, $CDCl_3$) of 40

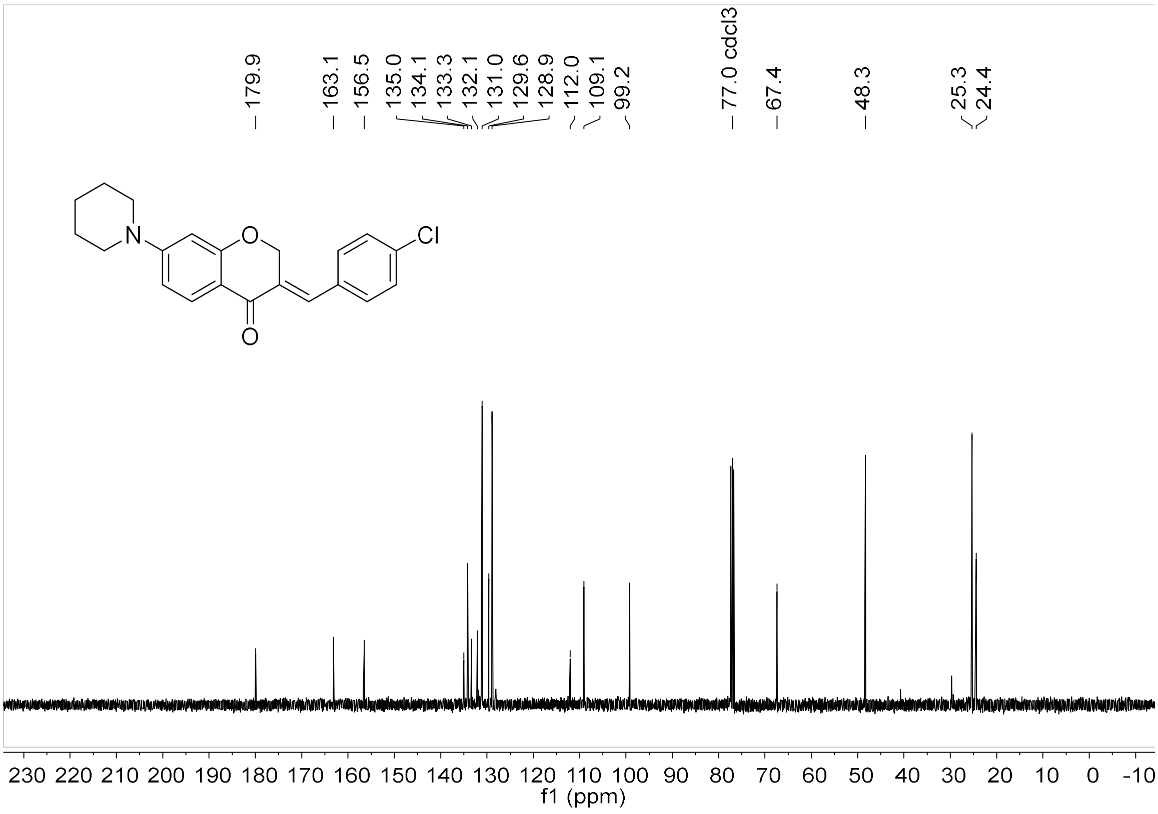

^{1}H NMR (500 MHz, $CDCl_3$) spectrum of 41

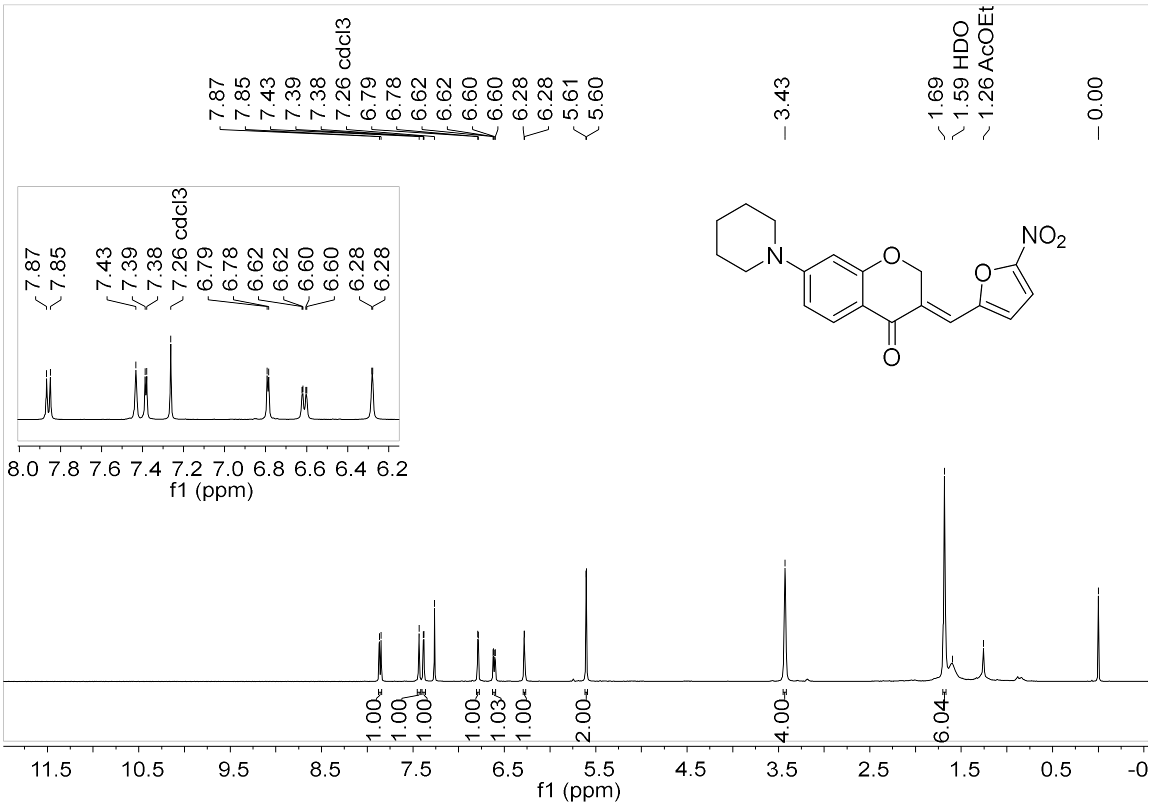

^{13}C NMR (126 MHz, $CDCl_3$) of 41

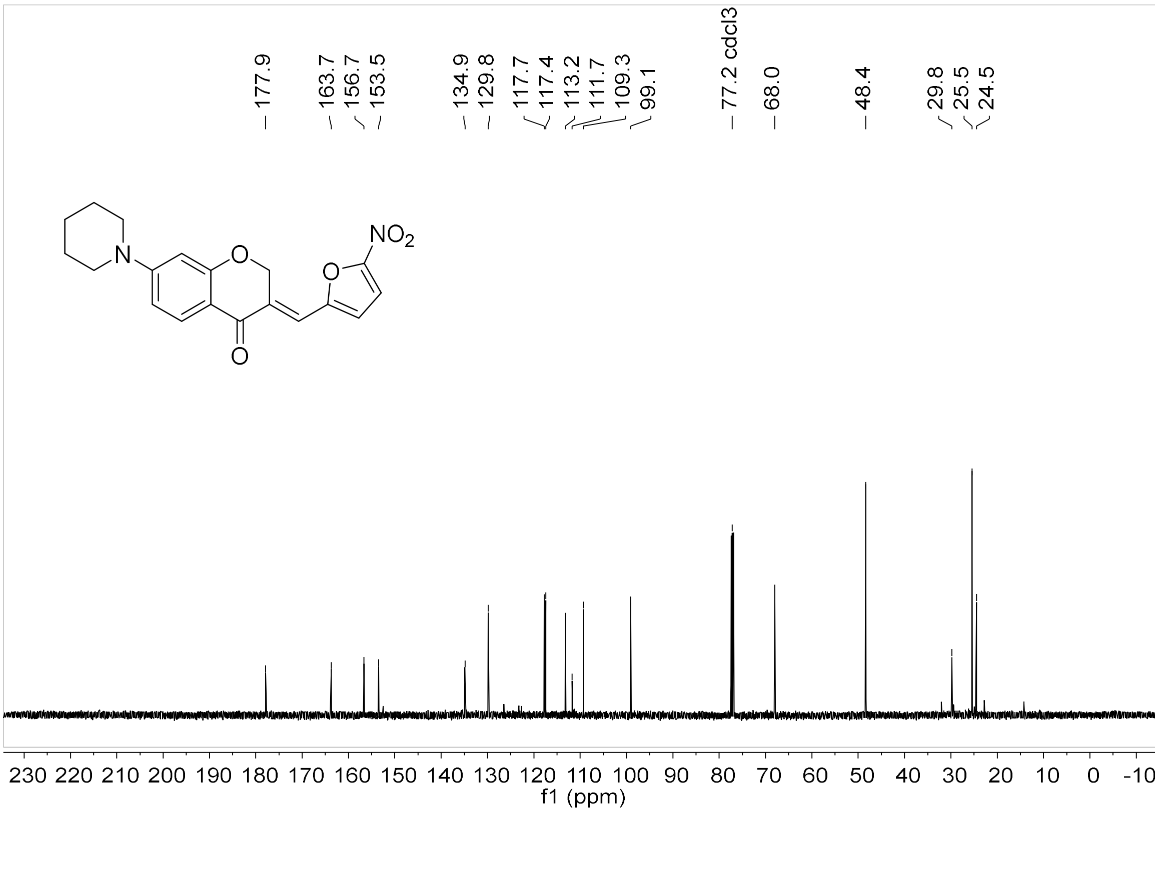

^{1}H NMR (500 MHz, CDCl₃) spectrum of 42

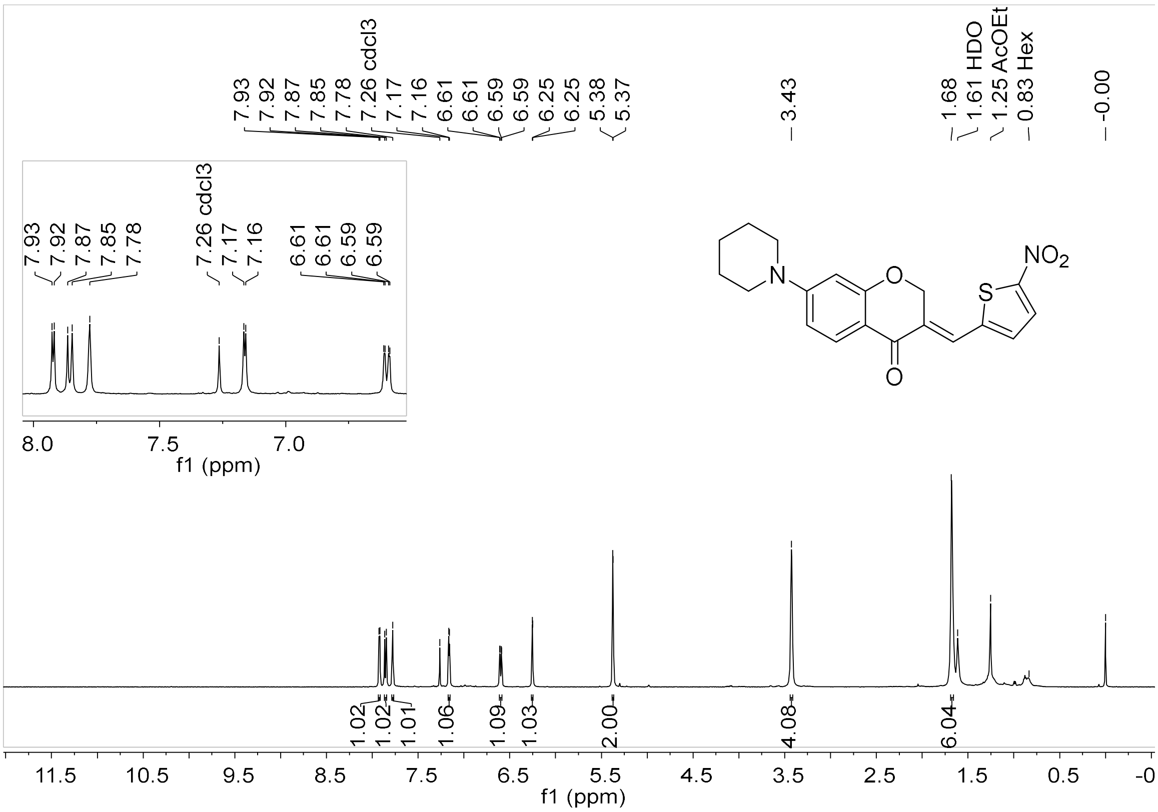

^{13}C NMR (126 MHz, CDCl₃) of 42

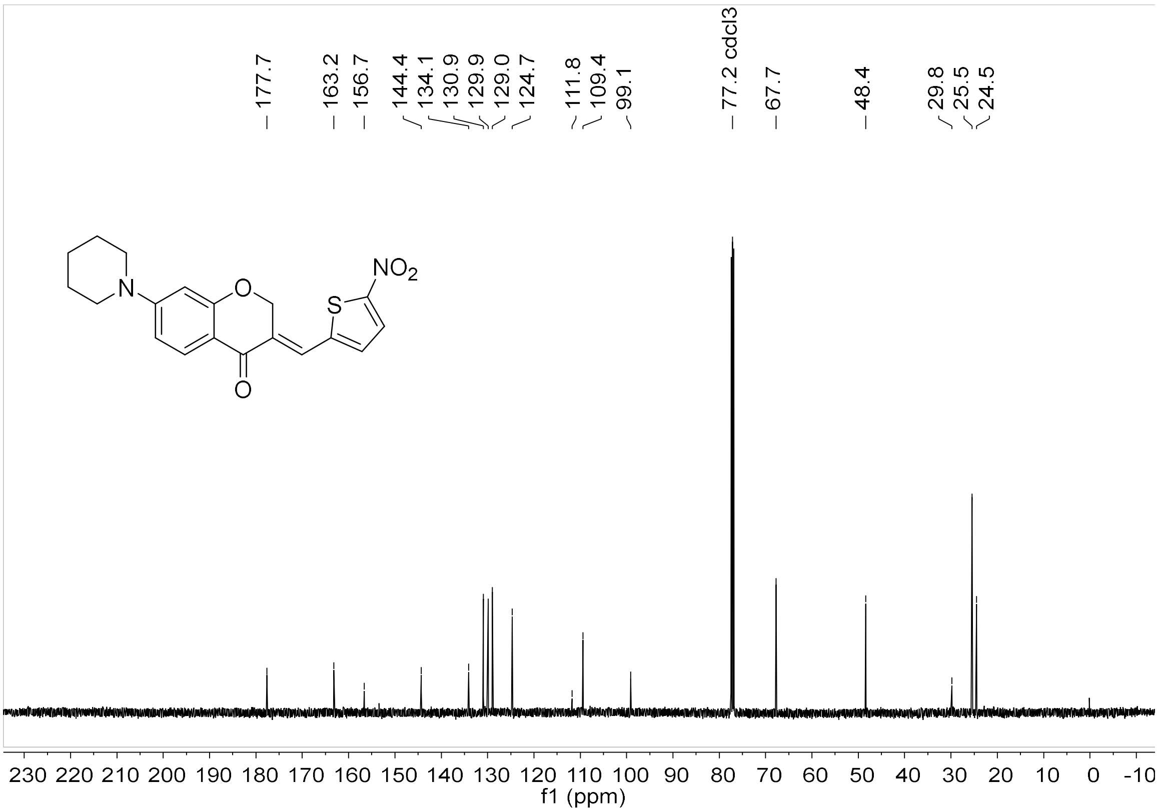

Supplement: Supplementary file 1 — Supplementary Material [file CMDC-20-e202500249-s001.pdf]
